# Supplementary material for: A Phase I Study of Carfilzomib with Cyclophosphamide and Etoposide in Relapsed and Refractory Leukemia and Solid Tumors
Source: Cancers (Basel). 2025 Sep 6;17(17):2924. doi: 10.3390/cancers17172924 (PMC12428389; doi:10.3390/cancers17172924)
Supplement: Supplementary file 1 [file cancers-17-02924-s001.zip › POE14-01 Protocol_Publication Supplement S2.pdf]

Pediatric Oncology Experimental Therapeutics Investigators' Consortium

Study Title: Phase I study of carfilzomib in combination with cyclophosphamide and etoposide for children with relapsed and refractory solid tumors and leukemias

Principal Investigators:

Norman J. Lacayo, MD
Stanford University School of Medicine and Stanford Cancer Institute
Lucile Packard Children's Hospital at Stanford
1000 Welch Rd., Ste. 300
Palo Alto, CA 94304
Phone: 650-723-5533
Fax: 650-723-5231
Email: lacayon@stanford.edu

Aru Narendran, MD, PhD
Alberta Children's Hospital
28 Oki Drive NW
Calgary, Alberta T3B 6A8
CANADA
Phone: 403-210-6418
Fax: 403-955-7684
Email: a.narendran@ucalgary.ca

Anne-Marie Langevin, MD
University of Texas Health Science Center at San Antonio
7703 Floyd Curl Drive, MC7810
San Antonio, TX 78229
Phone: 210-567-7460
Fax: 210-567-7466
Email: langevin@uthscsa.edu

Co-Investigators:

Valerie Brown, MD, PhD
Penn State Hershey Children's Hospital
500 University Drive, MC H085
Hershey, PA 17033-0850
Phone: 717-531-6012
Fax: 717-531-4789
Email: vbrown1@hmc.psu.edu

Steven DuBois, MD
Dana Farber Cancer Institute
450 Brookline Ave.
Boston, MA 02215
Phone: 617-632-5460
Fax: 617-632-5710
Email: steven_dubois@dfci.harvard.edu

Kevin Bielamowicz, MD
Arkansas Children's Hospital
1 Children's Way
Little Rock, AR 72202
Phone: 501-364-4405
Fax: 501-364-3654
Email: KJBielamowicz2@uams.edu

Tanya Trippett, MD
Memorial Sloan-Kettering Cancer Center
1275 York Ave.
New York, NY 10065
Phone: 212-639-8267
Fax: 212-639-3239
Email: trippet1@mskcc.org

Francis Eshun, MD
Phoenix Children's Hospital
1919 East Thomas Rd.
Phoenix, AZ 85016
Phone: 602-933-0921
Fax: 602-933-0211
Email: feshun@phoenixchildrens.com

POETIC Program Manager:

POETIC Data Coordinating Center
Pediatric Hematology/Oncology
Stanford University
455 Broadway Ave
Redwood City, CA 94063.
Phone: 650-736-0269
Fax: 650-736-3731
Email: poeticdcc@stanford.edu

Ativ Zomet, PhD.
Pediatrics Hematology/Oncology, Stanford University
POETIC DCC Research Program Manager
Phone: 650-497-0641
Fax: 650-736-3731
Email: azomet@stanford.edu

Research Pharmacist:

Namrata Patel, PharmD, BCOP
Pediatric Oncology/Hematology/SCT Clinical Pharmacist
Investigational Drug Services
Department of Pharmacy
Phone: 650.497.8776
Fax: 650.497.8283
Email: NaPatel@stanfordchildrens.org

Protocol version date: 01Mar2022

Declaration of the Study Principal Investigator

I affirm that the protocol has been appropriately developed and reviewed and is ready for approval.

Study Principal Investigator Name: Norman J. Lacayo, MD

Study Principal Investigator Signature: Norman J. Lacayo, MD

Electronically signed by: Norman J. Lacayo, MD
Reason: I have reviewed and am approving this document.
Date: Mar 4, 2022 11:25 PST

Date: Mar 4, 2022

Declaration of Site Principal Investigator

I confirm that I have read the above mentioned protocol and its attachments. I agree to conduct the described trial in compliance with all stipulations of the protocol, regulations and ICH E6 Guidelines for Good Clinical Practice.

Site Principal Investigator Name: ____

Site Principal Investigator Signature: ____

Date: ______________________________

PROTOCOL SYNOPSIS

TITLE: Phase I study of carfilzomib in combination with cyclophosphamide and etoposide for children with relapsed and refractory solid tumors and leukemias

OBJECTIVES:

Primary Objective:

To determine the DLTs and MTD of carfilzomib given in combination with cyclophosphamide and etoposide in pediatric patients with relapsed/refractory leukemias and solid tumors

Secondary Objectives:

1. To evaluate toxicities of carfilzomib in the pediatric population when combined with conventional chemotherapy
2. To gather preliminary efficacy data on the drug combination
3. To measure if circulating plasma proteasome (cProt) levels post treatment correlate with response to therapy and overall survival
4. To measure if the levels of proteasome activity and resistance to carfilzomib correlates with toxicity and/or response to treatment.
5. To measure if inhibition of proteasome activity by carfilzomib results in alteration in a number of autophagy and apoptosis related proteins, providing means to evaluate correlates of activity of carfilzomib.
6. To measure the level of proteasome inhibition in patient PBMCs before and during treatment by determination of the level of protein ubiquitination.
7. To determine *in vitro* sensitivity of patient leukemias and solid tumors to carfilzomib alone and in combination with study chemotherapeutic agents in order to generate a predictive model of drug sensitivity.
8. To perform whole exome sequencing (WES) and RNA seq on patient leukemia and solid tumor samples and WES on germ line DNA in order to determine potential mechanisms of drug sensitivity or resistance.

Exploratory Objectives:

1. To gather information on the methylation pattern of DNA and other epigenetic markers before and during treatment.
2. To evaluate potential biomarkers for measuring response and/or resistance to carfilzomib treatment.

STUDY DESIGN:

Disease Strata

Patients will be enrolled into Strata A or B, depending upon their underlying disease.

- Strata A – leukemia patients
- Strata B – solid tumor (including lymphoma) patients. If a lymphoma patient has ≥ 25% bone marrow blasts, then the patient will be considered a leukemia patient and will be enrolled in strata A. The dose escalations for each strata will be done independently

Treatment and Dose Escalation

Dose escalation will follow the rolling-six design. Doses will be escalated based upon DLTs until the MTD or the highest dose level is reached, whichever comes first. At the MTD or the highest dose level (if no MTD is reached), an additional 6 patients will be enrolled to further evaluate safety of the regimen. Once an MTD is determined for Strata A or B, if the Study Principal Investigator determines that the study treatment should not be futher pursued due to subject safety or enrollment barriers, the expansion cohort will be discontinued.

The initial dose escalation will utilize a 5-day dosing schedule of carfilzomib as described below

Dose Level	Cyclophosphamide mg/m^2	Etoposide mg/m^2	Carfilzomib mg/m^2
-2	See twice weekly dosing schedule (see below)		
-1	330	75	11
1	440	100	11
2	440	100	15
3	440	100	20
4	440	100	20/27*
5	440	100	20/36*

*For the FIRST CYCLE ONLY, each patient at dose levels 4 and 5 will receive 20 mg/m^2 on days 1 and 2 prior to escalation to $27 mg/m^2$ or 36 mg/m^2 as above for days 3-5. For all subsequent cycles, the patient will receive 27 mg/m2 or 36 mg/m2 as above, on all days 1-5.

The 3 drugs will be administered daily on days 1-5 schedule in the following order:

- Hour 0-1 Cyclophosphamide IV for 60 minutes (± 5 minutes)
- Hour 1-3 Etoposide IV for 120 minutes (± 10 minutes)
- Hour 3-3.5 Carfilzomib IV for 30 minutes (± 5 minutes)

Acute leukemia/NHL patients will receive a single dose of IT chemotherapy within 14 days of starting systemic therapy.

IF DOSE DE-ESCALATION IS REQUIRED BELOW DOSE LEVEL -1 OF THE 5-DAY DOSING SCHEDULE ABOVE (i.e. Dose level -1 is determined to be above

the MTD), THEN THE STUDY WILL MODIFY THE DOSE ESCALATION TO THE 2-DAY DOSING SCHEDULE OF CARFILZOMIB AS SHOWN BELOW.

Dose escalation of the 2-day schedule will begin at dose level 1.

Dose Level	Cyclophosphamide mg/m^2	Etoposide mg/m^2	Carfilzomib mg/m^2
-2	330	75	15
-1	330	75	20
1	330	75	20/27*
2	440	100	20/27*
3	440	100	20/36*
4	440	100	20/45*
5	440	100	20/56*

*For the FIRST CYCLE ONLY, each patient at dose levels 1 to 5 will receive 20 mg/m^2 on days 1 and 2 prior to escalation to 27mg/m^2, 36 mg/m^2, 45 mg/m^2, or 56 mg/m^2 as above for days 8, 9, 15, and 16. For all subsequent cycles, the patient will receive 27 mg/m^2, 36 mg/m^2, 45 mg/m^2, or 56 mg/m^2 as above on days 1, 2, 8, 9, 15, and 16.

Cyclophosphamide and Etoposide will be administered daily on days 1-5

Carfilzomib will be administered on days 1, 2, 8, 9, 15, 16

Cyclophosphamide and Etoposide daily on Days 1-5
  Hour 0-1 Cyclophosphamide IV for 60 minutes (± 5 minutes)
  Hour 1-3 Etoposide IV for 120 minutes (± 10 minutes)

Carfilzomib on Days 1, 2, 8, 9, 15, 16
  On Days 1,2 – Carfilzomib IV given from Hour 3-3.5 for 30 minutes (± 5 min)
  On Days 8,9,15,16 - Carfilzomib IV given alone over 30 min (± 5 min)

Acute leukemia/NHL patients will receive IT as above.

Patients with BSA > 2.2 m^2 will be dosed for Etoposide, Cyclophosphamide, and Carfilzomib based upon a BSA of 2.2 m^2.

Patients <10 kg will be dosed for Etoposide, Cyclophosphamide, and Carfilzomib based upon weight in lieu of BSA. The dose per kg used will be the dose per m^2 divided by 30.

All patients on either dosing schedule will start granulocyte colony-stimulating factor on Day 6 of each cycle, between 24-36 hours after completion of carfilzomib administration. This can be in the form of filgrastim (Neupogen®) or pegfilgrastim (Neulasta®).

For their first cycle, all patients will be pre-medicated for Carfilzomib with dexamethasone 0.1 mg/kg (max 4 mg).

Chemotherapy Cycles

Each cycle will be at least 28-days long. The patient must receive a minimum of one cycle. If a patient shows response to therapy, has recovered from the toxicities of therapy, and meets the same laboratory and organ function inclusion and exclusion criteria used for initial enrollment (ECHO/EKG need not to be repeated unless clinically indicated), the patient will be permitted to receive an additional cycle. There is no maximum number of cycles a patient can receive.

If a patient experiences a DLT and is fully recovered and has shown a response to therapy, then a single dose de-escalation will be permitted for subsequent cycles. If the patient has another AE that would be considered a DLT, then the patient will be taken off protocol.

There will be no intra-patient dose escalation.

Disease Assessment

For leukemia patients, disease assessments will be performed after every cycle.

For solid tumor/lymphoma patients, disease assessment will be performed after cycles 2, 4, 6, then a minimum of q3 cycles.

STUDY POPULATION: Relapsed and refractory solid tumors and leukemias

INCLUSION CRITERIA: Subjects must meet all of the following inclusion criteria to be eligible to enroll in this study.

Disease-related:

1. Patients must have either of the following:
   a. Relapsed/refractory leukemia in 2nd or greater relapse or who have failed at least one re-induction attempt after relapse or for refractory disease. Patients must meet the WHO classification with ≥ 5% blasts in the bone marrow or must have definitive extramedullary disease (e.g. chloromas, skin lesions). Patients may have asymptomatic CNS 1 or CNS 2 disease, but not CNS 3 or symptomatic CNS disease.

      OR

b. Relapsed/refractory non-CNS solid tumor that has not responded or has relapsed and for which no standard treatment is available. Patients may not have primary CNS tumors or CNS metastases. Lymphoma patients are permitted. Patients do not need to have measurable disease.

Demographic:

2. Age 6 months - 29.99 years at enrollment
3. Life expectancy ≥ 3 months
4. Lansky or Karnofsky ≥50
5. Prior therapy
    a. Patient must have fully recovered from the acute toxic effects of all prior chemotherapy, immunotherapy, radiotherapy, or surgery prior to study entry.
    b. Myelosuppressive therapy- At least 14 days must have elapsed since the administration of previous therapy. Six weeks must have elapsed from the administration of nitrosoureas or mitomycin C. For patients with ALL on maintenance therapy, they may be eligible if 7 days have elapsed and they are recovered from the toxic effects of the chemotherapy. This restriction does not include intrathecal chemotherapy, which is permitted. Hydroxyurea is permitted but must be discontinued ≥24 hours prior to start of protocol therapy.
    c. Biologic agents- At least 14 days must have elapsed since the completion of therapy with a biologic agent such as a monoclonal antibody. Seven days must have elapsed since the last dose of retinoids
    d. Radiation therapy – At least 14 days must have elapsed for local XRT. At least 90 days must have elapsed if prior radiation to ≥50% of the pelvis, the spine, or other substantial bone marrow radiation including TBI.
    e. Hematopoietic growth factors- At least 7 days must have elapsed since the last dose of G-CSF or GM-CSF. At least 14 days must have elapsed since last dose of pegfilgrastim (Neulasta®).
6. Patient must be ≥ 3 months from hematopoietic stem cell transplant, must not have active GVHD, and must be off all immunosuppression

Laboratory

7. Organ function:
    a. Either a serum creatinine ≤ ULN for age, or calculated or measured GFR ≥ 70 mL/min/1.73 m^2
    b. Total bilirubin ≤ 1.5 x ULN for age, direct bilirubin ≤ ULN for age
    c. AST and ALT ≤ 3 x ULN for age unless elevation can be clearly attributed to liver leukemia or metastases
    d. ECHO shortening fraction ≥ 27%
    e. Pulse Oximetry measurement ≥ 95% saturation without supplemental oxygen
8. Bone marrow function
    a. Hgb ≥10 g/dL – can be transfused

b. Plts ≥ 75,000 – cannot be transfused (must be ≥ 7 days from last plt transfusion)
c. ANC ≥ 750 – cannot be transfused (must be ≥ 72 hours from last neutrophil infusion)
However, the plt and ANC requirements can be waived if low counts thought to be secondary to leukemia or tumor bone marrow infiltration

Ethical/Other

9. Reproductive function:
    a. Female patients of childbearing potential must have a negative serum pregnancy test confirmed within 7 days prior to enrollment
    b. Female patients with infants must agree not to breastfeed their infants while on the study
    c. Male and female patients of child-bearing potential must agree to use an effective method of contraception approved by the investigator during the study and for a minimum of 3 months after study treatment
10. Written informed consent

EXCLUSION CRITERIA:

Concurrent Conditions

1. Prior treatment with carfilzomib
2. Known allergy to Captisol® (a cyclodextrin derivative used to solubilize carfilzomib).
3. Down syndrome
4. Fanconi Anemia or other underlying bone marrow failure syndrome
5. Pregnant or lactating females
6. Known history of Hepatitis B or C or HIV
7. Patient with any significant concurrent illness
8. Patient with uncontrolled systemic fungal, bacterial, viral or other infection with ongoing signs/symptoms despite appropriate treatment
9. Patient with illness, psychiatric disorder or social issue that could compromise patient safety or compliance with the protocol treatment or procedures, or interfere with the consent, study participation, follow-up, or interpretation of study results.

PROCEDURES: Radiologic evaluation and/or bone marrow aspiration as indicated for primary disease assessment

STUDY TREATMENT:

Carfilzomib
Cyclophosphamide
Etoposide
IT Methotrexate or Cytarabine for acute leukemia/NHL patients

**PRIMARY ENDPOINT:**	MTD
**SECONDARY EN POINTS:**	1. Safety/Toxicity<br>2. Efficacy<br>3. Biologic correlates
**STATISTICAL METHODS:**	Dose escalation by Rolling-Six design<br><br>Response Criteria<br>Solid Tumors – RECIST criteria (version 1.1)<br>Lymphoma – Cheson criteria<br>Leukemia – Standard response criteria

TABLE OF CONTENTS

PROTOCOL SYNOPSIS **5**

TABLE OF CONTENTS **12**

1. INTRODUCTION **18**

1.1 Disease Specific Background 18

1.2 Proteasome Background 18

1.3 Carfilzomib Background 18

1.4 Dose Rationale 23

1.5 Pediatric Study Rationale 24

2 OBJECTIVES **34**

2.1 Primary Objective 34

2.2 Secondary Objectives 34

2.3 Exploratory objectives 34

3 EXPERIMENTAL PLAN **35**

3.1 Study Design 35

3.2 Number of Centers 38

3.3 Number of Subjects 38

3.4 Estimated Study Duration 38

4 SUBJECT SELECTION **39**

4.1 Inclusion Criteria 39

4.2 Exclusion Criteria 41

4.3 CNS Status Definitions 41

5 SUBJECT ENROLLMENT **43**

5.1 Research Participant Registration 43

6 TREATMENT PROCEDURES **45**

6.1 Drug Preparation and Administration 45

6.2 Definition of Dose-limiting Toxicity (DLT) 45

6.3 Dose Reductions/Adjustments 47

6.4 Safety Considerations 48

6.5 Concomitant Medications 49

6.6 supportive care 50

7 STUDY TESTS AND OBSERVATIONS **52**

7.1 Blood Correlative Studies Time Points....53

8 STUDY DISCONTINUATION 54

8.1 Criteria for Removal from Protocol Therapy....54

8.2 Off Study Criteria....54

8.3 Termination of the Study by POETIC....54

9 ADVERSE EVENTS 55

9.1 Adverse Events Definitions 55

9.2 Causality 56

9.3 Adverse Events Reporting Procedures....56

9.4 Serious Adverse Events Definitions 57

9.5 Serious Adverse Event Reporting and Documentation Requirements 57

9.6 Expedited Reporting 60

9.7 Pregnancy....62

10 STATISTICAL ANALYSIS 63

10.1 Study Design....63

10.2 Study Endpoints....79

10.3 Sample Size Considerations....79

10.4 Interim Analysis....79

10.5 Planned Methods of Analysis 79

10.6 Data and Safety Monitoring....79

11 INVESTIGATIONAL PRODUCT - CARFILZOMIB 81

11.1 Description....81

11.2 Formulation....81

11.3 DRUG SUPPLY 82

11.4 Storage 83

11.5 Accountability....83

11.6 TOXICITY....83

12.1 CYCLOPHOSPHAMIDE INJECTION (CYTOXAN) NSC #26271 87

12.1.2 Formulation: 87

12.1.3 DRUG SUPPLY: 87

12.1.4 STORAGE:....87

12.1.5 TOXICITY....88

12.2 ETOPOSIDE INJECTION (VEPESID®, ETOPOPHOS®, VP-16) NSC#141540.89

12.2.2 Formulation: 89
12.2.3 DRUG SUPPLY: 90
12.2.4 STORAGE: 90
12.2.5 TOXICITY 90
12.3 CYTARABINE (CYTOSINE ARABANOSIDE, ARA C, CYTOSAR® NSC #63878)91
12.3.2 Formulation: 91
12.3.3 DRUG SUPPLY: 92
12.3.4 STORAGE: 92
12.4 METHOTREXATE (MTX, AMETHOPTERIN, TREXALL®) NSC#000740 93
12.4.2 Formulation: 93
12.4.3 DRUG SUPPLY: 94
12.4.4 STORAGE: 94
13 REGULATORY OBLIGATIONS 96
13.1 Informed Consent 96
13.2 Compliance with Laws and Regulations 96
13.3 Pre-study Documentation Requirements 97
13.4 Subject Confidentiality 98
14 ADMINISTRATIVE AND LEGAL OBLIGATIONS 99
14.1 Protocol Amendments, IRB Correspondence, and Study Termination 99
14.2 Study Documentation and Archive 100
14.3 Study Monitoring and Data Collection 101
15 REFERENCES 103
APPENDIX A: NCI-CTCAE VERSION 4.03 106

LIST OF ABBREVIATIONS

Abbreviation	Definition
°C	degrees Centigrade
°F	degrees Fahrenheit
AE	adverse event
ALL	acute lymphoblastic leukemia
ALT	alanine aminotransferase
AML	acute myeloid leukemia
ANC	absolute neutrophil count
aPTT	activated partial thromboplastin time (also PTT)
ALT	alanine aminotransferase
ASaT	All Subjects as Treated
AST	aspartate aminotransferase
BID	twice daily
BSA	body surface area
BUN	blood urea nitrogen
CBC	complete blood count
CFR	Code of Federal Regulations
CHF	congestive heart failure
CR	complete response
CRi	complete response with incomplete count recovery
CRp	complete response with incomplete platelet recovery
CrCl	creatinine clearance
CRF	case report form(s)
CRO	clinical research organization
CSF	cerebrospinal fluid
CSR	Clinical Study Report
CT	computed tomography
CTCAE	Common Terminology Criteria for Adverse Events
CV	curriculum vitae
dL	deciliter
DLT	dose-limiting toxicity
DOR	duration of response
DVT	deep venous thrombosis
ECG	electrocardiogram
ECOG	Eastern Cooperative Oncology Group
FAS	Full Analysis Set
FCBP	Females of childbearing potential
FDA	Food and Drug Administration

FISH	fluorescent in situ hybridization
FLC	free light chain
G-CSF	granulocyte colony stimulating factor
GCP	Good Clinical Practice
GLP	Good Laboratory Practice
GM-CSF	granulocyte macrophage colony stimulating factor
GVHD	graft vs. host disease
h	hour(s)
hgb	hemoglobin
HIPAA	Health Insurance Portability and Accountability Act
HIV	human immunodeficiency virus
HVA	homovanillic acid
IB	Investigator Brochure
ICH	International Conference on Harmonisation
IEC	Independent Ethics Committee
IND	Investigational New Drug (Application)
INR	International Normalized Ratio
IRB	Institutional Review Board
IT	intrathecal
IV	intravenous
kg	kilogram(s)
LDH	lactate dehydrogenase
LP	lumbar puncture
mg	milligram(s)
MIBG	metaiodobenzylguanidine scan
min	minute(s)
mIU	milli International Units
mL	milliliter(s)
MM	multiple myeloma
mm^2	millimeter(s) squared
mm^3	millimeter cubed
MPAL	mixed phenotype acute leukemia
MR	minimal response
MRI	magnetic resonance imaging
MTD	maximum tolerated dose
NCI	National Cancer Institute
NHL	non-Hodgkin's lymphoma
ORR	overall response rate
PBMC	peripheral blood mononuclear cells
PD	progressive disease
PET	positron emission tomography

PFS	progression-free survival
PK	Pharmacokinetics
plt	platelet
PO	per os (oral)
PR	partial response
PSA	prostate-specific antigen
PT	prothrombin time
PTT	partial thromboplastin time
QDx5	daily dosing for five days
QIU	Qualified Investigator Undertaking Form
RBC	red blood cell
SAE	serious adverse event
SAP	Statistical Analysis Plan
sCR	stringent complete response
SD	stable disease
SEER	Surveillance, Epidemiology, and End Results
SPEP	serum protein electrophoresis
STD_{10}	severely toxic dose in 10% of animals
TLS	tumor lysis syndrome
TTP	time to tumor progression
U	unevaluable
ULN	upper limit of the normal range
UPEP	urine protein electrophoresis
VGPR	very good partial response
VMA	vanillylmandelic acid
WBC	white blood count

1. INTRODUCTION

1.1 Disease Specific Background

1.2 Proteasome Background

The proteasome is a multicatalytic proteinase complex that is responsible for degradation of a wide variety of protein substrates within normal and transformed cells. Intracellular proteins targeted for degradation by the proteasome are first ubiquitinated via the ubiquitin conjugation system. Ubiquitinated proteins are cleaved within the proteasome by one or more of three separate threonine protease activities: a chymotrypsin-like activity, a trypsin-like activity, and a caspase-like activity.

1.3 Carfilzomib Background

Carfilzomib (PR-171) is a tetrapeptide ketoepoxide-based inhibitor specific for the chymotrypsin-like active site of the 20S proteasome. Carfilzomib is structurally and mechanistically distinct from the dipeptide boronic acid proteasome inhibitor bortezomib (Velcade®). In addition, when measured against a broad panel of proteases including metallo, aspartyl, and serine proteases, carfilzomib demonstrated less reactivity against non-proteasomal proteases when compared to bortezomib[1, 2].

1.3.1 Carfilzomib Toxicology Studies

In the initial Good Laboratory Practice (GLP)-compliant toxicity studies done by the drug maker, Onyx, carfilzomib was administered to rats and monkeys as two complete two-week cycles of QDx5 for five days with nine days rest[3]. Administration to rats at 12 mg/m^2, the severely toxic dose in 10% of animals (STD_{10}), caused > 90% proteasome inhibition in red blood cells one hour after dosing. Overall, stronger inhibition of the proteasome and longer duration of inhibition was tolerated with carfilzomib compared with bortezomib. Daily administration of bortezomib at anti-tumor doses is not tolerated in animals, and therefore daily bortezomib has not been given in the clinic. A dose-dependent decrease in proteasome activity was demonstrated in animals, and equivalent levels of proteasome inhibition were achieved with administration of carfilzomib as either an intravenous (IV) push or an IV infusion. The dose-limiting toxicities (DLTs) of carfilzomib in both the rat and monkey 28-day GLP toxicity studies included toxicity to the gastrointestinal tract, bone marrow, pulmonary, and cardiovascular systems. No behavioral or histopathological signs of neurotoxicity were observed, and carfilzomib does not cross the blood-brain barrier.

In 6-month rat and 9-month chronic toxicity studies, carfilzomib was administered on Days 1, 2, 8, 9, 15, and 16 of a 28-day cycle, mimicking the active anti-tumor regimen being used in ongoing adult Phase II studies in myeloma and solid tumors[3]. Tolerability was excellent, with no evidence of peripheral (or central) neurotoxicity, including neuropathology, observed, even at high doses. This is in stark contrast to that observed with bortezomib.[4,5] DLTs included effects on the gastrointestinal, renal, pulmonary, and cardiovascular systems and appeared to be related to Cmax effects. Of note, neutropenia was not observed; rather, transient neutrophilia was seen following acute dosing. Renal, cardiovascular and gastrointestinal toxicities were similar to those observed with bortezomib. Finally, cyclical thrombocytopenia, likely due to inhibition of platelet budding

from megakaryocytes, was similar to that seen with bortezomib. Proteasome inhibition in the blood in excess of 90% was achievable at well-tolerated doses, which contrasts with the ~70% proteasome inhibition achievable with bortezomib at its maximum tolerated dose (MTD). In summary, these animal toxicity studies support the tolerability of carfilzomib in clinical studies, even on intensive dosing schedules and at doses achieving proteasome inhibition in excess of what can be achieve with bortezomib at its MTD on a less intensive schedule.

1.3.2 Carfilzomib Preclinical Antitumor Activity

Based upon the results of *in vitro* and *in vivo* studies, it is anticipated that the more intense and longer duration of proteasome inhibition that can be achieved with carfilzomib will result in enhanced anti-tumor activity relative to bortezomib. Continuous (72 hr) exposure to carfilzomib is associated with potent cytotoxic and pro-apoptotic activity across a broad panel of tumor-derived cell lines in culture[1,6]. Incubation of hematologic tumor cell lines with carfilzomib for as little as one hour leads to rapid inhibition of proteasome activity followed by accumulation of polyubiquitinated proteins and induction of apoptotic cell death. Carfilzomib has also been demonstrated to be cytotoxic in bortezomib-resistant tumor cell lines[1,6].

The anti-tumor efficacy of carfilzomib has been tested in immunocompromised mice implanted with a variety of tumor cell lines. In a human colorectal adenocarcinoma model HT-29, administration of carfilzomib on a twice-weekly Day 1, Day 2 schedule resulted in significant reduction in tumor size and was superior to a twice-weekly Day 1, Day 4 schedule using the same dose of carfilzomib, and a once-weekly dosing schedule using twice the dose level. Bortezomib at its MTD has no activity in this xenograft model using the standard Day 1, Day 4 schedule[1].

1.3.3 Phase 1 Experience With Carfilzomib As A Monotherapy

The initial single agent Phase I clinical trial of carfilzomib, PX-171-001, evaluated carfilzomib in subjects with relapsed or refractory hematologic malignancies.[7] Eight dose levels of 3-6 patients each received 5 consecutive days of carfilzomib IV push at doses of 1.2, 2.4, 4, 6, 8.4, 11, 15, and 20 mg/m2, given in 14-day cycles. A total of 29 patients were treated. No dose limiting toxicities (DLTs) were observed in the first 7 dose levels through 15 mg/m^2. At 20 mg/m^2, there were 2 DLTs - grade 3 febrile neutropenia and grade 4 thrombocytopenia. Therefore, the maximum tolerated dose (MTD) was determined to be 15 mg/m^2/day. The most common non-hematologic toxicities, primarily grade 1 and 2, included fatigue, nausea, and diarrhea. No grade 3 or 4 peripheral neuropathies were reported. Drug clearance was rapid with an elimination half-life less than 30 minutes. Proteasome inhibition of >75% was seen in whole blood and PBMCs after a single dose of carfilzomib at dose levels of 15 mg/m^2 and higher. Antitumor activity was seen at doses ≥11 mg/m^2, including in 1 Mantle Cell Lymphoma patient (unconfirmed CR), 2 Multiple Myeloma patients (PR and minimal response), and one patient with Waldenström's Macroglobulinemia (minimal response). Across all dose cohorts, nine patients displayed stable disease with an average duration on therapy of 8 cycles. The most common reason for treatment discontinuation was progressive disease. However, for patients who completed at least 6 cycles, the primary reason for treatment discontinuation was the difficulty in maintaining the dosing schedule of 5 days per week every 14 days. As a result, the decision was made to move forward in subsequent adult studies with the easier dosing schedule of twice weekly therapy in a 28-day cycle as described below.

A Phase 1 clinical trial, PX-171-002, tested carfilzomib in subjects with relapsed/refractory hematologic malignancies.[8] During the dose escalation portion of the trial, 36 subjects received carfilzomib on Days 1, 2, 8, 9, 15, and 16 of a 28-day cycle. Subjects with Multiple Myeloma (MM), Non-Hodgkin's Lymphoma (NHL), Waldenström's Macroglobulinemia, and Hodgkin's Lymphoma (HL) were enrolled on the study.

No DLTs were observed in the initial seven cohorts (doses ranged from 1.2 to 15 mg/m^2) of three subjects each. At the 20 mg/m^2 dose level, one of eight patients had a Grade 3 renal failure at Cycle 1, Day 2 which was considered possibly related to study drug and lasted for six days. The patient continued on study for the remainder of Cycle 1 before having disease progression. At the 27 mg/m^2 dose level, one of six subjects experienced a DLT during Cycle 1, consisting of severe hypoxia with pulmonary infiltrates following Day 2 of dosing. In subjects where the 27 mg/m^2 dose was efficacious, a "first dose effect" was seen that included a constellation of findings that appeared to be the clinical sequelae of rapid tumor lysis syndrome (TLS) and/or cytokine release. This effect was notable for fever, chills, and/or rigors occurring during the evening following the first day of infusion. On the second day, three of five subjects with multiple myeloma experienced an increase in creatinine to Grade 2 (including the subject with the DLT). This elevation was rapidly reversible and all three subjects were rechallenged with carfilzomib without recurrence of the events. Interestingly, all three subjects had a rapid decline in serum and/or urine M-protein levels; two subjects achieved a PR and the third subject achieved a minimal response (MR). There were no consistent changes in potassium, calcium, phosphorous, or uric acid levels although some increases in LDH and other markers of tumor lysis were noted. Because of the possible TLS and reversible creatinine elevations, hydration and very-low dose dexamethasone prophylaxis were instituted in subsequent studies and have essentially eliminated clinically significant TLS/creatinine elevations and the other "first-dose effects."

Hematologic toxicities were primarily mild or moderate. The thrombocytopenia reported with carfilzomib is cyclical and similar to that reported with bortezomib. The cause and kinetics of the thrombocytopenia following treatment are different from those of standard cytotoxic agents.

Of the 36 evaluable patients enrolled in PX-171-002, 20 had MM[8]. Four MM patients achieved a partial response (PR), one of two at the 15 mg/m^2 dose, one of six at the 20 mg/m^2 dose, and two of five at the 27 mg/m^2 dose. The responses have been rapid in onset, beginning in some subjects after 1-2 doses. The duration of response (DOR) ranged from 134 to 392 days. The minimal effective dose was 15 mg/m^2 wherein >80% proteasome inhibition in peripheral blood and mononuclear cells was observed one hour after dosing. The median number of prior therapies for subjects on this trial was five, and responses were seen in subjects who had relapsed from (including some refractory to) bortezomib and/or immunomodulatory agents. Stable disease also occurred in four NHL and five MM subjects, with subjects on therapy for up to 409 days. Such prolonged therapy, at "full" twice-weekly doses, is not possible with bortezomib. These results led to the initiation of two Phase 2 studies.

1.3.4 Phase 2 Experience With Carfilzomib As A Monotherapy

Two Phase 2 clinical studies were completed with carfilzomib in MM patients, PX-171-003-A0 (N=46) in relapsed and refractory MM and PX-171-004 (N=164) in relapsed MM. In both studies,

patients were initially dosed with 20 mg/m^2 on Days 1, 2, 8, 9, 15, and 16 on a 28-day schedule. In these studies there were four cases of suspected or documented TLS prior to institution of the prophylaxis guidelines. Since these guidelines were implemented, no further cases of TLS have been reported including in >350 additional patients with relapsed or refractory MM treated in ongoing Phase II studies. In both studies, the most common adverse events were fatigue, anemia, thrombocytopenia (primarily cyclical), gastrointestinal, and dyspnea. Almost all were Grades 1 or 2. There were reported cases of increased serum creatinine that were primarily < Grade 2 and were transient, rapidly reversible, and non-cumulative. A very low rate of treatment-emergent peripheral neuropathy, 2.2% Grade 3/4, was observed in PX-171-003-A0 despite the fact that 78% of patients had Grade 1/2 neuropathy upon study entry[9].

The response rate in PX-171-003-A0 was 18% PR, 7% MR and 41% SD in these patients that entered the study with progressive disease and were refractory to their most recent therapy, often including bortezomib and/or an immunomodulatory drug (usually lenalidomide). The median time to progression on the PX-171-003-A0 study was 5.1 months with a DOR of 7.4 months (mean follow up of 7.6 months) [9].

A "stepped up" dosing schedule, referred to as 20/27 mg/m^2, has subsequently been incorporated into the PX-171-003 study (referred to as PX-171-003-A1) in order to maximize the clinical benefit of carfilzomib. Patients received 20 mg/m^2 for the first cycle and 27 mg/m^2 thereafter. The study completed enrollment of 266 patients by the end of 2009. To date, this dosing schedule has been well tolerated[7]. An independent Safety Oversight Group (SOG) evaluated the safety data from the 40 of 250 patients to be enrolled on the 20/27 schedule and agreed that the trial should proceed without modification. No cases of TLS were observed and rates of BUN and creatinine elevation dropped sharply, with Grade 3/4 renal impairment dropping to 2.2% in A1(from 15% in A0), most likely due to hydration and very low dose dexamethasone. The other most common adverse events were similar to the A0 portion of the study. Treatment-emergent peripheral neuropathy remains low on this portion of the study with 15% Grade 1/2 and one (0.7%) Grade 3/4 event reported to date on PX-171-003-A1[8]. In addition, anemia rates in the PX-171-003-A1 (higher dose) were lower than those reported in the PX-171-003–A0 portion of the study, possibly indicating that the higher dose of carfilzomib is achieving better clearing of neoplastic cells in the bone marrow allowing superior normal marrow reconstitution. Rates of thrombocytopenia and neutropenia were similar in the two cohorts, with Grade 3 neutropenia in ~5% without any Grade 4 neutropenia to date.[9]

In PX-171-004, a first cohort of patients received 20 mg/m^2. The subset of patients (N=54) that had not seen bortezomib had an ORR of 46% (2% CR, 9% VGPR and 35% PR), while the bortezomib treated patients (N=33) had an ORR of 18% (3% CR, 3% VGPR and 12% PR) [10,11]. The median TTP was 7.6 and 5.3 months in these two groups, respectively. Thus, carfilzomib can induce very high levels of response in patients who have not previously been treated with bortezomib and, even in bortezomib-treated patients, substantial anti-tumor activity is observed. Of note, disease control (PR + MR + SD) was achieved in ~65% of patients with progressive MM entering the study. Patients on these studies have been treated for >12 cycles with good tolerability and no cumulative toxicity (e.g., bone marrow, severe fatigue, or neuropathy) have not been observed.

The protocol was amended to allow patients to increase to 27 mg/m^2 in Cycle 2 or later based on tolerability, similar to that used in PX-171-003 – A1.

Further information about the Phase 2 studies is presented in the Investigator's Brochure.

1.3.5 Experience With Carfilzomib In Combination With Lenalidomide And Dexamethasone

PX-171-006 is an ongoing Phase 1b study in patients with relapsed multiple myeloma in which carfilzomib is administered in combination with lenalidomide (Revlimid®) and dexamethasone. "Low-dose" dexamethasone 40 mg/day is given on Days 1, 8, 15, and 22 in all cases. Carfilzomib is administered IV on Days 1, 2, 8, 9, 15, and 16; lenalidomide is administered PO on Days 1 through 21.

Enrollment has closed in this study, and no MTD was reached. The maximum per protocol doses of carfilzomib (27mg/m^2) with lenalidomide 25mg and low dose dexamethasone are being used[12]. After 8 patients tolerated these doses well, an additional 44 patients were enrolled in an "expansion" cohort at this level, and this regimen is being taken into Phase III in study PX-171-009.

To date, 40 patients were treated in cohorts 1-6 and 44 in the cohort 6 expansion. 27/32 patients in cohorts 1–5 are evaluable for safety and 29/32 for response. Patients were heavily pre-treated; 72% received prior BTZ and 87.5% received prior LEN or thalidomide (Thal). 47% of patients were refractory to their last therapy (typically lenalidomide + high dose dexamethasone; > 84% of patients had a history of neuropathy with 67% BTZ- or Thal-related. No treatment emergent fatigue, neuropathy, or thrombotic events ≥ Grade 3 were observed. Hematological AEs ≥ Grade 3 (thrombocytopenia [n=6], anemia [n=4], and neutropenia [n=6]) were reversible. 4 patients had drug-related SAEs as follows: transient Grade 3 sinus bradycardia, Grade 3 upper respiratory tract infection, febrile neutropenia, and Grade diarrhea + Grade urinary infection. ORR and CBR for the 29 evaluable patients are 59% and 72%, respectively. Response data is shown in the table below. Initial responses improved with continued therapy, (up to 18 cycles). Median duration of response has not been reached (median follow-up 5.2 months). No dose-limiting toxicities or deaths attributed to study treatment have been observed. Several patients have completed the study (in the lower dose cohorts) after 18 cycles and are continuing in an extension study. Updated efficacy data are presented in the following table:

	**CRd: Cohorts 1–5 (CFZ: 15 to 20 mg/m2; LEN: 10 to 25 mg)**		
**Response**	**Relapsed (n=16)**	**Refractory (n=13)**	**Overall (n=29)**
≥ CR/nCR	5 (31)	1 (8)	6 (21)
≥ VGPR	7 (44)	4 (31)	11 (38)
≥ PR	9 (56)	8 (62)	17 (59)
≥ MR	11 (67)	10 (77)	21 (72)

Together, these results suggest that carfilzomib, lenalidomide, and low-dose dexamethasone (CRd) in combination are active and well tolerated and that there are no significant overlapping toxicities (in the dose ranges tested). Importantly, lenalidomide-associated neutropenia and thrombocytopenia do not appear to be exacerbated by concurrent treatment with carfilzomib, even up to 27mg/m^2, suggesting that carfilzomib will combine well with other anti-cancer agents.

1.4 DOSE RATIONALE

Preliminary data suggest that carfilzomib as a single agent can produce substantial response rates in myeloma subjects across a variety of dosing cohorts. Responses were seen over a wide therapeutic window, from 15 to 27 mg/m^2. Maximum proteasome inhibition was seen at doses 11 mg/m^2 and higher in whole blood samples taken 1 hour after the first dose. The final analysis of the human pharmacokinetic (PK) data is ongoing but appears to be rapid and similar to the results from the animal studies. Carfilzomib is rapidly cleared from plasma with an elimination half-life of < 60 minutes at the 20 mg/m^2 dose. Large, single arm studies of the 20/27 mg/m^2 dose are ongoing and suggest that this dose is very well tolerated with patients being treated for >10 cycles without cumulative toxicities

In multiple preclinical studies, the tolerability of carfilzomib in rats has been shown to be significantly higher when administered as a 30 min infusion as compared to a rapid IV bolus. Toxicities observed with IV bolus injection of carfilzomib *above the MTD* at a dose of 48 mg/m^2 include evidence of prerenal azotemia (transient increases in BUN > creatinine) as well as lethargy, piloerection, dyspnea, and gastrointestinal bleeding. Notably, death occurred in ~50% of animals at 48 mg/m^2 when carfilzomib was given as a bolus. Administration of the same dose (48 mg/m^2) as a 30 min infusion was well tolerated, with no changes in BUN and creatinine and substantially reduced signs of lethargy, piloerection, or dyspnea. Moreover, all animals in the infusion treatment groups survived. The only toxicity observed following infusion of carfilzomib over 30 min was gastrointestinal bleeding. The reduced toxicity seen with dosing by infusion may reflect the reduced C_{max} of carfilzomib vs that with bolus dosing. Inhibition of the pharmacological target of carfilzomib (the chymotrypsin-like activity of the proteasome) was equivalent in the bolus and 30 min infusion treatment groups.

In the clinic, the MTD of carfilzomib has not been reached in the multiple myeloma (MM) setting, particularly when administered as a 30-minute infusion. The 27mg/m2 of carfilzomib (bolus administration over 2-10') is well tolerated in MM patients overall and can be tolerated for >12 cycles in late stage MM patients with substantial comorbidities.

A phase 1 dose escalation study (PX-171-007) of single agent carfilzomib administered is ongoing and as of 10 July 2009, over 65 patients with solid tumors had started treatment in the initial Phase 2 portion of the study at 36 mg/m2 (bolus administration over 2-10'). A review of the tolerability of 36 mg/m2 carfilzomib in these patients indicates that this regimen was very well tolerated with only one DLT (fatigue) and an overall adverse event profile similar to that seen with the 27mg/m2 carfilzomib experience with bolus dosing (see IB for details). Three patients completed > 12 cycles of therapy at 36 mg/m2 with no evidence of cumulative toxicity. There were no significant DLTs observed; the majority of discontinuations on the study were due to progressive disease. Because of the long-term tolerability carfilzomib, the Phase 1b portion of this study was reopened, and a separate arm for multiple myeloma was added.

In the PX-171-007 trial, more recently patients have been treated with carfilzomib given as a 30-minute infusion in order to potentially minimize Cmax-related infusion events. The protocol was amended and doses of 20/36 (20 mg/m^2 given on Days 1 and 2 of cycle 1 only; followed by 36 mg/m^2 for all subsequent doses), 20/45, 20/56 and 20/70 mg/m^2 were investigated. Doses were given in two separate cohorts of patients with advanced MM and advanced solid tumors and lymphomas. The MTD in the solid tumor and lymphoma cohort was 20/70 mg/m^2. Further dose expansion was conducted at 20/56 mg/m^2 showing the regimen to be well tolerated. The MTD in the MM cohort was 20/56 mg/m^2. These data indicate that carfilzomib 30-minute infusion can be given at very high levels, with >95% inhibition of blood proteasome levels achievable and with (at least) acute tolerability. All protocols using ≥36mg/m^2 carfilzomib are now administering the drug as a 30-minute infusion.

In addition to the above observations, a phase I study of carfilzomib in patients with relapsed and refractory multiple myeloma was reported in abstract form at the 2009 American Society of Hematology meeting which demonstrated that carfilzomib can be safely administered to patients with substantial renal impairment (CrCl < 30, including patients on dialysis) without dose adjustment.[13] These data indicate that carfilzomib does not exacerbate underlying renal dysfunction, and confirm the "pre-renal" etiology of the BUN/creatinine elevations observed with IV bolus carfilzomib.

1.5 PEDIATRIC STUDY RATIONALE

1.5.1 PRECLINICAL STUDIES

Dr. Aru Narendran's laboratory has done extensive pre-clinical work on the effects of carfilzomib in pediatric leukemias and solid tumors, described below. [14,15]

Carfilzomib induces effective growth inhibition in cell lines derived from pediatric patients and primary leukemia specimens.

Increasing concentrations of carfilzomib were added to exponentially growing leukemic cell lines and the number of viable cells was quantified after four days in culture. Leukemia cell lines used were derived from infant and pediatric patients. Data presented in Fig. 1 shows that carfilzomib induced effective cell growth inhibition in all of the cell lines evaluated (IC50 values Table 1). These studies were also carried out in primary leukemia cells, including samples from infant leukemia. The effects of carfilzomib against JMML cells that proliferate in response to exogenous GM-CSF and continuous cultures of secondary AML cells that were derived from a patient who initially presented with ALL were also evaluated. Data shown in Fig. 2 provides evidence for carfilzomib activity towards primary leukemic cells in all of the patients studied.

Figure 1

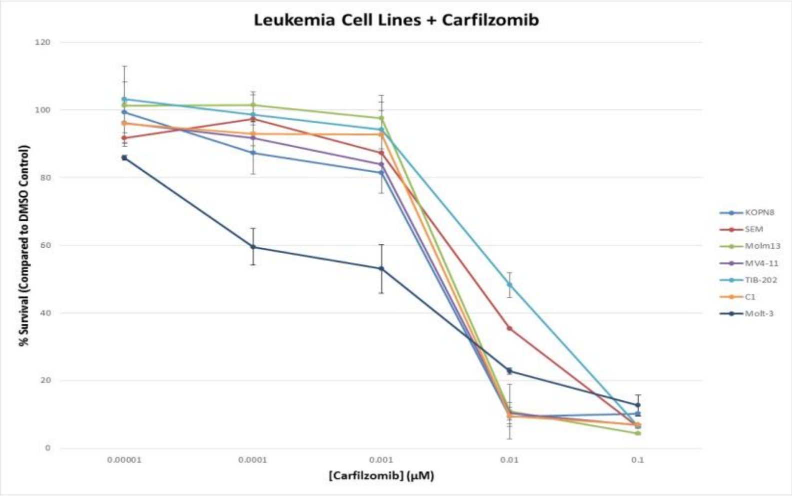

Table 1

Cell Line	Type	IC50 (μM)
C1	B-ALL	1×10^{-6}
SEM	B-ALL	0.008
Molt-3	T-ALL	0.001
Molm13	AML	0.008
MV-411	AML	5×10^{-6}
KOPN8	Infant ALL	0.0004
TIB-202	Infant AML	0.01

Figure 2

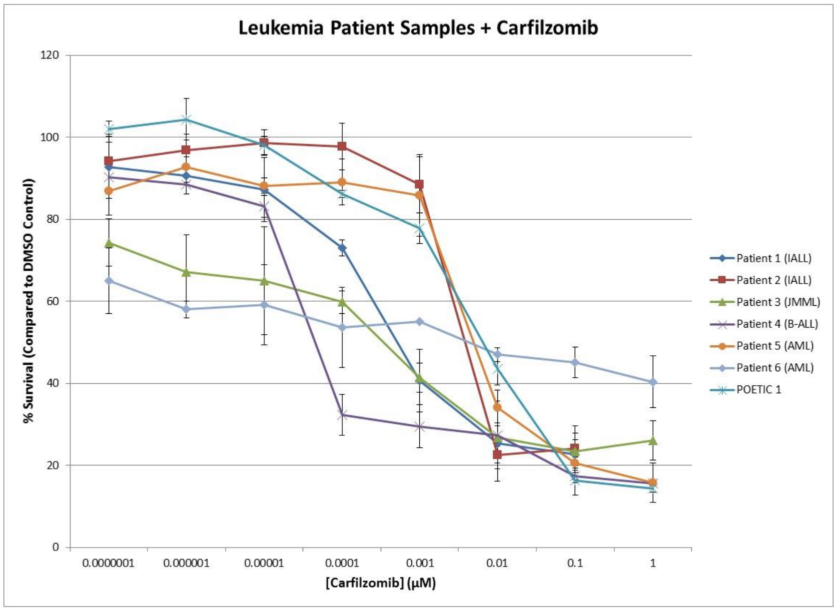

Table 2

Sample	Type	IC50 (µM)
Patient 1	Infant ALL	0.002
Patient 2	Infant ALL	0.01
Patient 3	JMML	0.0002
Patient 4	B-ALL	0.0001
Patient 5	AML	0.008
Patient 6	AML	0.006

In vitro synergy between carfilzomib and the conventional anti-leukemic agents.

Next, Dr. Narendran's lab evaluated the potential of carfilzomib in combination therapies in potential clinical studies. In these experiments, drug sensitivity assays were performed to evaluate the outcome of adding carfilzomib to cells that are exposed to cytarabine and etoposide. These assays used four representative cell lines: MOLM13, TIB202 (AML), KOPN8, and SEM (ALL). Combination indices are presented in Table 3. This information indicates that the addition of carfilzomib can produce synergistic or additive effect (SEM cells) when combined with either cytarabine or etoposide.

Table 3 Combination studies with carfilzomib in ALL cell lines

	Molm13			TIB-202		
Inhibitor	Alone (µM)	+ 0.001 µM Carfilzomib (µM)	Combination Index	Alone (µM)	+ 0.001 µM Carfilzomib (µM)	Combination Index
Cytarabine	0.01	0.008	0.9	8	0.1	0.2
Etoposide	0.1	0.06	0.7	8	4	0.6
Sorafenib	$< 1 \times 10^{-6}$	$< 1 \times 10^{-6}$	N/A	5	1	0.3
Mefloquine	7.7	2.2	0.4	7.2	0.6	0.2

	KOPN8			SEM		
Inhibitor	Alone (µM)	+ 0.001 µM Carfilzomib (µM)	Combination Index	Alone (µM)	+ 0.001 µM Carfilzomib (µM)	Combination Index
Cytarabine	0.003	0.002	0.8	0.02	0.02	1
Etoposide	0.07	0.02	0.5	0.8	0.4	0.7
Sorafenib	$< 1 \times 10^{-6}$	$< 1 \times 10^{-6}$	N/A	2	3	0.8
Mefloquine	7.3	3.8	0.7	8.7	4.8	0.7

In vitro preclinical studies involving cyclophosphamide are difficult to perform as the prodrug needs to be metabolized by the liver to produce active metabolites. However a number of previous studies have indicated the mechanistically based potential for synergy between cyclophosphamide and proteasome inhibitors.[16]

Carfilzomib activity leads to activation of apoptosis pathways.

Cytotoxicity studies show that carfilzomib treatment leads to a dose-dependent loss of viable cells in culture. To identify some of the potential mechanisms involved in this process Dr. Narendran's lab studied the effect of carfilzomib over a period of exposure times and evaluated the activation of three apoptosis-related proteins. Representative Western blot data presented in Figure 3 for an ALL and AML cell lines show that clearly identifiable changes in PARP cleavage and the generation of active fragments in caspase 7 and caspase 8 in time dependent and concentration dependent (Figure 4) manner. Similar findings were seen with other cell lines and primary cells with the exception of the KOPN8 cell line in which only caspase 8 activation was noted under the experimental conditions (data not shown). Following these observations Dr. Narendran's lab also investigated the effect of carfilzomib on the modulation of key pro-survival molecules in leukemia cells. Increasing concentrations of carfilzomib were added to leukemia cells and changes in cellular levels of BCL2 and MCL-1 were evaluated by Western blot analysis after 18 hrs. Figure 5 shows a concentration dependent up-regulation of MCL-1 where no notable changes in BCL2 were observed under identical conditions.

Figure 3. Induction of apoptosis in leukemia cell lines by carfilzomib

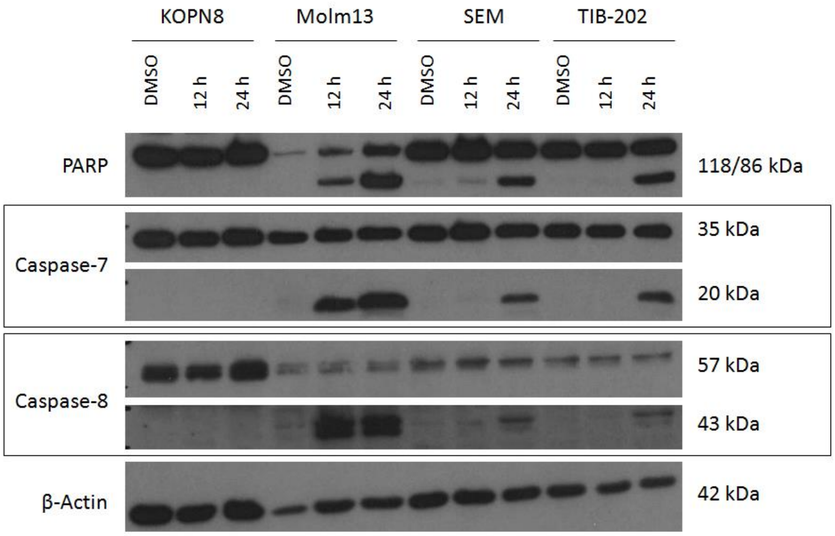

Cells treated with 0.01 µM (KOPN8) or 0.05 µM (Molm13, SEM, TIB-202) carfilzomib or vehicle control (DMSO) for 12 h and 24 h.

Figure 4

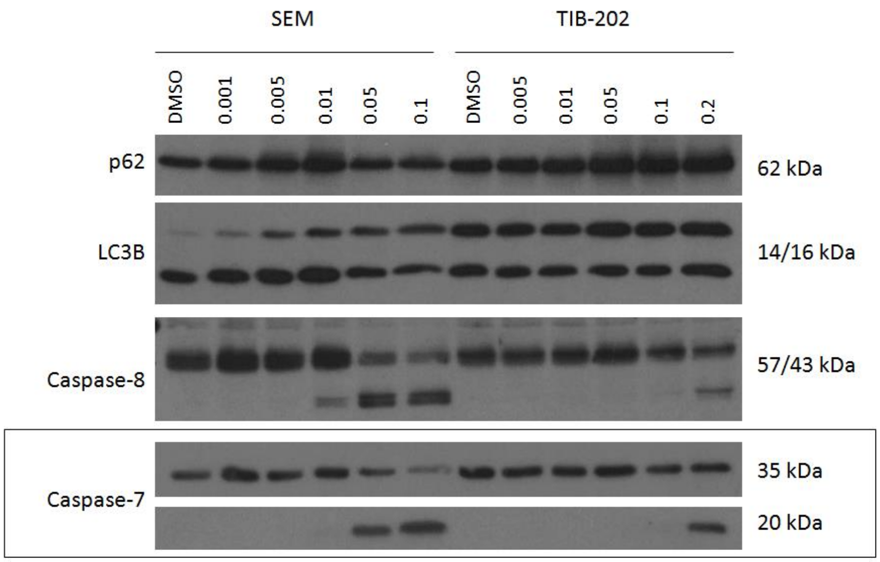

SEM and TIB-202 treated with increasing concentrations (0.001 – 0.2 µM) of carfilzomib or vehicle control (DMSO) for 12 h.

Figure 5. Increased apoptosis in cultures treated with combination of carfilzomib plus etoposide and cyclophosphamide

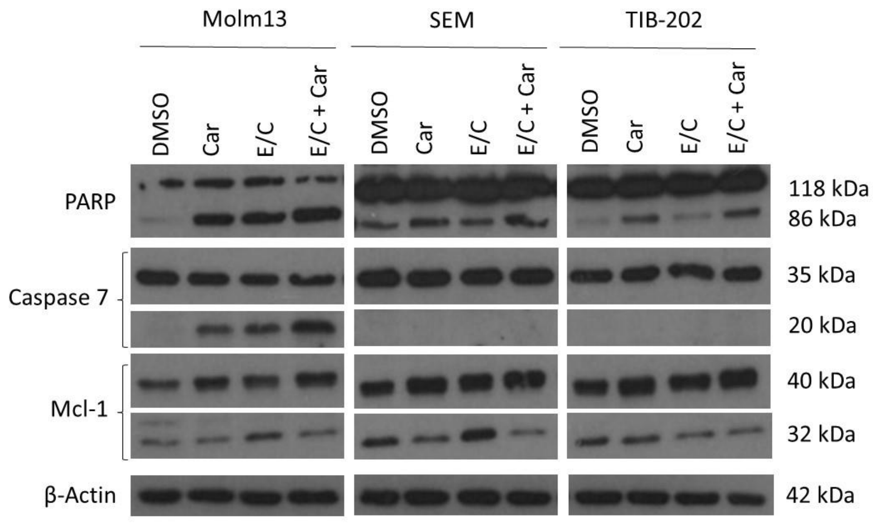

Cell lines treated with carfilzomib alone or in combination with etoposide and cyclophosphamide for 24 hours

Effective cytotoxicity of Carfilzomib in pediatric solid tumor cell lines in vitro.

Dr. Narendran's lab evaluated the ability of carfilzomib to induce cytotoxicity in a panel of pediatric tumor cells, including neuroblastoma (n=6), Ewings sarcoma (n=2), osteosarcoma (n=2), and ATRT (n=3). Exponentially growing tumor cells were treated with increasing concentrations of carfilzomib and after four days in culture, cell viability was measured. Significant growth inhibition was observed in a dose-dependent manner in all cell lines tested (Figure 6).

Figure 6. Activity of carfilzomib against pediatric solid tumor cell lines

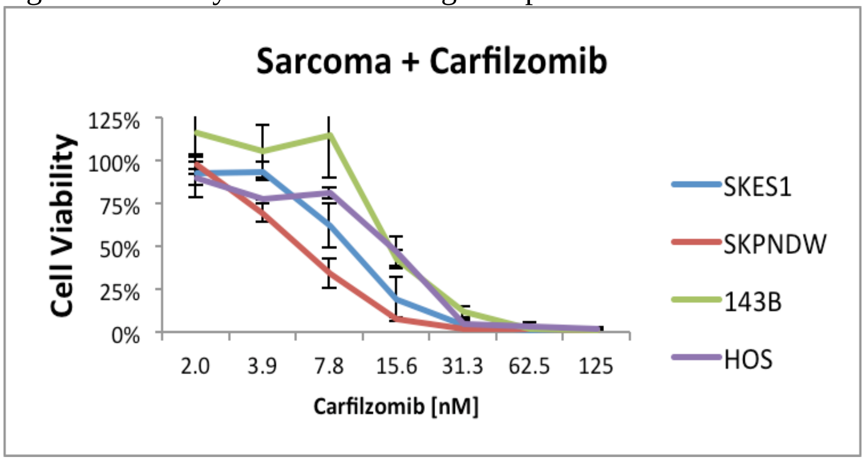

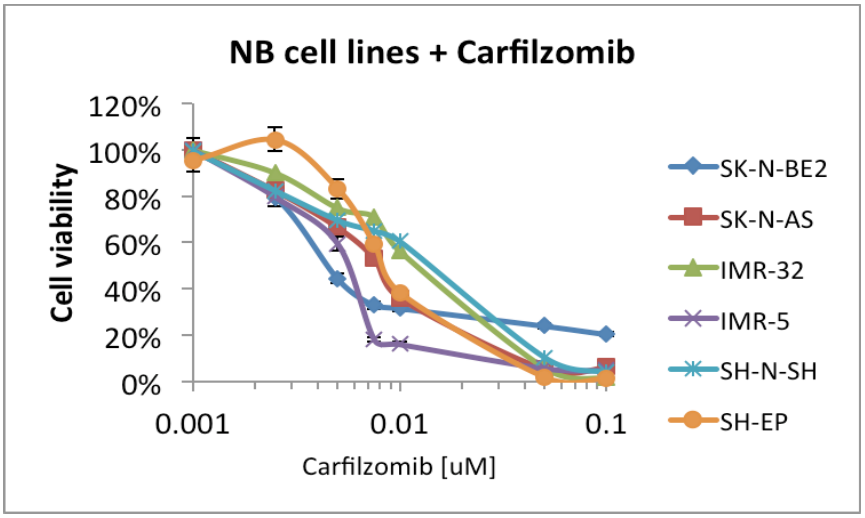

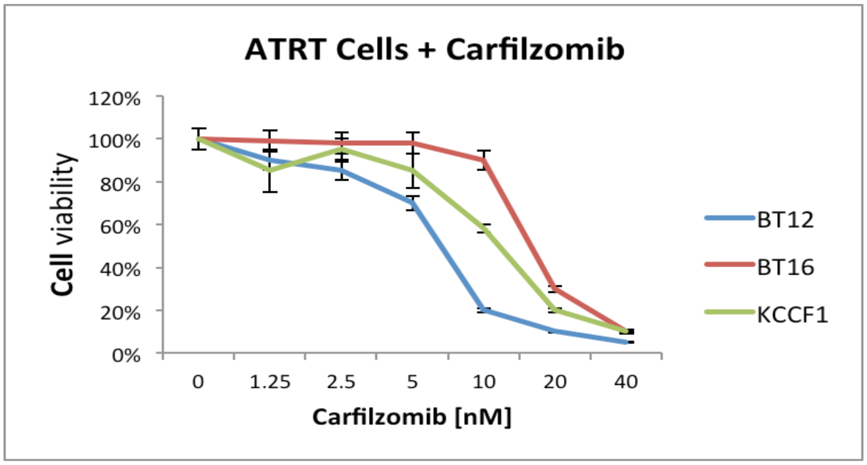

To confirm that carfilzomib exerts its activity via proteasome inhibition, neuroblastoma cell line IMR-5 was selected to measure the activity of 20S proteasome upon inhibition by carfilzomib or bortezomib using LLVY-AMC-based fluorescence detection assay. Both inhibitors displayed a dose-dependent inhibitory activity towards 20S proteasome (Figure 7). At 0.1 uM, 80% of the proteasome activity was blocked by carfilzomib.

Figure 7. Comparison of carfilzomib and bortezomib against the NB cell line IMR-5.

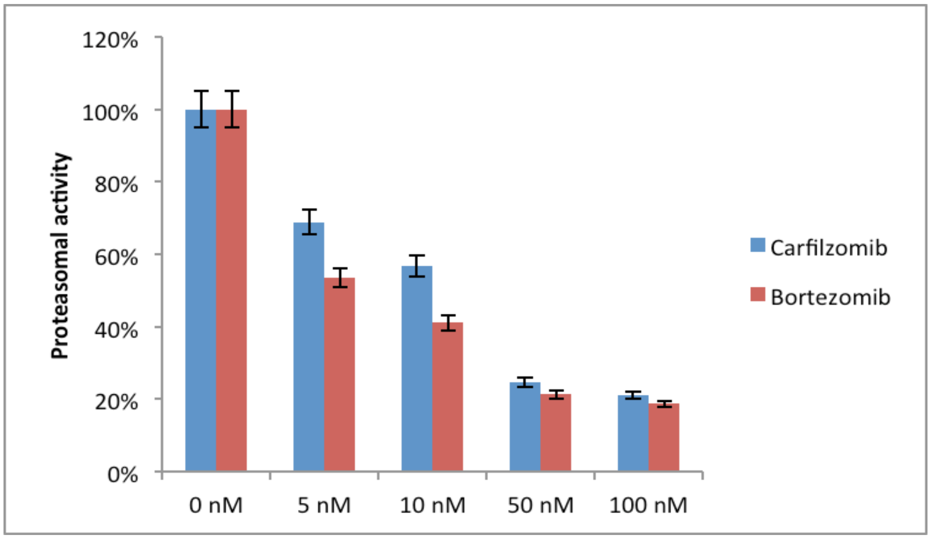

Effect of drug schedule of carfilzomib and chemotherapy on cytotoxic synergy.

To evaluate the scheduling effect of carfilzomib and chemotherapy in combination, two leukemia cell lines were treated with carfilzomib first followed by etoposide 12 hours later or etoposide followed 12 hours later by carfilzomib. Data obtained show that in the SEM cell line there was no difference in tumor cell survival based upon the order of administration However, for the MOLM13 cells, giving etoposide 12 hours prior to carfilzomib significantly increased the activity of the combination (Figure 8). This data supports the order drugs will be administered in this protocol.

Figure 8. Effect of drug administration order on synergy

Viable tumor cells

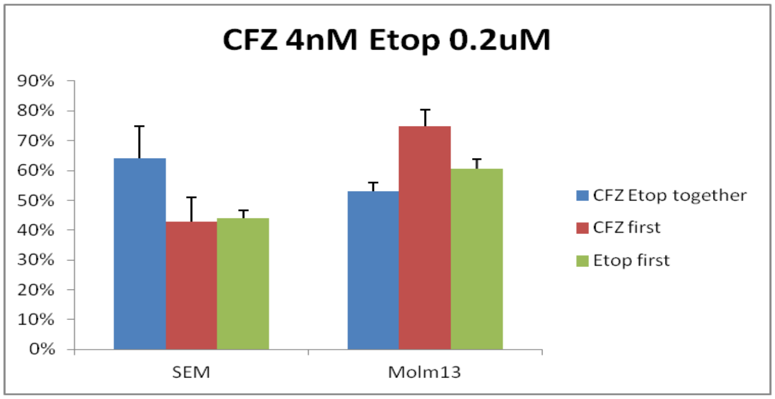

Carfilzomib first: Cells were exposed to carfilzomib for 12 hrs before the addition of etoposide.

Etop first: Cells were exposed to Etop for 12 hrs before the addition of carfilzomib.

1.5.2 Choice of Cyclophosphamide and Etoposide Backbone

Carfilzomib has not yet been evaluated in children. It will be a phase I combination study looking at carfilzomib in combination with a standard chemotherapy backbone for both solid tumors/lymphomas and leukemias. Solid tumors/lymphomas and leukemias will be analyzed and dose-escalated independently as separate cohorts.

The backbone will consist of cyclophosphamide and etoposide, both given daily for 5 days. The rationale for use of this backbone includes the following: 1) As described above, *in vitro* studies have shown synergy of carfilzomib in combination with etoposide and other studies have evaluated synergy between cyclophosphamide and proteasome inhibitors.[16] 2) Both cyclophosphamide and etoposide are active agents in pediatric leukemias [both lymphoid and myeloid] and solid tumors. 3) The combination has been used as a standard regimen to treat both solid tumors and leukemias. 4) Because of extensive experience with this regimen, the baseline toxicities are well known. This will be crucial in distinguishing toxicities of the backbone versus the carfilzomib. 5) There are multiple current adult combination trials with carfilzomib, some of which include cyclophosphamide or etoposide. 6) By the time of study enrollment, patients often will have received the maximum cumulative doses of anthracyclines, ruling out the use of anthracycline-containing treatment regimens. 7) Because of the rapid clearance *in vivo* in adult trials, it is thought that giving carfilzomib back-to-back with the standard chemotherapy will maximize synergy. Since the cyclophosphamide and etoposide regimen is given daily for 5 days, it can be given on the same days as the carfilzomib, utilizing the 5-day dosing schedule of the initial Phase I adult carfilzomib trial.[7]

1.5.3 Carfilzomib Pediatric Dosing Rationale

To best synergize with the cyclophosphamide and etoposide regimen, this study will start with the 5-day daily dosing schedule of carfilzomib that was trialed in PX-171-001.[7] As outlined above, the MTD on that trial of giving carfilzomib as a single agent was 15 mg/m^2. In that study, the carfilzomib was administered as an IV push over 1-2 minutes on days 1-5. In PX-171-007, it was shown that with the day 1,2,8,9,15,16 dosing regimen, the MTD could be doubled by increasing the infusion time to 30 min (for solid tumors/lymphomas, MTD increased from 36 mg/m2 to 70 mg/m2 and in multiple myeloma from 27 mg/m2 to 56 mg/m2).[17] Although this has not yet been trialed using the 5-day dosing regimen, it is thought that the MTD would likely be higher using the 30-minute infusion rate. Therefore, that is the rate that will be used for this study.

As 11 mg/m^2 was the lowest dose level that showed clinical activity in PX-171-001, that will be the initial starting dose for this study. For dose level 2, the dose will be increased to 15 mg/m^2, the Phase I single agent MTD with the rapid IV infusion rate and also a dose that showed >75% proteasome inhibition in whole blood and PBMCs 1 hour after dosing.[7] From there, the dose escalation will continue up to a maximum dose of 36 mg/m^2. There will be one dose level -1 de-escalation built in that maintains the carfilzomib at 11 mg/m^2, but decreases doses of the chemotherapy backbone by 25%.

In the unlikely event that that dose level -1 of the 5-day dosing regimen is not tolerated, then the protocol will change over to use the 2-day carfilzomib dosing schedule where the drug is given on days 1, 2, 8, 9, 15, 16. Although that would mean 6 doses in lieu of 5 doses per cycle, it is thought that if the 5 doses given daily for 5 days is not tolerated, it may be that 6 doses is tolerated if spread

out over 16 days. To be consistent with the adult studies and labeling, the plan is to include all 6 doses of carfilzomib per cycle.

If it is necessary to switch to the 2-day dosing schedule, the new dose escalation will start with 75% of the standard 5-day cyclophosphamide and etoposide dosing and the carfilzomib dose will be started at 27 mg/m^2 with a subsequent dose escalation to a maximum carfilzomib dose of 56 mg/m^2, the 30-minute infusion MTD for multiple myeloma patients as a single agent and the dose at which a dose expansion was performed for the solid tumor and lymphoma patients in PX-171-007.[17] There are also 2 built-in dose de-escalations that would first decrease the carfilzomib to 20 mg/m^2 and subsequently to 15 mg/m^2. If dose level -2 is not tolerated, then the regimen will be considered intolerable and the study will be completed.

A phenomenon noted in adult trials of carfilzomib is a "first dose effect," which is notable for fever, chills, rigors, and/or dyspnea following the first 1-2 doses of carfilzomib. Because of this, the initial FDA approval for the drug was for the lower dose of 20 mg/m^2 given for all doses in the first cycle, followed by an increase to 27 mg/m^2 for all subsequent cycles. Since then, it is now mandated that all patients receive premedication with dexamethasone prior to all doses in the first cycle. Following institution of that premedication requirement, the incidence of the first dose effect has decreased significantly. Currently, most trials with carfilzomib only administer 20 mg/m^2 for the first 2 doses of the first cycle before moving up to the higher dose for all other doses in the first cycle as well as all doses in subsequent cycles. This has been well tolerated.

In January 2015, the trial of carfilzomib, lenalidomide and dexamethasone for relapsed multiple myeloma was published in the New England Journal of Medicine.[18] The trial results were submitted to the FDA by Onyx as the complete analysis of that trial was a condition of the accelerated approval of carfilzomib. In that trial, only doses 1 and 2 of the first cycle were given as 20 mg/m^2 prior to the dose escalation to 27 mg/m^2 thereafter. This was well tolerated without complications. As a result of this trial, on January 29, 2015, Onyx submitted an application to the FDA to change the labeling to only require the 20 mg/m^2 on days 1 and 2 before stepping up the dose.

This trial will base the step-up dosing schedule on this most recent data, the current use of this dosing in most current trials, and the high likelihood that this will become the standard adult dosing. In this trial, patients assigned to dose levels higher than 20 mg/m^2 will be given 20 mg/m^2 for the first 2 doses of cycle 1 only. The remainder of doses will be given as assigned for that dose level.

2 OBJECTIVES

2.1 PRIMARY OBJECTIVE

To determine the DLT and MTD of carfilzomib given in combination with cyclophosphamide and etoposide in pediatric patients with relapsed/refractory leukemias and solid tumors/lymphomas.

2.2 SECONDARY OBJECTIVES

1. To evaluate toxicities of carfilzomib in the pediatric population when combined with conventional chemotherapy
2. To gather preliminary efficacy data on the drug combination
3. To measure if circulating plasma proteasome (cProt) levels post treatment correlate with response to therapy and overall survival
4. To measure if the levels of proteasome activity and resistance to carfilzomib correlates with toxicity and/or response to treatment
5. To measure if inhibition of proteasome activity by carfilzomib results in alteration in a number of autophagy and apoptosis related proteins, providing means to evaluate correlates of activity of carfilzomib
6. To measure the level of proteasome inhibition in patient PBMCs before and during treatment by determination of the level of protein ubiquitination.
7. To determine *in vitro* sensitivity of patient leukemias and solid tumors to carfilzomib alone and in combination with study chemotherapeutic agents in order to generate a predictive model of drug sensitivity.
8. To perform whole exome sequencing (WES) and RNA seq on patient leukemia and solid tumor samples and WES on germ line DNA in order to determine potential mechanisms of drug sensitivity or resistance.

2.3 EXPLORATORY OBJECTIVES

1. To gather information on the methylation pattern of DNA and other epigenetic markers before and during treatment.
2. To evaluate potential biomarkers for measuring response and/or resistance to carfilzomib treatment.

3 EXPERIMENTAL PLAN

3.1 STUDY DESIGN

3.1.1 DISEASE STRATA

Patients will be enrolled into Strata A or B, depending upon their underlying disease.

- Strata A – leukemia patients
- Strata B – solid tumor (including lymphoma) patients. If a lymphoma patient has >=25% bone marrow blasts, then the patient will be considered a leukemia patient and will be enrolled in strata A. The dose escalations of each strata will be performed independently

3.1.2 Treatment and Dose Escalation

Dose escalation will follow the rolling-six design (see section 10.1 for details)

Doses will be escalated based upon DLTs (see section 6.2 for DLT definitions) until the MTD or the highest dose level is reached, whichever comes first.

At the MTD or the highest dose level (if no MTD is reached), an additional 6 patients will be enrolled to further evaluate safety of the regimen.

The initial dose escalation will utilize a 5-day dosing schedule of carfilzomib as described below.

Dose Level	Cyclophosphamide (mg/m^2)	Etoposide (mg/m^2)	Carfilzomib (mg/m^2)
-2	See twice weekly dosing schedule (see below)		
-1	330	75	11
1	440	100	11
2	440	100	15
3	440	100	20
4	440	100	20/27*
5	440	100	20/36*

*For the FIRST CYCLE ONLY, each patient at dose levels 4 and 5 will receive 20 mg/m^2 on days 1 and 2 prior to escalation to 27mg/m^2 or 36 mg/m^2 as above for days 3-5. For all subsequent cycles, the patient will receive 27 mg/m2 or 36 mg/m2 as above, on all days 1-5.

All 3 drugs will be administered daily on days 1-5 schedule in the following order:

- Hour 0-1 Cyclophosphamide IV for 60 minutes (± 5 minutes)
- Hour 1-3 Etoposide IV for 120 minutes (± 10 minutes)
- Hour 3-3.5 Carfilzomib IV for 30 minutes (± 5 minutes)

Acute leukemia/ Non-Hodgkin's lymphoma (NHL) patients will receive a single dose of IT chemotherapy within 14 days of starting systemic therapy. It is permitted for this to have been administered prior to study enrollment. If an IT dose was given within the 14-day period, then a second dose should NOT be given prior to start of systemic chemotherapy. All IT should be based upon standard dosing for age:

ALL/NHL patients should receive IT methotrexate

- 0.5-0.99 yo - 6 mg
- 1-1.99 yo – 8 mg
- 2-2.99 yo – 10 mg
- 3-8.99 yo – 12 mg
- ≥9 yo – 15 mg

AML and MPAL patients should receive IT cytarabine

- 0.5-0.99 yo – 20 mg
- 1-1.99 yo – 30 mg
- 2-2.99 yo – 50 mg
- >= 3 yo – 70 mg

IF DOSE DE-ESCALATION IS REQUIRED BELOW DOSE LEVEL -1 OF THE 5-DAY DOSING SCHEDULE ABOVE (i.e. Dose level -1 is determined to be above the MTD), THEN THE STUDY WILL MODIFY THE DOSE ESCALATION TO THE 2-DAY DOSING SCHEDULE OF CARFILZOMIB AS SHOWN BELOW.

Dose escalation of the 2-day schedule will begin at dose level 1.

Dose Level	Cyclophosphamide (mg/m^2)	Etoposide (mg/m^2)	Carfilzomib (mg/m^2)
-2	330	75	15
-1	330	75	20
1	330	75	20/27*
2	440	100	20/27*
3	440	100	20/36*
4	440	100	20/45*
5	440	100	20/56*

*For the FIRST CYCLE ONLY, each patient at dose levels 1 to 5 will receive 20 mg/m^2 on days 1 and 2 prior to escalation to 27mg/m^2, 36 mg/m^2, 45 mg/m^2, or 56 mg/m^2 as above for days 8,9,15,and 16. For all subsequent cycles, the patient will receive 27 mg/m^2, 36 mg/m^2, 45 mg/m^2, or 56 mg/m^2 as above on all days 1,2,8,9,15, and16.

Cyclophosphamide and Etoposide with be administered daily on days 1-5, and Carfilzomib will be administered on days 1, 2,8,9,15,16.

Cyclophosphamide and Etoposide daily on Days 1-5:

- Hour 0-1 Cyclophosphamide IV for 60 minutes (± 5 minutes)
- Hour 1-3 Etoposide IV for 120 minutes for (± 10 minutes)

Carfilzomib on Days 1, 2,8,9,15,16

- Days 1, 2 – Carfilzomib given from Hour 3-3.5 IV for 30minutes (± 5 minutes)
- Days 8, 9, 15, 16 - Carfilzomib given alone over 30 min (±5 minutes)

Acute leukemia/NHL patients will receive IT as above.

Patients with a BSA> 2.2 m^2 will be dosed for Etoposide, Cyclophosphamide, and Carfilzomib based upon a BSA of 2.2 m^2.

Patients <10 kg should be dosed for Etoposide, Cyclophosphamide, and Carfilzomib based upon weight in lieu of BSA. The dose per kg used will be the dose per m2 divided by 30. (For example, if a patient is 7 kg at dose level 1, the carfilzomib dose should be 27/30 mg per kg [0.9 mg/kg] x 7 kg = 6.3 mg)

All patients on either dosing schedule, will start granulocyte colony-stimulating factor (G-CSF) on Day 6 of each cycle, between 24-36 hours after completion of carfilzomib administration. This can be in the form of:

- Filgrastim (Neupogen®) 5 mcg/kg subcutaneously daily until *at least* post-nadir ANC >1000. G-CSF must be discontinued at least 48 hours prior to beginning the next cycle. The patient must meet blood count criteria for the next cycle once ≥ 48 hours off the G-CSF.

- For leukemia patients, it is recommended that the patient be off G-CSF for at least 48 hours (preferably longer) before a bone marrow assessment is performed as it can confound the disease status assessment, particularly in the case of AML. If a leukemia patient has an M2 or M3 marrow which is thought possibly related to G-CSF effect, then further treatment should be delayed and the bone marrow repeated one week later to assess disease response.

OR

- Pegfilgrastim (Neulasta®) 0.1 mg/kg (max 6 mg) subcutaneously x 1 dose

The choice will be up to the individual investigator. However, please note that Pegfilgrastim (Neulasta®) use is not recommended for children under 40 kg.

For their first cycle, patients will be pre-medicated with dexamethasone 0.1 mg/kg (max 4 mg) on all days that they are receiving carfilzomib. See section 6.1 regarding mandatory pre-medication and hydration with chemotherapy administration.

3.1.3 Chemotherapy Cycles

Each cycle will be at least 28-days long. The patient must receive a minimum of one cycle. If a patient shows response to therapy, has recovered from the toxicities of therapy, and meets the same

laboratory and organ function inclusion and exclusion criteria as used for initial enrollment (ECHO/EKG need not to be repeated unless clinically indicated), the patient will be permitted to receive an additional cycle. The next cycle will begin on Day 29-31 of the previous cycle. This can be delayed for recovery from toxicity, while awaiting meeting criteria to start, or if a leukemia patient requires repeat bone marrow assessments because bone marrow is too hypocellular to make an adequate disease assessment or because of possible G-CSF effect confounding interpretation (see above).

There is no maximum number of cycles a patient can receive. The number of cycles beyond one will be at the discretion of the investigator as long as the above criteria continue to be met.

If a patient experiences a DLT and is fully recovered and has shown a response to therapy, then a single dose de-escalation will be permitted for subsequent cycles. If the patient has a subsequent AE that would be considered a DLT, then the patient will be taken off protocol.

There will be no intra-patient dose escalation.

3.1.4 Disease Assessment

For leukemia patients, disease assessments will be performed after every cycle.

For solid tumor/lymphoma patients, disease assessment will be performed after cycles 2, 4, 6, and then a minimum of every 3 cycles. Assessments must be completed prior to starting the subsequent cycle. If a patient shows signs of disease progression, then disease assessment should be performed at that time.

See Section 10.12 for more details about disease evaluation.

3.2 NUMBER OF CENTERS

8

3.3 NUMBER OF SUBJECTS

DEPENDENT UPON DOSE ESCALATION. ESTIMATE-50

3.4 ESTIMATED STUDY DURATION

Approximately 8 years for patient accrual and follow-up.

4 SUBJECT SELECTION

4.1 INCLUSION CRITERIA

Subjects must meet all of the following inclusion criteria to be eligible to enroll in this study.

Disease-related:

1. Patients must have either of the following:
   a. Relapsed/refractory leukemia in 2nd or greater relapse or who have failed at least one re-induction attempt after relapse or for refractory disease. Patients must meet the WHO classification with ≥ 5% blasts in the bone marrow or must have definitive extramedullary disease (e.g. chloromas, skin lesions). Patients may have asymptomatic CNS 1 or CNS 2 disease, but not CNS 3 or symptomatic CNS disease. (See section 4.3 for CNS status definitions)

   OR

   b. Relapsed/refractory non-CNS solid tumor that has not responded or has relapsed and for which no standard treatment is available. Patients may not have primary CNS tumors or CNS metastases. Lymphoma patients are permitted. Patients do not need to have measurable disease.

Demographic:

2. Age 6 months-29.99 years at enrollment
3. Life expectancy ≥ 3 months
4. Lansky or Karnofsky ≥50
5. Prior therapy
   a. Patient must have fully recovered from the acute toxic effects of all prior chemotherapy, immunotherapy, radiotherapy, or surgery prior to study entry.
   b. Myelosuppressive therapy- At least 14 days must have elapsed since the administration of previous therapy. Six weeks must have elapsed from the administration of nitrosoureas or mitomycin C. For patients with ALL on maintenance therapy, they may be eligible if 7 days have elapsed and they are recovered from the toxic effects of the chemotherapy. This restriction does not include intrathecal chemotherapy, which is permitted. Hydroxyurea is permitted but must be discontinued ≥24 hours prior to start of protocol therapy.
   c. Biologic agents- At least 14 days must have elapsed since the completion of therapy with a biologic agent such as a monoclonal antibody. Seven days must have elapsed since the last dose of retinoids

   d. Radiation therapy – At least 14 days must have elapsed for local XRT. At least 90 days must have elapsed if prior radiation to ≥50% of the pelvis, the spine, or other substantial bone marrow radiation including TBI.
   e. Hematopoietic growth factors- At least 7 days must have elapsed since the last dose of G-CSF or GM-CSF. At least 14 days must have elapsed since last dose of pegfilgrastim (Neulasta®).

6. Patient must be ≥ 3 months from hematopoietic stem cell transplant, must not have active GVHD, and must be off all immunosuppression

Laboratory

7. Organ function:
   a. Either a serum creatinine ≤ ULN for age, or calculated or measured GFR ≥ 70 mL/min/1.73 m^2
   b. Total bilirubin ≤ 1.5 x ULN for age, direct bilirubin ≤ ULN for age
   c. AST and ALT ≤ 3 x ULN for age unless elevation can be clearly attributed to liver leukemia or metastases
   d. ECHO shortening fraction ≥ 27%
   e. Pulse oximetry measurement ≥ 95% saturation without supplemental oxygen

8. Bone marrow function
   a. Hgb ≥10 g/dL – can be transfused
   b. Plts ≥ 75,000 – cannot be transfused (must be ≥ 7 days from last plt transfusion)
   c. ANC ≥ 750 – cannot be transfused (must be ≥ 72 hours from last neutrophil infusion)

   However, the plt and ANC requirements can be waived if low counts thought to be secondary to leukemia or tumor bone marrow infiltration.

Ethical/Other

9. Reproductive function:
   a. Female patients of childbearing potential must have a negative serum pregnancy test confirmed within 7 days prior to enrollment
   b. Female patients with infants must agree not to breastfeed their infants while on the study
   c. Male and female patients of child-bearing potential must agree to use an effective method of contraception approved by the investigator during the study and for a minimum of 3 months after study treatment

10. Written informed consent

4.2 EXCLUSION CRITERIA

Concurrent Conditions

1. Prior treatment with carfilzomib
2. Known allergy to Captisol® (a cyclodextrin derivative used to solubilize carfilzomib).
3. Down syndrome
4. Fanconi Anemia or other underlying bone marrow failure syndrome
5. Pregnant or lactating females
6. Known history of Hepatitis B or C or HIV
7. Patient with any significant concurrent illness
8. Patient with uncontrolled systemic fungal, bacterial, viral or other infection with ongoing signs/symptoms despite appropriate treatment
9. Patient with illness, psychiatric disorder or social issue that could compromise patient safety or compliance with the protocol treatment or procedures, interfere with the consent, study participation, follow-up, or interpretation of study results.

4.3 CNS Status Definitions

Only CNS1 and CNS2 are eligible, and only in the **absence** of neurologic symptoms suggestive of CNS leukemia, such as cranial nerve palsy. CNS status is defined as:

- CNS 1: In cerebral spinal fluid (CSF), absence of blasts on cytospin preparation, regardless of the number of white blood cells (WBCs).
- CNS 2: In CSF, presence < 5/μL WBCs and cytospin positive for blasts, or > 5/μL WBCs but negative by Steinherz/Bleyer algorithm:
    - CNS 2a: < 10/μL RBCs; < 5/μL WBCs and cytospin positive for blasts;
    - CNS 2b: ≥ 10/μL RBCs; < 5/μL WBCs and cytospin positive for blasts; and
    - CNS 2c: ≥ 10/μL RBCs; ≥ 5/μL WBCs and cytospin positive for blasts but negative by Steinherz/Bleyer algorithm (see below).
- CNS3: In CSF, presence of ≥ 5/μL WBCs and cytospin positive for blasts (in the absence of a traumatic lumbar puncture) and/or clinical signs of CNS leukemia.

STEINHERZ/BLEYER ALGORITHM FOR EVALUATING TRAUMATIC LUMBAR PUNCTURES:

If the patient has leukemic cells in the blood and the lumbar puncture is traumatic and the cytospin contains ≥ 5 WBC/µL with blasts, the following algorithm should be used to distinguish between CNS2 and CNS3 disease:

$$\frac{\text{CSF WBC}}{\text{CSF RBC}} > 2\text{X} \quad \frac{\text{Blood}}{\text{Blood RBC}}$$

Therefore, a patient with CSF WBC ≥ 5/µL blasts, whose CSF WBC/RBC is 2X greater than the blood WBC/RBC ratio, has CNS disease at diagnosis.

For example, the following patient would be classified as CNS3: CSF WBC = 60/µL; CSF RBC = 1500/µL; blood WBC = 46000/µL; blood RBC = 3.0 X 10^6/µL:

$$\frac{60}{0.015\ 1500} = 0.04 > 2\text{X} \quad \frac{46000}{3.0 \times 10^6} =$$

5 SUBJECT ENROLLMENT

5.1 RESEARCH PARTICIPANT REGISTRATION

Confirm eligibility as defined in the section entitled Inclusion/Exclusion Criteria. Obtain informed consent, by following procedures defined in section entitled Informed Consent Procedures. During the registration process registering individuals will be required to complete a protocol specific Eligibility Checklist. The individual signing the Eligibility Checklist is confirming that the participant is eligible to enroll in the study. Study staff are responsible for ensuring that all institutional requirements necessary to enroll a participant to the study have been completed.

Registration for this study will take place at the POETIC Data Coordinating Center (DCC) located within Pediatric Hematology/Oncology, Stanford University. All research participants will be registered through the REDCap system by the POETIC DCC staff.

All potential participants must be discussed and approved by the Study Principal Investigator and/or the POETIC Director or designee prior to being granted a slot reservation.

The participating site must contact the POETIC DCC to confirm slot availability and reserve a slot on the protocol when a patient is being considered for the trial.
The following documents must be submitted and completed by the registering site for each enrollment within 48 hours of the informed consent form being signed. The POETIC DCC telephone number is 650-736-0269 and the fax number is 650-736-3731. The POETIC DCC email address is poeticdcc@stanford.edu.

- The de-identified signed informed consent and HIPAA Authorization form
- The completed eligibility checklist provided by the POETIC DCC
- Supporting source documentation for eligibility questions (e.g. laboratory results, pathology report, radiology reports, MD notes, physical exam sheets, medical history, prior treatment records, and EKG report).

Upon receipt, the research staff at the POETIC DCC will conduct an interim review of all documents and confirm eligibility. If the eligibility checklist is not complete or source documentation is missing, the participant registration will be held until the completed documents are provided. The site will be responsible for sending the completed registration documents within 30 days of the consent prior to protocol treatment or any research tests.

Registrations will occur between 8:00 AM and 4:00 PM Pacific Standard Time (PST), Monday through Friday. Registration documents must be sent directly to the DCC prior to 3:00 PM PST to ensure timely processing and adequate time for source verification. If a registration is sent to the DCC after 3:00PM PST, all efforts will be made to have the patient registered that day; however, same day registration will not be guaranteed.

Once eligibility is confirmed, the participant is registered and will be assigned a protocol participant number by the POETIC DCC. This number is unique to the participant and must be written on all data and correspondence related to the participant. This protocol participant number will be communicated to study staff at the registering site via e-mail and will serve as the enrollment confirmation.

Regulatory documents for each participating site must be current and the site must be in good standing before registrations are completed.

6 TREATMENT PROCEDURES

Study treatment must begin within 72 hours of study enrollment. Certain exceptions, such as for intervening holidays, may be made after discussion with the Study Principal Investigator. Study tests and observations must be obtained within the windows specified in section 7.

6.1 DRUG PREPARATION AND ADMINISTRATION

- **Cyclophosphamide** should be prepared as per standard institutional protocol (see section 12.1). Cyclophosphamide will be administered by IV infusion over 60 minutes (± 5 minutes) from hour 0 – 1.
- **Etoposide** should be prepared as per standard institutional protocol (see section 12.2). Etoposide will be administered by IV infusion over 120 minutes (± 10 minutes) from hour 1 – 3.
- **Carfilzomib** for Injection is supplied as a lyophilized parenteral product in single-use vials. The lyophilized product is reconstituted with 29 mL Sterile Water for Injection, USP, to a final carfilzomib concentration of 2mg/mL prior to further dilution (see section 11.2 for detailed dilution instructions). Carfilzomib will be given as an IV infusion over 30 minutes (± 5 minutes) from hour 3-3.5. The dose will be administered at a facility capable of managing hypersensitivity reactions. Do not administer with other medicinal products.
- **IV lines** are to be flushed per facility standards between each medication.
- **IV pre-hydration** must be given daily immediately prior to hour 0 cyclophosphamide start. It is recommended that patients receive a minimum of D5 0.45% normal saline or D5 0.9% normal saline, normal saline or comparable IVF at a rate of 125 mL/m^2/hour for 2 hours prior to starting cyclophosphamide. The cyclophosphamide at hour 0-1 will be immediately followed by etoposide at hours 1-3 to be immediately followed by carfilzomib at hour 3-3.5. The initial IV hydration, cyclophosphamide and etoposide administration will serve as adequate pre-hydration for the carfilzomib dose. If the carfilzomib is dose de-escalated from the 5-day to the 2-day dosing schedule, then on days when the patient is only receiving carfilzomib, the patient should be pre-hydrated with the 125 mL/m^2/hour for 2 hours as above prior to the carfilzomib dose. Following completion of the carfilzomib dose, the patient should receive an additional two hours of **post-hydration** of 125 mL/m^2/hour. This will also serve as post-hydration for the cyclophosphamide (in addition to the subsequent etoposide and carfilzomib doses). Patients with active leukemia or non-Hodgkin's lymphoma (NHL) not in CR should receive continuous IV hydration at a minimum throughout the 5 days of cyclophosphamide and etoposide treatment along with appropriate tumor lysis syndrome prophylaxis and monitoring (see section 6.4.1). All subjects should be monitored closely for evidence of fluid overload
- It will be mandated that patients stay inpatient during the first 5 days of Cycle 1, then as medically necessary for good clinical care. For all subsequent cycles, the patients may be treated on an outpatient basis. Subjects will remain at the clinic under observation for at least 1 hour following the 1st dose of Cycle 2. During this observation times, post dose IV hydration will be given as outlined above.

6.2 DEFINITION OF DOSE-LIMITING TOXICITY (DLT)

Subjects will be evaluated for toxicity according to the Common Terminology Criteria for Adverse Events (CTCAE) of the National Cancer Institute (NCI) version 4.03 (Appendix A). A DLT must

be reported to the POETIC Data and Coordinating Center at Stanford University and to the study Principal Investigator.

The definition of DLT will be based upon the extensive experience with known toxicities of the cyclophosphamide and etoposide chemotherapy backbone being used in this study. The goal is to identify toxicities either specific to or worsened by the study drug, carfilzomib. Only those toxicities occurring in cycle 1 will be considered DLTs for the purposes of dose escalation and de-escalation. Toxicities that occur in subsequent cycles will be handled through dose modifications (Section 6.3) but will not figure into the definition of MTD.

A DLT is defined as any of the following toxicities that are *possibly*, *probably*, or *definitely* attributable specifically to carfilzomib:

Non-hematologic toxicities:

Any Grade 4 toxicity

OR

Any Grade 3 toxicity without resolution to ≤ Grade 2 or baseline (with optimal supportive care) within 7 days of the toxicity onset

Except the following **specific exclusions:**

- Alopecia
- Anorexia
- Correctable metabolic/electrolyte abnormalities. This includes manifestations of tumor lysis syndrome and associated precautions (e.g. intentionally not correcting hypokalemia)
- Fever with or without neutropenia
- Elevation of transaminases, alkaline phosphatase, total or fractionated (direct and/or indirect) bilirubin that return to ≤ Grade 2 elevation within 14 days of the toxicity onset
- Grade 3 infection

Any grade of toxicity that results in delay of starting Cycle 2 by more than 2 weeks will be considered a DLT.

Hematologic toxicities:

Patient will be considered to have a DLT if the ANC does not recover to ≥ 750 or the platelets to ≥ 75k by Day 43 of cycle 1. The cytopenia must be due to bone marrow aplasia/hypocellularity and not leukemic/malignancy infiltration.

No other hematologic toxicities will be considered DLTs.

6.3 DOSE REDUCTIONS/ADJUSTMENTS

If a patient experiences a toxicity that meets the definition of DLT during any treatment cycle, the treatment in the subsequent cycles should be reduced by one dose level. If the patient has a subsequent toxicity meeting the DLT definition, then the patient will be removed from study. However, if the patient experiences the DLT at dose level -1 of the 5-day carfilzomib dosing regimen, no further intra-patient dose de-escalation will be allowed. In other words, the patient will not be permitted to change from the 5-day to the 2-day dosing regimen.

The exception to the above is if a toxicity is definitely attributable to one of the non-study chemotherapies and not the carfilzomib. In that case, that specific chemotherapy drug only should be adjusted and/or standard supportive care (such as the addition of mesna or increased hydration) given. Any adjustments must first be discussed with the study Principal Investigator. However, if the toxicity occurs again, the patient will be removed from study.

For patients with allergic reaction to etoposide, etoposide phosphate at the same dose may be substituted.

The following are specific dose reductions for renal and liver and neuro toxicity. These should be applied for any toxicities occurring within a treatment cycle. While any of these toxicities are present, the patient will be ineligible to start the next treatment cycle.

Dose Adjustment Guidelines for Toxicity:

Toxicities		Cyclophosphamide	Etoposide	Carfilzomib
**Renal Dysfunction**	CrCl 15-50 mL/min/1.73m2<br>CrCl 10-15 mL/min/1.73 m2<br>CrCl < 10 mL/min/1.73 m2	Full dose<br>Full dose<br>Reduce dose by 50%	Reduce dose by 25%<br>Reduce dose by 25%<br>Reduce dose by 50%	Full dose<br>Hold until CrCl > 30 mL/min/1.73 m2 then restart at one level dose reduction
**Liver dysfunction**	Direct bilirubin > 2 mg/dL	Full dose	Reduce dose by 50%	Full dose
	Direct bilirubin ≥3 and < 5 mg/dL	Full dose	Reduce dose by 75%	Full dose
	Direct bilirubin ≥5	Full dose	Hold	Full dose
**Neuropathy***	Grade 3 that persists more than 7 days	Full dose	Full dose	Hold until resolves to ≤ Grade 2 or baseline, then restart at one dose level reduction
	Grade 4	Full dose	Full dose	Discontinue
**PRES^**	Any Grade	Full dose	Full dose	Discontinue

*If a patient develops central neuropathy possibly, probably, or definitely related to intrathecal chemotherapy, decisions regarding future IT treatment including drug choice will be at the discretion of the local investigator. This should be discussed with the study Principal Investigator.

^Posterior Reversible Encephalopathy Syndrome

6.3.1 Missed Doses

Missed doses will not be replaced during a cycle. The patient will be considered evaluable for disease response during cycle 1 only if the patient receives all required doses of carfilzomib, cyclophosphamide, and etoposide. If a dose needs to be held for any medical reason possibly, probably, or definitely related to carfilzomib administration during cycle 1, that will be considered a

DLT. If the medical reason is unlikely or definitely not related to the carfilzomib, then the patient will be considered inevaluable and will be replaced by another patient in the dose escalation.

6.3.2 Changes In Body Surface Area (BSA) And Weight

BSA will be recalculated at the beginning of each cycle and medications dosed accordingly. For patients <10 kg, weight at the beginning of each cycle will be used for dose calculations.

6.4 SAFETY CONSIDERATIONS

Based upon the experience in the Phase 1 and 2 clinical studies with carfilzomib, the following observations are noted:

- A "first dose effect" has been seen, which is notable for fever, chills, rigors, and/or dyspnea occurring during the evening following the first day of infusion and an increase in creatinine on Day 2, which may be the clinical sequelae of rapid tumor lysis and/or cytokine release.
- Should a "first dose effect" occur at any point, treatment with high dose glucocorticoids (e.g. methylprednisolone 1-2 mg/kg) is recommended. In addition, intravenous fluids, vasopressors, oxygen, bronchodilators, and acetaminophen should be available for immediate use and instituted, as medically indicated.
- Dexamethasone 0.1 mg/kg (max 4 mg) PO/IV will be administered prior to all carfilzomib doses during the 1st cycle. On days when patients are receiving cyclophosphamide and etoposide prior to carfilzomib, the dexamethasone should be administered along with other premedications prior to starting cyclophosphamide at hour 0.
- If treatment-related fever, rigors, chills, and/or dyspnea are observed post any dose of carfilzomib after dexamethasone has been discontinued for subsequent cycles, dexamethasone 0.1 mg/kg (max 4 mg) PO/IV should be re-started and administered prior to all subsequent doses.
- CrCl changes are mostly transient, reversible, and non-cumulative. All subjects should be well hydrated. Clinically significant electrolyte abnormalities should be corrected. Renal function must be monitored closely during treatment with carfilzomib. Serum chemistry values, including creatinine, must be obtained and reviewed prior to each dose of carfilzomib during Cycle 1 and prior to each course in subsequent cycles.
- Carfilzomib treatment can cause nausea, vomiting, diarrhea, or constipation sometimes requiring the use of antiemetics or antidiarrheals. Fluid and electrolyte replacement should be administered to prevent dehydration.

6.4.1 Guidelines For Monitoring, Prophylaxis, And Treatment Of Tumor Lysis Syndrome (TLS)

TLS, which may be associated with multiorgan failure, has been observed in treatment Cycles 1 and 2 in some patients with multiple myeloma who have been treated with carfilzomib. Patients receiving the cyclophosphamide and etoposide backbone alone can also develop TLS.

6.4.1.1 Hydration and Fluid Monitoring

Patients with leukemia or lymphoma not in CR at the time of treatment need to undergo TLS prophylaxis as per institutional protocol. This includes aggressive hydration and, if appropriate based upon laboratory values and other patient-specific issues, alkalinization. All patients with leukemia or lymphoma must be started on either allopurinol or rasburicase at the time of treatment initiation regardless of whether they have signs of TLS at that time. It is recommended that allopurinol or rasburicase be continued for at least 3-7 days after the start of therapy and only discontinued when there is no longer any laboratory evidence of tumor lysis syndrome.

6.4.1.2 Laboratory Monitoring

Patients should be closely monitored for TLS with frequent laboratory evaluation. The time interval between laboratory evaluations should be contingent upon each patient's baseline laboratory values and presumed tumor burden. Based upon an individual patient's TLS risk, it is recommended that TLS labs be checked every 4-12 hours during the first few days of treatment. At the local investigator's discretion, once the risk of TLS has decreased and laboratory values have normalized, laboratory evaluation frequency can be decreased, and then eventually discontinued once TLS is no longer a clinical concern. In the case of severe TLS development or for a patient deemed to be high risk for its development, ICU monitoring may be indicated.

6.4.1.3 Management of Tumor Lysis Syndrome

If TLS occurs, correct electrolyte abnormalities as appropriate, monitor renal function and fluid balance, and administer therapeutic and supportive care, including dialysis, as clinically indicated.

6.5 Concomitant Medications

Concomitant medication is defined as any prescription or over-the-counter preparation including vitamins and supplements. Patients must not receive any non-protocol chemotherapy, investigational therapy, or immunomodulating agents. Use of alternative medications (e.g. herbal or botanical, St John's Wort) is not permitted during the entire study period.

Patients may not receive corticosteroids other than those required in the protocol for carfilzomib premedication, to treat the "first dose effect" of fever, chills, rigors, and/or dyspnea, to treat acute allergic reactions, or for pre-medication for blood products when indicated for patients with history of severe reactions. Corticosteroids should not be used as antiemetics.

Prior hydroxyurea is permitted to control leukocytosis, but it must be discontinued at least 24 hours before beginning protocol therapy.

Patients should not receive any cardiac protectants such as dexrazoxane (Zinecard®) while being treated on protocol.

Female subjects of child-bearing potential must agree to use contraception for the duration of the study. Male subjects must agree to use a barrier method of contraception for the duration of the study if sexually active with a female of child-bearing potential. Abstinence is highly recom-

mended for pregnancy avoidance as well as patient safety secondary to patient's immunocompromised state and bleeding risk. For sexual activity, it is strongly recommended that 2 methods of birth control are used.

Concomitant medications should be recorded from 14 days before Day 1 through the end of the subject's study participation. Any change in concomitant medications must be recorded.

6.6 SUPPORTIVE CARE

6.6.1 Dexamethasone

Dexamethasone 0.1 mg/kg (max 4 mg) PO/IV will be administered prior to all carfilzomib doses during the 1st cycle. On days when patients are receiving cyclophosphamide and etoposide prior to carfilzomib, the dexamethasone should be administered along with other pre-medications prior to starting cyclophosphamide at hour 0. If treatment-related fever, rigors, chills, and/or dyspnea are observed post any dose of carfilzomib after dexamethasone has been discontinued for subsequent cycles, dexamethasone 0.1 mg/kg (max 4 mg) PO/IV should be re-started and administered prior to all subsequent doses.

6.6.2 Anti-emetics

All patients should receive pre-medication with anti-emetics following institutional guidelines. Further anti-emetic use should be given as needed. Patient should NOT receive corticosteroids as an anti-emetic.

6.6.3 Infection Control

Pneumocystis carinii prophylaxis: All patients are required to be on PCP prophylaxis as per institutional protocol. Prophylaxis should be continued through the study period and for a minimum of 3 months following protocol therapy. It is recommended that trimethoprim/sulfamethoxazole be used as frontline. If the patients are sulfa-allergic or have another reason that trimethoprim/sulfamethoxazole cannot be given, then alternatives include dapsone and pentamidine (IV or inhaled).

Fungal prophylaxis: All patients are required to receive systemic antifungal prophylaxis. Choice of medications will be made as per institutional guidelines, patient-specific factors, and based upon local flora.

Viral prophylaxis: Acyclovir or similar medication should be given to patients with a history of herpes simplex or zoster, per institutional prophylaxis guidelines, unless contraindicated.

Fever with Neutropenia: All patients with fever ≥38.3 on one occasion or ≥ 38.0 on 2 occasions at least an hour apart in a 24-hour period with ANC <500 are to be hospitalized and treated with broad spectrum antibiotics as per institutional guidelines and local infection profiles. Anti-fungal treatment should be considered for persistent fever for >3-5 days or emergence of new fever while on broad-spectrum antibiotics. Surveillance radiographic imaging for infection source should be strongly considered.

6.6.4 Blood Products

Blood products should be administered following institutional guidelines. All blood products administered should be recorded.

6.6.5 Gastrointestinal (GI) prophylaxis

While patients are undergoing dexamethasone pre-treatment for carfilzomib, they should also be given GI prophylaxis. It is recommended that a proton pump inhibitor be used instead of H2 blockers as H2 blockers can be myelosuppressive and concomitant use can worsen the myelosuppression of the study regimen.

6.6.6 G-CSF

Administration of short-acting G-CSF (Filgrastim, Neupogen®) or long-acting G-CSF (Pegfilgrastim, Neulasta®) is mandated starting on Day 6 of each cycle, between 24-36 hours after completion of carfilzomib administration. The choice of agent will be up to the local institution. However, please note that Pegfilgrastim (Neulasta®) use is not recommended for children under 40 kg. See Section 3.12 for further details.

6.6.7 Other Supportive Care

Any other necessary supportive care will be provided as needed for good clinical practice and per institutional guidelines

7 Study Tests and Observations

Evaluation	Screening	Cycle 1						Subsequent Cycles			End of Treatment
	Allowable number of days prior to enrollment	Day 1	Days 2, 3, 4, 5	Day 8 (± 1day)	Day 15 (± 1day)	Day 22 (± 1day)	Day 28 (± 1day)	Day 1 (- 2 days)	Day 2	Day 28 (+3days)	(+7 days)
Full Medical history	7 days	X						X			X
Physical/Interval history	7 days	X	X	X	X	X	X	X		X	X
Weight, Height, BSA	7 days	X						X			X
Performance Status	7 days							X			
CBC w/differential	7 days	X	X	X	X	X	X	X		X	X
Blood chemistry [a]	7 days	X	X	X	X	X	X	X		X	X
Urinalysis	7 days	X	X					X			X
PT/PTT/Fibrinogen	7 days			X[b]	X[b]	X[b]	X[b]				
Vital signs (T, HR, RR, BP) w/ Pulse oximetry	7 days	X	X	X[n]	X[n]	X	X	X[n]		X	X
Serum pregnancy[c]	7 days							X[c]			
Tumor assessment [d]	14 days						X[m]			X[d]	
Bone marrow [d,e]	14 days						X[f, g]			X[d, h]	X[f, g]
CSF Count [i]	14 days							X			
EKG & ECHO	14 days										
Correlative studies (optional) - blood[k]	N/A	X	X	X				X[l]	X[l]		
Correlative studies (optional)- bone marrow	14 days						X			X	
Correlative studies (optional)– tumor sample	Submit if available										
Buccal swab (optional)	14 days										
AE assessment [j]	Continuously throughout study period										

a. Blood chemistry to include: sodium, potassium, chloride, CO2, glucose, BUN, creatinine, calcium, albumin, alkaline phosphatase, ALT, AST, total bilirubin, direct bilirubin, total protein, uric acid, magnesium, phosphorus, and LDH. *For solid tumor patients ONLY: if uric acid is normal on Cycle 1, Day 1, it does not need to be repeated on Cycle 1, Days 2-5.
b. Repeat weekly if abnormal until resolved.
c. For females of childbearing potential; to be repeated every month. Must confirm result is negative prior to the start of each treatment cycle.
d. MRI/CT/PET/MIBG/Bone Marrow Aspirate/Biopsy as indicated for specific tumor/disease. Assessment tool used at screening should be used throughout the trial period. Tumor assessment for solid tumors and lymphomas to be done at time of screening, after Cycles 2, 4, 6, and then a minimum of every 3 cycles, unless otherwise clinically indicated. Bone marrow aspirates/biopsies are required at screening for solid tumor patients if disease type can have bone marrow involvement. Repeat bone marrow aspirates/biopsies must be performed for solid tumor patients only if bone marrow is positive for disease at screening, patient has delayed bone marrow recovery, or if otherwise clinically indicated. Disease evaluation window for all patients can be done day 28, +/-3 days (day 25-31) for subsequent cycles.
e. If bone marrow aspirate unattainable, then bone marrow biopsy may be substituted.
f. Bone marrow aspirate for patients with leukemia.
g. For leukemia patients, repeat bone marrow weekly if end-of-cycle response assessment cannot be made because bone marrow is hypocellular or cannot rule out G-CSF effect.
h. Leukemia patients are to have bone marrow aspirate +/- biopsy at the end of every treatment cycle.
i. For leukemia and Non-Hodgkin's lymphoma patients only. Can be done with bone marrow evaluation at the end of previous cycle to avoid second anesthesia. Patients with chronic myelogenous leukemia (CML) who are not in blast crisis and patients with juvenile myelomonocytic leukemia (JMML) do not need CSF evaluation prior to study enrollment nor during treatment and will not receive intrathecal chemotherapy.
j. Adverse Event assessment: Period of observation for collection of adverse events extends from informed consent signed until the end of treatment.
k. See Correlative Studies Time Points table (next page)
l. Cycle 2 only
m. Only for leukemia patients with extramedullary disease
n. For patients receiving carfilzomib on Days 8, 9, 15 and 16, vital signs w/ pulse oximetry must be obtained on those days
o. Urinalysis to include: Urine specific gravity, pH, Glucose, Ketones, Protein, Blood*, Nitrites*, Leukocyte esterase* (*If results are abnormal, a microscopic exam is required and must include results for red blood cells, white blood cells, and bacteria).
p. Physical exam to include: General, HEENT, Lungs, Cardiovascular, Abdomen, Lymph nodes, Skin, Musculoskeletal, Genitalia, Neurological, and Psychological.

7.1 Blood Correlative Studies Time Points

See Appendix C for details of samples to be collected and shipping requirements as well as shipping forms to accompany samples.

Note that blood and bone marrow samples should not be batched, but sent on Mon-Thurs on the same day as collected. If it is a weekend or holiday, store in refrigerator then ship the sample on next available business day.

**CYCLE 1:**	
Day 1	Hour 0 (pre-chemo)
Day 1	Hour 3 (between end of chemo and start of carfilzomib)
Day 2	Hour 0 (pre-chemo)
Day 3	Hour 0 (pre-chemo)
Day 8	If the patient is receiving carfilzomib on Day 8, then the sample should be drawn prior to administering the carfilzomib dose. If the patient is not receiving carfilzomib that day, then the sample can be drawn at any time.

**CYCLE 2:**	
Day 1	Hour 0 (pre-chemo)
Day 2	Hour 0 (pre-chemo)

8 Study Discontinuation

8.1 Criteria for Removal from Protocol Therapy

a. Lack of response to treatment therapy
b. Progressive disease
c. Relapse in any site following remission
d. Second malignant neoplasm
e. Completion of protocol therapy
f. Patient/parent withdrawal or refusal after beginning protocol therapy
g. Patient/parent withdrawal or refusal before beginning protocol therapy
h. Patient off treatment for other complicating disease
i. Non-compliance with protocol regimen and procedures
j. Unacceptable toxicity
k. Investigator determination
l. Female patient becomes pregnant or begins breast-feeding
m. Patient pursues alternative treatment (i.e. BMT or other, specify)

8.2 Off Study Criteria

a. Death
b. Patient Lost to follow-up
c. Parent/Patient withdraws consent, refuses follow-up
d. Fifth anniversary of study's closure to accrual.

8.3 Termination of the Study by POETIC

POETIC may terminate this study prematurely, either in its entirety or at an investigative site, for reasonable cause provided that written notice is submitted in advance of the intended termination. Advance notice is not required if the study is stopped due to safety concerns.

9 Adverse Events

9.1 Adverse Events Definitions

An AE is any untoward medical occurrence in a study subject administered an investigational product and that does not necessarily have a causal relationship with this treatment.

An AE therefore can be any unfavorable and unintended sign (including laboratory finding), symptom or disease temporally associated with participation in an investigational study, whether or not considered drug-related. In addition to new events, any increase in the severity or frequency of a pre-existing condition that occurs after the subject signs a consent form for participation is considered an AE. This includes any side effect, injury, toxicity, or sensitivity reaction.

An unexpected AE is any adverse drug event, the specificity or severity of which is not consistent with the current IB or prescribing information for a marketed compound. Also, reports which add significant information on specificity or severity of a known, already documented AE constitute unexpected AEs. For example, an event more specific or more severe than described in the IB would be considered "unexpected".

Whenever possible, the Common Terminology Criteria for Adverse Events (CTCAE) version 4.03 should be used to describe the event and for assessing the severity of AEs (see Appendix A). Any events representing a change in the CTCAE Grade need to be reported on the AE case report form. This includes any change in laboratory values.

For AEs not adequately addressed in the CTCAE 4.03, the severity table below may be used:

Severity	Description
GRADE 1 – Mild	Transient or mild discomfort; no limitation in activity; no medical intervention/therapy required.
GRADE 2 – Moderate	Mild to moderate limitation in activity—some assistance may be needed; no or minimal medical intervention/therapy required.
GRADE 3 – Severe	Marked limitation in activity, some assistance usually required; medical intervention/therapy required, hospitalizations possible.
GRADE 4 – Life-threatening	Extreme limitation in activity, significant assistance required; life-threatening (immediate risk of death); significant medical intervention/therapy required, hospitalization or hospice care probable.
GRADE 5 – Fatal	Death

Any condition, laboratory abnormality, or physical finding with an onset date prior to the subject signing consent for study participation is considered to be pre-existing in nature and part of the subject's medical history.

9.2 Causality

Using the following criteria, the relationship of the AE to the study drug should be assessed as follows:

- Definite - The adverse event *is clearly related* to the study drug.
- Probable - The adverse event *is likely related* to the study drug.
- Possible - The adverse event *may be related* to the study drug.
- Unlikely - The adverse event *is doubtfully related* to the study drug.
- Unrelated - The adverse event *is clearly NOT related* to the study drug.

9.3 Adverse Events Reporting Procedures

All AEs (e.g., any new event or worsening in severity or frequency of a pre-existing condition or laboratory finding) with an onset date after the subject receives first dose of the study drug must be promptly documented on the appropriate summary. Details of the event must include severity, relationship to study drug, duration, action taken, and outcome. Serious adverse events (SAEs) will be recorded on the appropriate form.

All AEs that are considered related to study drug must be followed to resolution or stabilization if improvement is not expected.

AEs should be reported from the time the subject receives the first dose of study drug through 30 days post-last dose of study drug or initiation of a new anti-cancer therapy, whichever occurs first. In addition, the Investigator should report any AE that may occur after this time period that is believed to have a reasonable possibility of being associated with study drug. If a subject is randomized but discontinues study prior to receiving any study drug, AEs must be reported through the end-of-study visit. AEs which completely resolve and then recur should be recorded as a new AE. For subjects who complete the end of study visit less than 30 days following their last dose of study drug, a follow up of ongoing AEs should be attempted by telephone, and documented in the subject’s source. AEs continuing at 30 days post-last dose should have a comment in the source by the Investigator that the event has stabilized or is not expected to improve.

The Principal Investigator is responsible for evaluating all AEs, obtaining supporting documents, and determining that documentation of the event is adequate. Adverse events will be assigned a severity grade using the NCI-CTCAE grading scale v4.03.

All AEs must be recorded on the CRF.

The Principal Investigator may delegate these duties to Sub-investigators and must ensure that these Sub-investigators are qualified to perform these duties under the supervision of the Principal Investigator and that they are listed on the FDA Form 1572.

9.4 Serious Adverse Events Definitions

An SAE is one that meets the following criteria:

- Death
- Life threatening experience defined as any adverse experience that places the subject, in the view of the Investigator, at immediate risk of death at the time of occurrence; i.e., it does not include a reaction that, had it occurred in a more severe form, might have caused death.
- Requires inpatient hospitalization or prolongation of an existing hospitalization (except scheduled hospitalizations for non-acute, unrelated cause such as an elective surgery)
- Results in persistent or significant disability/incapacity
- Is a congenital anomaly/birth defect in the offspring of an exposed subject
- Important medical events that may not result in death, be life-threatening, or require hospitalization, may be considered an SAE, when, based upon appropriate medical judgment, it jeopardizes the subject and may require medical or surgical intervention to prevent one of the outcomes listed in this definition.

Any death occurring within 30 days of the subject receiving study drug, regardless of the subject having discontinued from the study must be reported to the Sponsor and the POETIC DCC as an SAE.

9.5 Serious Adverse Event Reporting and Documentation Requirements

The Study Principal Investigator is responsible for monitoring the safety of patients who enroll in the study. All adverse events (AEs) and Serious Adverse Events (SAEs) occurring after any administration of the study drug regardless of drug attribution will be followed to the end of the study including 30 days after the last administration of the study drug, as well as any SAEs designated possibly, probably, or definitely related to treatment that occur greater than 30 days.

The descriptions and grading scales found in the revised NCI Common Terminology Criteria for Adverse Events (CTCAE) version 4.03 will be used for adverse event and serious adverse event reporting. All participating sites should have access to a copy of the CTCAE version 4.03. A copy of the CTCAE version 4.03 can be downloaded from the CTEP web site (http://ctep.cancer.gov/reporting/ctc.html).

The Study Site Investigator is required to report all adverse events that occur during the clinical study starting with the first dose of study drug throughout 30 days of stopping the investigational agent. Serious and non-serious adverse events will be discussed with the participating sites during regularly scheduled study meetings.
Severe adverse events must be reported to the appropriate protocol-defined study sponsors. Serious adverse events must be reported either by telephone or in person to the Study Principal Investigator, POETIC DCC, and local IRB within 24 hours of knowledge of their occurrence. SAE report form and source documentation must be sent to the Study Principal Investigator and the POETIC DCC within 3 calendar days. Additionally, the Serious Adverse Event should be entered into the

Electronic Data Capture (EDC). The Study Site Investigator is responsible for submitting follow-up reports for all SAEs that occurred for study participants under their care regarding the patient's subsequent course until the SAE has resolved or until the patient's condition stabilizes (in the case of persistent impairment), or the patient dies.

Reporting requirements for adverse events that occur on treatment and within 30 days[1] of the last dose of study drug.

	Grade 1	Grade 2		Grade 2		Grade 3		Grade 3		Grades 4 & 5
	Unexpected and Expected	Unexpected		Expected		Unexpected		Expected		
	with or without hospitalization	with Hospitalization	without Hospitalization	with Hospitalization	without Hospitalization	with Hospitalization	without Hospitalization	with Hospitalization	without Hospitalization	Unexpected and Expected
Unrelated Unlikely	Not Required	SAE Report Required	Not Required	SAE Report Required	Not Required	SAE Report Required	Not Required	SAE Report Required	Not Required	SAE Report Required
Possible Probable Definite	Not Required	SAE Report Required	SAE Report Required	SAE Report Required	Not Required	SAE Report Required	SAE Report Required	SAE Report Required	Not Required	SAE Report Required

[1] Adverse events with attribution of possible, probable, or definite that occur greater than 30 days after the last dose of study treatment require an SAE report as follows:

- Grade 3 unexpected events with hospitalization or prolongation of hospitalization
- Grade 4 unexpected events
- Grade 5 expected events and unexpected events

The following toxicities, with or without hospitalization, are EXCEPTIONS, and do NOT require expedited reporting on this trial:

- Grade 1 – 4 hemoglobin, white blood cells, lymphocytes, neutrophils (ANC), or platelets
- Grade 1 – 3 nausea, vomiting, diarrhea, anorexia, fatigue/malaise, weight loss
- Grade 1 – 4 alopecia
- Grade 1 – 4 AST, ALT, GGT that returns to ≤ Grade 1 or baseline within 42 days of the start of therapy
- Grade 1 – 4 fever, febrile neutropenia, with the exception of Zoster (Varicella) or herpetic infections
- Grade 1 – 3 hyperglycemia
- Grade 1 – 3 hypokalemia, hypophosphatemia, hypomagnesemia
- Grade 1 – 3 mucositis/stomatitis
- Grade 1 – 4 metabolic/electrolyte abnormalities attributable to tumor lysis syndrome or its treatment (Na, K, HCO_3, Ca^{++}, PO_4, creatinine, and uric acid) that resolves to ≤ Grade 1 or baseline within 42 days of the start of therapy
- Grade 3 hypertension that resolves to ≤ Grade 2 or baseline within 42 days of the start of therapy

Initial notification for all SAEs must include:

- Grade of event
- Date of event
- A brief description of the event
- Attribution to the investigational agent

- Patient Status

Relationship (definite, probable, possible, unlikely, unrelated) of any adverse event to *study drug* should use the following criteria in Section 9.2.

SAEs that occur at a site must be reported within 24 hours of knowledge by phone or e-mail to:

- POETIC DCC
- Study Principal Investigator of the protocol
- Local IRB, according to their institutional policy

The POETIC DCC must report individual site SAEs received via email within 3 days to:

- All participating POETIC sites

Participating POETIC sites where the SAE did **NOT** occur must report an SAE within 3 days to:

- Local IRB, according to their institutional policies.

The Study Principal Investigator of the protocol must report SAEs that are considered Suspected, Unexpected Serious Adverse Reaction (SUSARs) within 7-15 days to:

- Amgen, see details in section ‘Expedited reporting’ below
- The Food and Drug Administration (FDA), see details in section ‘Expedited reporting’ below

SAE contact information for the POETIC DCC is listed below:

POETIC Data Coordinating Center
Pediatric Hematology/Oncology
Stanford University
455 Broadway Ave.
Redwood City, CA 94063.
Phone: 650-736-0269
Fax: 650-736-3731
Email: poeticdcc@stanford.edu

Contact information for the Primary Investigators is listed below:

Study Principal Investigators:

Norman J. Lacayo, MD
Stanford University School of Medicine and Stanford Cancer Institute
Lucile Packard Children’s Hospital at Stanford
1000 Welch Rd., Ste. 300
Palo Alto, CA 94304
Phone: 650-723-5533
Fax: 650-723-5231
Email: lacayon@stanford.edu

Anne-Marie Langevin, MD
University of Texas Health Science Center at San Antonio
7703 Floyd Curl Drive, MC7810
San Antonio, TX 78229
Phone: 210-567-7460
Fax: 210-567-7466
Email: langevin@uthscsa.edu

Aru Narendran, MD, PhD
Alberta Children's Hospital
28 Oki Drive NW
Calgary, Alberta T3B 6A8
CANADA
Phone: 403-210-6418
Fax: 403-955-7684
Email: a.narendran@ucalgary.ca

9.6 Expedited Reporting

The Study Principal Investigator must inform Amgen in writing by Fax at the contact information listed below of all Expedited Safety Reports submitted to the relevant Regulatory Agencies. These notifications should be performed in parallel to the Regulatory Agency submissions (e.g., within 7 calendar days for any Fatal or Life-threatening SUSARs and within 15 calendar days for all other SUSARs), but in no case any later than 1 business day from the submission date. This must be documented on FDA 3500A MEDWATCH or CIOMS I (for EU studies) form. This form must be completed and supplied to Amgen in English.

The Study Principal Investigator should submit reports within 7 calendar days to the FDA by a rapid means of communication for any unexpected fatal or life-threatening suspected adverse reactions, preferably by fax or email (for email, obtain a secure email account with FDA. Information in the IND acknowledgment letter. For fax, see contact information below). Each submission should be addressed to the Regulatory Project Manager and/or the Chief, Project Management Staff. Cite the IND number of this study #126230 at the top of the first page of any communications concerning this application.

The initial report must be as complete as possible, at a minimum including the serious adverse event term (s), patient identifier, date of awareness of the event, an assessment of the causal relationship between the event and the investigational product(s), and name of the reporter (investigator). Information not available at the time of the initial report (e.g., an end date for the adverse event or laboratory values received after the report) must be documented on a follow-up MEDWATCH or CIOMS I form and submitted to Amgen in the same timelines as outlined above. The Amgen protocol number (20159889/IST-CAR-2013-100660) and the institutional protocol number should be included on all reports to Amgen.

All other SAE's will be sent to Amgen on a biannual basis in the form of a line listing in English. The line listing must include the following information; patient initials, date of birth, sex, SAE onset date, SAE stop date, event name (term), outcome, date of first dose of study drug(s), date

of last dose of study drug(s) prior to the event, action taken with study drug(s) the Investigator's assessment of causality (relationship to carfilzomib), and the Investigator's assessment of expectedness to carfilzomib. The sponsor reserves the right to review the CRFs or source documents in response to any inquires by regulatory agencies that the sponsor may receive.

Amgen Global Safety Contact Information:

Amgen
Fax: 888-814-8653 (Toll-Free, Us Only)
+44-20-7136-1046 (Toll, Global)
E-mail: Svc-Ags-In-Us@Amgen.Com

FDA Safety Contact Information:

FDA
Food and Drug Administration
Center for Drug Evaluation and Research
Division of Hematology Products
5901-B Ammendale Road
Beltsville, MD 20705-1266
Phone: (301) 796-7550
Fax: (301) 796-9845

In accordance with local regulations, Amgen will notify the Study Principal Investigator in writing of new safety information regarding the study drug Carfilzomib when issued via Amgen's Global Safety Notification (GSN) Intralinks Portal or via their Shared Investigator Platform (SIP) (ie, not previously described in the IBs) .

Other important findings which may be reported by Amgen in writing include: increased frequency of a clinically significant expected SAE, an SAE considered associated with study procedures that could modify the conduct of the study, lack of efficacy that poses significant hazard to study subjects, clinically significant safety finding from a nonclinical (eg, animal) study, important safety recommendations from a study data monitoring committee, or sponsor decision to end or temporarily halt a clinical study for safety reasons.

Upon receiving any new safety information from Amgen, the Study Principal Investigator must review and retain any memo, letter, report, or Investigator Brochure Amendments and provide this information to the POETIC DCC to be distributed to all POETIC sites by POETIC DCC. Where required by local regulations for the study, each POETIC site will submit the safety information to the appropriate IRB. Study Principal Investigator and local IRB will determine if the informed consent and protocol requires revision.

The Study Principal Investigator will notify the FDA in an IND safety report of potentially serious risks from clinical trials or any other clinical, animal, or in-vitro studies that suggest significant human risk as soon as possible, but no later than 15 calendar days after the sponsor receives the safety information and determines that the information qualifies for reporting.

9.7 Pregnancy

If a subject or spouse or partner of a subject becomes pregnant while enrolled in this clinical trial or up to three months following administration of carfilzomib, the Study Principal Investigator, the POETIC DCC and Amgen Global Safety must be notified within 24 hours of the Investigator, designee, or site personnel learning of the pregnancy (See Amgen Global Safety contact information above). If the subject is pregnant, carfilzomib and all other chemotherapy treatment must be withheld.

Subjects, spouses, or partners will be followed through the outcome of the pregnancy. The Investigator will be required to report the outcome to the POETIC DCC and Amgen Global Safety.

If the outcome of the pregnancy meets a criterion for immediate classification as an SAE—spontaneous abortion (any congenital anomaly detected in an aborted fetus is to be documented), stillbirth, neonatal death, or congenital anomaly—the Investigator should repeat the procedures for expedited reporting of SAEs as outlined above.

10 Statistical Analysis

10.1 Study Design

10.1.1 Dose Escalation and Determination of Maximum Tolerated Dose (MTD)

Dose escalation will be managed independently for each of the two strata (A and B). DLTs will be assessed for each patient during the 1st cycle only.

The rolling six phase 1 trial design will be used for the conduct of this study. [19] Two to six patients can be concurrently enrolled onto a dose level, dependent upon (1) the number of patients enrolled at the current dose level, (2) the number of patients who have experienced DLT at the current dose level, and (3) the number of patients entered but with tolerability data pending at the current dose level. Accrual is suspended when a cohort of six has enrolled or when the study endpoints have been met.

Dose level assignment is based on the number of participants currently enrolled in the cohort, the number of DLTs observed, and the number of participants at risk for developing a DLT (i.e., participants enrolled but who are not yet assessable for toxicity). For example, when three participants are enrolled onto a dose cohort, if toxicity data is available for all three when the fourth participant entered and there are no DLTs, the dose is escalated, and the fourth participant is enrolled to the subsequent dose level. If data is not yet available for one or more of the first three participants and no DLT has been observed, or if one DLT has been observed, the new participant is entered at the same dose level. Lastly, if two or more DLTs have been observed, the dose level is de-escalated. This process is repeated for participants five and six. In place of suspending accrual after every three participants, accrual is only suspended when a cohort of six is filled. When participants are inevaluable for toxicity, they are replaced with the next available participant if escalation or de-escalation rules have not been fulfilled at the time the next available participant is enrolled onto the study.

The following table provides the decision rules for enrolling a patient at (i) the current dose level (ii) at an escalated dose level, (iii) at a de-escalated dose level, or whether the study is suspended to accrual:

# Pts Enrolled	# Pts with DLT	# Pts without DLT	# Pts with Data Pending	Decision
2	0 or 1	0, 1 or 2	0, 1 or 2	Same dose level
2	2	0	0	De-escalate*
3	0	0, 1 or 2	1, 2 or 3	Same dose level
3	1	0, 1 or 2	0, 1 or 2	Same dose level
3	0	3	0	Escalate**
3	≥ 2	0 or 1	0 or 1	De-escalate*
4	0	0, 1, 2 or 3	1, 2, 3 or 4	Same dose level
4	1	0, 1, 2 or 3	0, 1, 2 or 3	Same dose level
4	0	4	0	Escalate**
4	≥ 2	0, 1 or 2	0, 1 or 2	De-escalate*
5	0	0, 1, 2, 3 or 4	1, 2, 3, 4 or 5	Same dose level
5	1	0, 1, 2, 3 or 4	0, 1, 2, 3 or 4	Same dose level
5	0	5	0	Escalate**
5	≥ 2	0, 1, 2 or 3	0, 1, 2 or 3	De-escalate*

6	0	0, 1, 2, 3, or 4	2, 3, 4, 5 or 6	Suspend
6	1	0, 1, 2, 3 or 4	0, 1, 2, 3 or 4	Suspend
6	0 or 1	5 or 6	0 or 1	Escalate**
6	≥ 2	0, 1, 2, 3 or 4	0, 1, 2, 3 or 4	De-escalate*

If two or more of a cohort of up to six patients experience DLT at a given dose level then the MTD has been exceeded and dose escalation will be stopped with the exception of the following:

In the event that two DLTs observed out of 6 evaluable patients are different classes of Adverse effects (e.g. hepatotoxicity and myelosuppression), expansion of the cohort to 12 patients will be considered if all of the following conditions are met:

- One of the DLTs does not appear to be dose-related
- The Adverse effects are readily reversible
- The study chair, or co-investigator, and POETIC Scientific Leadership all agree that expansion of the cohort is acceptable and important to preserve the integrity of the study and the safety of the patients enrolled or planned to be enrolled. Adverse event data will be monitored in real time by these individuals. This information will be reviewed in real time in order to make decisions regarding cohort expansion, escalation, or de-escalation.

If fewer than 1/3 of patients in the expanded cohort experience dose-limiting toxicities, the dose escalation can proceed.

10.1.2 Outcome Assessment/Therapeutic Response

A. Leukemia Response Assessment

- **Complete Remission (CR):** Attainment of an M1 bone marrow (< 5% blasts) with no evidence of circulating blasts or extramedullary disease and with recovery of blood counts (ANC≥ 1000/µL and platelet count ≥ 100,000/µL). Occasionally, a rare blood blast may be identified during marrow regeneration; however, the marrow must be M1 status.

- **Complete remission with incomplete platelet recovery (CRp):** Attainment of an M1 bone marrow (< 5% blasts), no evidence of circulating blasts or extramedullary disease and with ANC ≥1000/µL and platelet count < 100,000/µL

- **Complete remission with incomplete blood count recovery (CRi):** Attainment of an M1 bone marrow (< 5% blasts), no evidence of circulating blasts or extramedullary disease and with ANC < 1000/µL or platelet count < 100,000/µL

- **Partial Response (PR)**: A decrease of at least 50% in the percentage of blasts to 5% to 25% (M2 marrow) in the bone marrow aspirate. For patients with extramedullary disease only, a decrease in disease amount of at least 30%. Bone marrow must have adequate cellularity with normal hematopoietic progenitors to determine response. A repeat bone marrow aspiration weekly may be required to distinguish between a PR and increased blasts caused by bone marrow regeneration.

- **Progressive Disease (PD):** An increase of at least 25% of the absolute number of bone marrow or circulating leukemic blasts, development of new extramedullary disease or an increase in existing extramedullary disease by 20% or other laboratory or clinical evidence of progression.
- **Relapse:** Morphologic relapse after CR/CRp/CRi is defined as a reappearance of leukemic blasts in the blood or ≥ 5% blasts in the bone marrow not attributable to any other cause (e.g., bone marrow regeneration)

- **Stable Disease (SD):** Patient fails to qualify for CR, CRi, CRp, PR, PD, or relapse

- **Unevaluable (U):** Aplastic or severely hypocellular marrow (<10-20% cellularity) with any blast percentage. In this instance, marrow evaluation should be repeated weekly until response determination can be made.

*Note the use of G-CSF in this protocol can confound disease assessment as there may be excessive bone marrow blasts (as well as blood blasts) secondary to rapid bone marrow regeneration. If an elevated blast count is thought possibly related to G-CSF, then a repeat bone marrow aspirate should be performed weekly until an adequate assessment can be made. It is recommended that there be at least 48 hours, ideally > 7 days, between the final dose of G-CSF and the bone marrow assessment.

<u>Bone Marrow Classification:</u>
M1 is < 5% blasts
M2 is 5 to 25% blasts
M3 is > 25% blasts

B. Solid Tumor Response Assessment

For patients with measurable disease, response and progression will be evaluated in this study using the revised Response Evaluation Criteria in Solid Tumors (RECIST) guideline (version 1.1). [20] Key points are that 5 target lesions are identified and that changes in the *largest* diameter (unidimensional measurement) of the tumor lesions but the *shortest* diameter of malignant lymph nodes are used in the RECIST v 1.1 criteria.

For patients without measurable disease, but disease present by PET, MIBG, or bone marrow evaluation will be evaluated for response as described in the relevant sections below.

RECIST

Measurable Disease

Measurable lesions are defined as those that can be accurately measured in at least one dimension (longest diameter in the plane of measurement is to be recorded) with a minimum size of:

- 10 mm by CT scan (CT scan slice thickness no greater than 5 mm.)
- 10 mm caliper measurement by clinical exam (lesions which cannot be accurately measured with calipers should be recorded as non-measurable).
- 20 mm by chest X-ray.

All tumor measurements must be recorded in millimeters (or decimal fractions of centimeters). Malignant lymph nodes: To be considered pathologically enlarged and measurable, a lymph node must be > 15 mm in short axis when assessed by CT scan (CT scan slice thickness recommended to be no greater than 5 mm). At baseline and in follow-up, only the short axis will be measured and followed.

Non-measurable Disease

This includes all other lesions, including small lesions (longest diameter < 10 mm or pathological lymph nodes with > 10 to < 15 mm short axis) as well as truly non-measurable lesions. Lesions considered truly non-measurable include: leptomeningeal disease, ascites, pleural or pericardial effusion, inflammatory breast disease, lymphangitic involvement of skin or lung, abdominal masses/ abdominal organomegaly identified by physical exam that is not measurable by reproducible imaging techniques.

Target Lesions

All measurable lesions up to a maximum 2 lesions per organ and 5 lesions in total, representative of all involved organs, should be identified as target lesions and recorded and measured at baseline. Target lesions should be selected on the basis of their size (lesions with the longest diameter), be representative of all involved organs, and should be those that are suitable for reproducible repeated measurement (either by imaging techniques or clinically). If the largest lesion does not lend to reproducible measurement, the next largest lesion which can be measured reproducibly should be used. A sum of diameters (longest for non-nodal lesions, short axis for nodal lesions) for all target lesions will be calculated and reported as the baseline sum diameters. If lymph nodes are to be included in the sum, only the short axis is added to the sum. The baseline sum diameters

will be used as a reference to further characterize any objective tumor regression in the measurable dimension of the disease.

Non-target Lesions

All other lesions (or sites of disease) should be identified as non-target lesions and should also be recorded at baseline. Measurements of these lesions are not required, but the presence or absence of each, or in rare instances unequivocal progression of each, should be noted throughout follow-up. It is possible to record multiple non- target lesions involving the same organ as single item.

Guidelines for Evaluation of Measurable Disease

All measurements should be taken and recorded in metric notation using a ruler or calipers. All baseline evaluations should be performed as closely as possible and never more than 4 weeks before study enrollment. [However, for the purposes of this trial, the baseline evaluation will need to be performed within 14 days of study enrollment.] Tumors in a previously irradiated area will be considered measurable.

The same method of assessment and the same technique should be used to characterize each identified and reported lesion at baseline and during follow-up. Imaging based evaluation should always be done rather than clinical examination unless the lesion(s) being followed cannot be imaged but are assessable by clinical exam.

Clinical lesions: Clinical lesions will only be considered measurable when they are superficial and > 10 mm diameter as assessed using calipers (e.g. skin nodules). For the case of skin lesions, documentation by color photography including a ruler to estimate the size of the lesion is suggested. As noted above, when lesions can be evaluated by both clinical exam and imaging, imaging evaluation should be undertaken since it is more objective and may also be reviewed at the end of the study.

- **Chest X-ray:** Chest CT is preferred over chest X-ray, particularly when progression is an important endpoint, since CT is more sensitive than X-ray, particularly in identifying new lesions. However, lesions on chest X-ray may be considered measurable if they are clearly defined and surrounded by aerated lung.

- **CT, MRI:** CT is the best currently available and reproducible method to measure lesions selected for response assessment. This guideline has defined measurability of lesions on CT scan based on the assumption that CT slice thickness is 5 mm or less. When CT scans have slice thickness greater than 5 mm, the minimum size for a measurable lesion should be twice the slice thickness. MRI is also acceptable in certain situations (e.g. for body scans).

- **Ultrasound:** Ultrasound is not useful in assessment of lesion size and should not be used as a method of measurement. Ultrasound examinations cannot be reproduced in their entirety for independent review at a later date and, because they are operator dependent, it cannot be guaranteed that the same technique and measurements will be taken from one assessment to

the next. If new lesions are identified by ultrasound in the course of the study, confirmation by CT or MRI is advised. If there is concern about radiation exposure at CT, MRI may be used instead of CT in selected instances.

- **Endoscopy, laparoscopy:** The utilization of these techniques for objective tumor evaluation is not advised. However, they can be useful to confirm complete pathological response when biopsies are obtained or to determine relapse in trials where recurrence following complete response or surgical resection is an endpoint.

- **Tumor markers:** Tumor markers alone cannot be used to assess objective tumor response. If markers are initially above the upper normal limit, however, they must normalize for a patient to be considered in complete response. Because tumor markers are disease specific, instructions for their measurement should be incorporated into protocols on a disease specific basis.

- **Cytology, histology:** These techniques can be used to differentiate between PR and CR in rare cases if required by protocol (for example, residual lesions in tumor types such as germ cell tumors, where known residual benign tumors can remain). When effusions are known to be a potential adverse effect of treatment (e.g. with certain taxane compounds or angiogenesis inhibitors), the cytological confirmation of the neoplastic origin of any effusion that appears or worsens during treatment can be considered if the measurable tumor has met criteria for response or stable disease in order to differentiate between response (or stable disease) and progressive disease.

Evaluation of Target Lesions

- **Complete Response (CR):** Disappearance of all target lesions. Any pathological lymph nodes (whether target or non-target) must have reduction in short axis to <10 mm.

- **Partial Response (PR):** At least a 30% decrease in the sum of the diameters of target lesions, taking as reference the baseline sum diameters.

- **Progressive Disease (PD):** At least a 20% increase in the sum of the diameters of target lesions, taking as reference the smallest sum on study (this includes the baseline sum if that is the smallest on study). In addition to the relative increase of 20%, the sum must also demonstrate an absolute increase of at least 5 mm. (Note: the appearance of one or more new lesions is also considered progression).

- **Stable Disease (SD):** Neither sufficient shrinkage to qualify for PR nor sufficient increase to qualify for PD, taking as reference the smallest sum diameters while on study.

Evaluation of Non-target Lesions

- **Complete Response (CR):** Disappearance of all non-target lesions and normalization of tumor marker level. All lymph nodes must be non-pathological in size (< 10 mm short axis).

- **NonCR/NonPD:** Persistence of one or more non-target lesion(s) and/or maintenance of tumor marker level above the normal limits.

- **Progressive Disease (PD):** Unequivocal progression of existing non-target lesions. (Note: the appearance of the one or more new lesions is also considered progression.).

Although a clear progression of “non-target” lesions only is exceptional, in such circumstances the opinion of the treating physician should prevail, and the progression status should be confirmed at a later time by the study chair.

Note: If tumor markers are initially above the upper normal limit, they must normalize for a patient to be considered in complete clinical response.

Evaluation of Best Overall Response

The best overall response is the best response recorded from the start of the treatment until the end of treatment taking into account any requirement for confirmation. The patient's best response assignment will depend on the achievement of both measurement and confirmation criteria.

Overall Response Assessment for Solid Tumors

Target Lesions	Non-target Lesions	New Lesions	Overall Response
CR	CR	No	**CR**
CR	Non-CR/non-PD	No	**PR**
CR	Not evaluated	No	**PR**
PR	Non-PD or not all evalu-	No	**PR**
SD	Non-PD or not all evalu-	No	**SD**
Not all evaluated	Non-PD	No	**Not evaluated**
PD	Any	Yes or No	**PD**
Any	PD	Yes or No	**PD**
Any	Any	Yes	**PD**

Note: Patients with a global deterioration of health status requiring discontinuation of treatment without objective evidence of disease progression at that time should be classified as having "symptomatic deterioration." Every effort should be made to document the objective progression, even after discontinuation of treatment.

In some circumstances, it may be difficult to distinguish residual disease from normal tissue. When the evaluation of complete response depends on this determination, it is recommended that the residual lesion be investigated (fine needle aspirate/biopsy) before confirming the complete response status.

Incorporating Response Criteria for Patients by FDG-PET

In certain cases, it is reasonable to incorporate the use of FDG-PET scanning to complement CT scanning in assessment of progression (particularly possible 'new' disease).[21] New lesions on the basis of FDG- PET imaging can be identified according to the following algorithm:

Negative FDG-PET at baseline, with a positive FDG-PET at follow- up is a sign of PD based on a new lesion. If the positive FDG-PET at follow-up corresponds to a new site of disease confirmed by CT, this is PD. If the positive FDG-PET at follow-up is not confirmed as a new site of disease on CT, additional follow-up CT scans are needed to determine if there is truly progression occurring at that site (if so, the date of PD will be the date of the initial abnormal FDG-PET scan). If the positive FDG-PET at follow-up corresponds to a pre-existing site of disease on CT that is not progressing on the basis of the anatomic images, this is not PD.

<u>Note</u>: A 'positive' FDG-PET scan lesion means one that is FDG avid with an uptake greater than twice that of the surrounding tissue on the attenuation corrected image.

<u>RESPONSE CRITERIA FOR PATIENTS WITHOUT MEASURABLE DISEASE:</u>

1. **<u>Neuroblastoma patients with MIBG positive lesions</u>**

MIBG Positive Lesions

Patients who have a positive MIBG scan at the start of therapy will be evaluable for MIBG response. The use of ^{123}I for MIBG imaging is recommended for all scans. If the patient has only one MIBG positive lesion and that lesion was radiated, a biopsy must be done at least 28 days after radiation was completed and must show viable neuroblastoma.

The following criteria will be used to report MIBG response:

- **Complete response (CR):** Complete resolution of all MIBG positive lesions
- **Partial Response (PR):** Resolution of at least one MIBG positive lesion, with persistence of other MIBG positive lesions
- **Stable disease (SD):** No change in MIBG scan in number of positive lesions
- **Progressive disease (PD):** Development of new MIBG positive lesions

The response of MIBG lesions should be assessed using the Curie scale as outlined below. The body is divided into 9 anatomic sectors for osteomedullary lesions, with a 10th general sector allocated for any extra-osseous lesion visible on MIBG scan**.** In each region, the lesions are scored as follows. The **absolute extension score** is graded as:

- 0 = no site per segment
- 1 = 1 site per segment
- 2 = more than one site per segment
- 3 = massive involvement (>50% of the segment)

The **absolute score** is obtained by adding the score of all the segments. See diagram of sectors below:

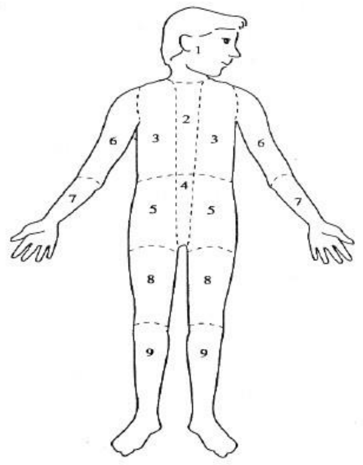

The **relative score** is calculated by dividing the absolute score at each time point by the corresponding pre-treatment absolute score. The relative score of each patient is calculated at each response assessment compared to baseline and classified as below:

- **Complete response:** all areas of uptake on MIBG scan completely resolved.

  If morphological evidence of tumor cells in bone marrow biopsy or aspiration is present at enrollment, no tumor cells can be detected by routine morphology on two subsequent bilateral bone marrow aspirates and biopsies done to be considered a **Complete Response**.

- **Partial response**: Relative score ≤ 0.2 (lesions almost disappeared) to ≤ 0.5 (lesions strongly reduced).

- **Stable disease**: Relative score > 0.5 (lesions weakly but significantly reduced) to 1.0 (lesions not reduced).

- **Progressive disease**: New lesions on MIBG scan.

Overall Best Response Assessment

Each patient will be classified according to his "best response" for the purposes of analysis of treatment effect.

2. **Solid tumor patients with bone marrow involvement**

Bone Marrow Involvement

Bone marrow obtained within 14 days prior to study enrollment with tumor cells seen on routine morphology (not by immunohistochemical staining only) of bone marrow aspirate or biopsy

Bone Marrow responses are determined by H&E Staining of bilateral bone marrow biopsies and aspirates.

- **Complete Response (CR)**: No tumor cells detectable by routine morphology and normalization of urinary catecholamines or other tumor markers if elevated at the time of study enrollment.

- **Progressive Disease (PD):** In patients who enroll with solid tumor infiltration in bone marrow by morphology have progressive disease if there is a doubling in the amount of tumor in the marrow AND a minimum of 25% tumor in bone marrow by morphology. (For example, a patient entering with 5% tumor in marrow by morphology must increase to ≥ 25% tumor to have progressive disease; a patient entering with 30% tumor must increase to > 60%). In patients who enroll without evidence of extrinsic cells in bone marrow will be defined as progressive disease if tumor is detected subsequent bone marrow biopsies or aspirations

- **Stable Disease (SD):** Persistence of tumor in bone marrow that does not meet the criteria for either complete response or progressive disease.

Overall Best Response Assessment

Each patient will be classified according to his "best response" for the purposes of analysis of treatment effect.

3. Response criteria for solid tumor patients with FDG-PET positive lesions without measurable disease

Response assessment is derived from EORTC Criteria [19]:

- **Complete Response (CR):** Disappearance of all known disease
- **Partial response (PR):** Estimated decrease of >=50%
- **Progressive Disease (PD):** Estimated increase of >=25% in existent lesions
- **Stable Disease (SD):** Neither CR, PR, or PD criteria met

C. Lymphoma Response Assessment

Lymphoma response assessment will utilize the Cheson Criteria as below [22]:

Response	Definition	Nodal Masses	Spleen, Liver	Bone Marrow
**CR**	Disappearance of all evidence of disease	(a)FDG-avid or PET positive prior to therapy; mass of any size permitted if PET negative<br>(b)Variable FDG-avid or PET negative; regression to normal size on CT	Not palpable, nodules disappeared	Infiltrate cleared on repeat biopsy; if indeterminate by morphology, immunohistochemistry should be negative
**PR**	Regression of measurable disease and no new sites	≥50% decrease in SPD of up to 6 largest dominant masses; no increase in size of other nodes<br>(a)FDG-avid or PET positive prior to therapy; one or more PET positive at previously involved site<br>(b)Variable FDG-avid or PET negative; regression on CT	≥50% decrease in SPD of nodules (for single nodule in greatest transverse diameter); no increase in size of liver or spleen	Irrelevant if possible prior to therapy; cell type should be specified
**SD**	Failure to attain CR/PR or PD	(a)FDG-avid or PET positive prior to therapy; PET positive at prior sites of disease and no new sites on CT or PET<br>(b)Variable FDG-avid or PET negative; no change in size of previous lesions on CT		
**Relapsed Disease or PD**	Any new lesion or increase by ≥ 50% of previously involved sites from nadir	Appearance of a new lesion(s) > 1.5 cm in any axis, ≥50% increase in SPD of more than one node, or ≥50% increase in longest diameter of a previously identified node > 1 cm in short axis<br>Lesions PET positive if FDG-avid lymphoma or PET positive prior to therapy	>50% increase from nadir in the SPD of any previous lesions	New or recurrent involvement

Abbreviations: CR, complete remission; FDG, [18F]fluorodeoxyglucose; PET, positron emission tomography; CT, computed tomography; PR, partial remission; PD, sum of the product of the diameters; SD, stable disease; PD, progressive disease.

10.1.3 CORRELATIVE STUDIES

The following schema summarizes the correlative studies planned at the laboratories of Phoenix Children's Hospital and Aru Narendran:

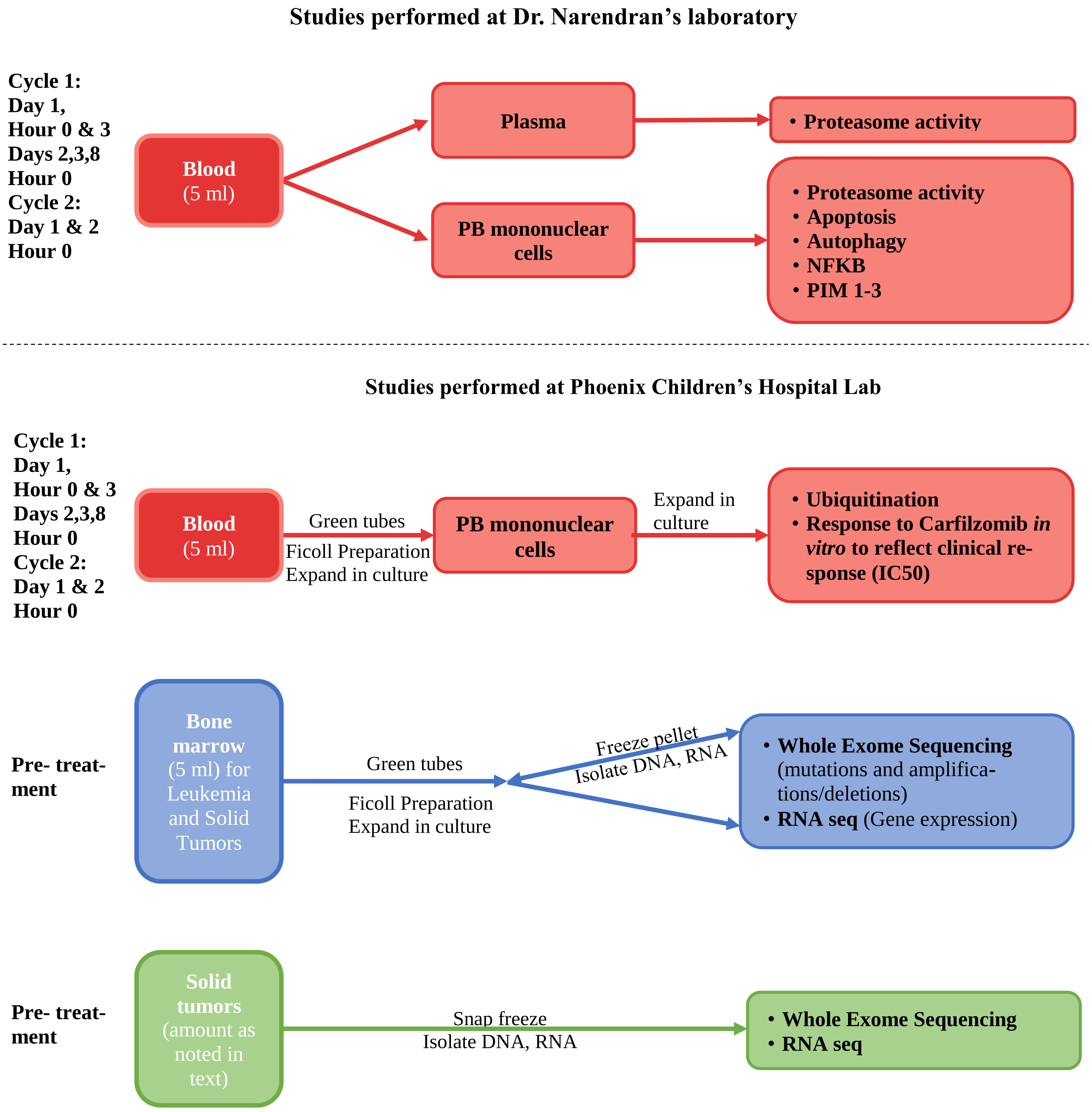

Laboratory of Aru Narendran, MD, PhD:

Previous studies have shown that intrinsic resistance to proteasome inhibitors directly correlates with basal levels of cellular proteasome activity and that pretreatment cellular proteasome levels may predict response to treatment. Secondly, elevated total circulating plasma proteasomes (cProt) levels post treatment has been shown to correlate with poorer response to therapy and decreased overall survival.[23] In addition, inhibition of proteasome activity results in alteration in a number of autophagy and apoptosis related proteins, providing an effective way to evaluate biological correlates of activity of agents such as carfilzomib. The studies here will focus on understanding these variables in plasma and circulating malignant cells in patients treated for hematological malignancies and plasma and circulating lymphocytes as surrogate markers in patients with solid tumors.

Sample collection and processing: 5ml of blood (for patients <10kg, 3mL blood is acceptable) will be collected in heparinized tubes just before the initiation of treatment with carfilzomib (baseline) and at defined periods during treatment (See Section 7 and Appendix C). These samples will be shipped by overnight courier to Narendran lab at the University of Calgary. Upon receipt, the mononuclear cells and plasma will be separated by an initial centrifugation step (1200 RPM for 5 min) followed by Percoll gradient centrifugation of the cellular fraction as per established protocols. The plasma samples will be immediately frozen and stored at -80°C until analysis. The mononuclear cells will be washed with cold PBS, counted and lysates will be made using appropriate buffers.

Plasma: Proteasome activity in plasma samples will be measured according to the method of Manasanch, et. al. based on the release of 7-amino-4-methyl coumarin (AMC).[24] Briefly, plasma samples will be activated with SDS for chymotrypsin and caspase like activities and with 10% Tween 20 for trypsin like activity. An aliquot of 10 uL of the activated plasma sample will be mixed with 30uL of assay buffer (25 mM HEPES), and 10 uL of flurogenic-AMC substrate. A multi-Mode Microplate Reader will be used to measure the fluorescent release of free AMC with time. All assays will be done in triplicate and statistical analyses will be performed using Mann Whitney test for comparison between independent groups.

Cells: Various proteasome activities in intact cells will be measured using commercially available kits (Trypsin-like and Caspase-like Cell-Based Assays, Promega, Madison, WI, U.S.A.) according to manufacturer's protocol. Briefly, 5000 cells/well will be taken in 96 well plates and appropriate substrates (for the chymotrypsin-like, trypsin-like and caspase-like activities, Suc-LLVY-aminoluciferin, Z-LRR-aminoluciferin and Z-nLPnlD-aminoluciferin, respectively) will be added.[25] After 10 min of incubation, luminescence will measured in an automatic plate reader. Observed activity levels will be standardized to original corresponding cell numbers.

Western Blot Analyses: Cells will be counted and lysed in RIPA buffer (125 mM Tris-HCl, pH 6.8) containing 4% sodium dodecyl sulfate (SDS) plus protease and phosphatase inhibitors (Sigma). Protein concentrations will be quantified by Bradford reagent (Bio-Rad Laboratories, Hercules, CA). After mixing with treatment buffer containing 2.5% β-mercaptoethanol, samples will be resolved by 8% SDS-PAGE. Proteins will then be transferred to nitrocellulose membranes, blocked with blotto buffer and probed with appropriate primary and secondary antibodies coupled to HRPO and luminol reagent. Blots will be exposed to double-emulsion films (Eastman Kodak, Rochester, NY) and the resulting

bands will be quantified by scanning and densitometry. Primary antibodies will include markers of apoptosis (Bcl-2, Mcl-1, BAX, PARP, caspase 3, caspase 9, caspase 10), autophagy (LC3 I, LC3 II, p62) and other known markers of target modulation such as NFKB and PIM1-3.

Laboratory of Phoenix Children's Hospital:

The studies performed will focus on (1) monitoring the activity of carfilzomib through the determination of downstream biomarkers of proteasome inhibition, and (2) developing *in vitro* and *in vivo* assays that reflect the patients' clinical response to carfilzomib, alone or in combination with the chemotherapy used in this protocol. From each patient, the following samples will be collected and assayed as described below: (1) Blood, (2) Bone marrow from patients with leukemia and solid tumors when done, (3) Tumor samples, if already available, from patients with solid tumors, (4) Buccal swabs

Sample collection and processing: 5ml of blood (for patients <10kg, 3mL of blood is acceptable) will be collected in tubes containing sodium heparin prior to the initiation of treatment and at defined periods during treatment (See Section 7 and Appendix C). 5 mL bone marrow in sodium heparin samples for patients with leukemia or with solid tumors with bone marrow involvement both pre- and post- treatment will also be collected. If the following are available, those will be submitted as well.

1. Snap frozen tissue for DNA/RNA extraction: at least 20 mg (wet weight) of tumor tissue that is representative of the malignancy (0.25 g is approximately 1.1 x 0.9 x 0.5 cm). (See Appendix C for shipping instructions and contact information for Matt Pankratz at the Phoenix Children's Hospital Laboratory regarding acceptable tumor samples).

All samples will be shipped by overnight courier to the Phoenix Children's Hospital Lab in Phoenix, AZ.

Analysis of ubiquitination as a target of proteasome inhibition by carfilzomib directly in PBMCs (as a surrogate target source): The inhibition of CT-L activity by carfilzomib results in accumulation of ubiquitinated substrates. Therefore, the effect of administered carfilzomib will be studied by measuring levels of ubiquitination in PBMCs. PBMCs derived from patients (250,000-500,000 cells) will be lysed using modified RIPA buffer (50 mM Tris pH 7.4, 150 mM NaCl, 1% NP-40, 1 mM EDTA, 0.25% sodium deoxycholate) containing protease and phosphatase inhibitors (Pierce Biotechnology, Rockford, IL, USA) and treated with DNAse1 (Qiagen, Maryland, USA). Lysates will be centrifuged at 16,100 x *g* at 4ºC for 30 min and supernatants stored at -80ºC. Protein concentrations will be determined using BCA assay (Pierce Biotechnology) and equal amounts of protein will be resolved on 4-12% gradient-Bis-Tris SDS-PAGE gels (Bio-Rad), transferred to PVDF membranes (Bio-Rad, Hercules, CA, USA) and blocked with 5% dry milk/BSA in 20 mM Tris base, 135 mM NaCl, 0.1% Tween 20 (TBST) and blotted against ubiquitinated substrates using a polyclonal antibody against ubiquitin (Cell Signaling Technology # 3933S). Membranes will be routinely washed using TBST, and incubated with a secondary anti-rabbit IgG horseradish peroxidase-conjugated antibody (Santa Cruz Biotechnology # sc-2077) followed by signal detection using enhanced chemi-luminescence reagent (Millipore). The signal will be quantitated using a Chemiluminescence imager and integrated signal quantification software from Bio-Rad.

An *in vitro* assay to evaluate clinical response to carfilzomib: The goal of this experimental approach is to develop a rapid *in vitro* assay to reflect the clinical response to carfilzomib treatment. In the present Phase I clinical trial, patients will receive cyclophosphamide (Hour 0-1) and etoposide (Hour 1-3) and then carfilzomib (Hour 3-3.5). Therefore, it is not possible to distinguish the clinical efficacy of carfilzomib alone from that of the other chemotherapeutic agents if cell viability of a patient's PBMCs are studied at or after Hour 3.

In the present assay in order to directly measure the cytotoxicity of carfilzomib, PBMCs, BM leukemia or infiltrating tumor cells and tumor cells isolated from solid tumors will be treated with different doses of carfilzomib and the IC50 will be measured using CellTiter-Glo® Luminescent Cell Viability Assay (Promega, Wisconsin, USA) in 384 well plates (Greiner). In this procedure 3,000 cells per well are seeded in 384-well plates in 40 µl RMPI complete medium. Cells will be cultured in a controlled atmosphere (37°C, 5% CO_2) for 24 hours. The following day the cells will be treated with carfilzomib diluted in an additional 10 µl RPMI complete medium per well. Cells will be incubated with carfilzomib for 72 h after which 25 µl of the CellTiter-Glo® Luminescent Cell Viability Reagent will be added per well and incubated at room temperature on a shaker for 30 min. The plate will be then read using an Envision plate reader (Perkin Elmer, Santa Clara, CA, USA). The results of this assay would reflect the clinical response to carfilzomib as monotherapy in tumor cells (or normal PBMCs when tumor cells not available) of the patient. Similar experiments will be set up to assess combinations with carfilzomib along with etoposide and cyclophosphamide (soluble metabolite) to compare *in vitro* sensitivity with *in vivo* clinical responses, a major goal of integrated genomic and personalized medicine.

Whole exome sequencing of leukemia and solid tumors along with germ line DNA as well as transcriptomic (RNA seq) analysis of leukemia and tumor cells to determine potential pathways contributing to carfilzomib drug sensitivity and/or resistance. This aim will also directly be used to generate an integrated genomic, RNA expression, and drug sensitivity model using a novel computer learning engine to predict responses to therapeutic agents: Ficoll-separated bone marrow mononuclear cells (BM MNCs which represent usually a significant enrichment of leukemic blasts) will be expanded in culture (please see appendix 1). One million cells will be pelleted and frozen at -80°C. DNA and RNA will be isolated from the BM MNCs and from the frozen solid tumor samples and processed, respectively, for Whole Exome Sequencing (WES) and for RNA-seq. DNA will also be isolated from a buccal swab sample for germ line analysis. Sequencing will be performed on the Illumina HiSeq 2500 platform at TGen. WES reads will be aligned using Bowtie to identify single-nucleotide mutations, multi-base pair insertions and deletions, and chromosomal amplifications and deletions. RNA sequences will be aligned using Cufflinks and TopHat to determine gene expression, to detect alternate splicing, and to identify translocations. Downstream analyses using Partek, Ingenuity, and BIONET will integrate DNA mutations and differential gene expression with pathway over-representation and drug-target identification.

10.2 STUDY ENDPOINTS

- Define MTD – see section 10.1.1
- Assess toxicities – see section 6.2
- Assess tumor response- see section 10.1.2
- Correlative studies – see section 10.1.3

10.3 SAMPLE SIZE CONSIDERATIONS

Sample size will be dependent upon dose escalations for each of the strata. Once the MTD or maximum dose level is reached, whichever comes first, an additional 6 patients will be enrolled in that strata for further toxicity analysis unless the Study Principal Investigator determines expansion cohort for that particular Strata should be discontinued due to subject safety or enrollment barriers.

10.4 INTERIM ANALYSIS

All study patients will be followed in real-time for toxicity and response. Each project will maintain a steering committee who will meet via conference call bi-weekly to critically review the scientific progress of the clinical trial. The steering committee will include the study principal investigators and research staff. The study principal investigator will also communicate directly with site investigators regarding their patients and any issues that arise. Trial progress, including discussion with all site principal investigators of each enrolled patient, will occur during a monthly conference call.

10.5 PLANNED METHODS OF ANALYSIS

Descriptive analysis will be performed regarding dose escalation, toxicities, tumor response, correlative studies.

10.6 DATA AND SAFETY MONITORING

The Data and Safety Monitoring Committee (DSMC) under the direction of Dr. Susan Slovin and Krista Napolitano, Manager, will be responsible for monitoring the data safety of the open and closed to accrual protocols sponsored by POETIC. The DSMC meets quarterly, and will review data quarterly, semi-annually or annually based on the risk level of the protocol. A copy of the MSKCC Data and Safety Monitoring Plans is on file at the Data and Coordinating Center.

The Data and Safety Monitoring (DSM) Plans at Memorial Sloan Kettering Cancer Center were initially created and approved by the National Cancer Institute in September 2001. The DSM Plans at MSKCC were established and are monitored by the Clinical Research Administration. The plans address the new policies set forth by the NCI in the document entitled "Policy of the

National Cancer Institute for Data and Safety Monitoring of Clinical Trials" which can be found at: http://cancertrials.nci.nih.gov/clinicaltrials/conducting/dsm-guidelines. The MSKCC Data and Safety Monitoring Plans can be found on the MSKCC Intranet at: http://mskweb2.mskcc.org/irb/index.htm. The Data and Safety Monitoring Committee (DSMC) monitors all Phase I and II clinical trials and reports to the Research Council and Institutional Review Board.

11 INVESTIGATIONAL PRODUCT - CARFILZOMIB

11.1 DESCRIPTION

Carfilzomib is a synthetic small molecule peptide bearing the chemical name (2S)-N-((S)-1-((S)-4-methyl-1-((R)-2-methyloxiran-2-yl)-1-oxopentan-2-ylcarbamoyl)-2-phenylethyl)-2-((S)-2-(2-morpholinoacetamido)-4-phenylbutanamido)-4-methylpentanamide. The molecular formula is $C_{40}H_{57}N_5O_7$ and the molecular weight is 719.91. It specifically functions as an inhibitor of the chymotrypsin-like activity of the 20S proteasome which leads to the accumulation of protein substrates within the cell and induction of apoptosis.

11.2 FORMULATION

Carfilzomib for Injection will be provided as a 60 mg lyophilized powder vial which, when reconstituted with 29 mL of Sterile Water for Injection, USP, contains 2 mg/mL isotonic solution of carfilzomib Free Base in 10 mM sodium citrate buffer (pH 3.5) containing 10% (w/v) sulfobutylether-□-cyclodextrin (SBE-□-CD, Captisol®).

To reconstitute, slowly direct the 29 mL sterile water into the inside of the vial to minimize foaming. Gently swirl and/or invert the vial slowly for about 1 minute, or until complete dissolution. DO NOT SHAKE to avoid foam generation. If foaming occurs, allow the solution to settle in the vial until foaming subsides (approximately 5 minutes) and the solution is clear. Visually inspect for particulate matter and discoloration prior to administration. The reconstituted product should be a clear, colorless solution and should not be administered if any discoloration or particulate matter is observed.

Withdraw the required dose of carfilzomib and dilute in 5% Dextrose Injection, USP according to the following table:

Dose-Based Carfilzomib Dilution

Carfilzomib Dose	Total Volume in 5% Dextrose Injection, USP
< 20mg	25mL
≥ 20 mg- < 50 mg	50mL
≥50 mg	100mL

Note that for the FIRST CYCLE ONLY, the total volume on Days 1 and 2 may differ from the volume of subsequent doses due to the different doses used during the step-up process.

11.3 DRUG SUPPLY

11.3.1 HOW DRUG IS SUPPLIED

Lyophilized Carfilzomib for Injection is an investigational therapeutic agent provided in a single-dose vial as a sterile, lyophilized powder in the following dosage:
60 mg Single-Use Glass Vial / 4 pk Carton

Each single-dose vial provides 60 mg of Carfilzomib in a 50 cc labeled glass vial with an elastomeric stopper and a flip-off lid. Flip-off lid colors may vary:
Green or Purple flip-off lid colors may be provided.

The product is supplied in labeled carton(s) containing four (4) single-use vials per carton and is shipped and stored between 2°C - 8°C (36°F - 46°F).

11.3.2 DRUG SUPPLY DISTRIBUTOR

Amgen is the contracted drug distribution company for the study.
All shipments are made via an expedited courier (typically overnight, but certain locations require 2-day shipping).

The initial Carfilzomib drug shipment will be initiated by Amgen upon site activation via the POETIC DCC.

All subsequent drug orders, Investigational Product (IP) requests will be requested electronically using the eZIP online drug order portal or manually requested using the Non-Amgen Sponsored Clinical Research (NASCR) Drug Shipment Request Form (see FORM-086838). Once properly completed, the NASCR Drug Shipment Request Form, if utilized, should sent via e-mail to to GCCS@amgen.com or faxed to +1 (805) 376-9807. The standard lead-time for IP requests is 2 weeks from the receipt of the Drug Shipment Request Form. Rush requests can be accommodated by communicating the urgency via email.

IP shipments will be shipped from Amgen Thousand Oaks to the sites using 2-8°C Credo shippers via UPS or FedEx. Credo is a re-usable shipping system and all shippers need to be returned to Amgen. Return process details are outlined in the Credo Shipper Return Instructions.

Temperature will be monitored through Amgen's shipper performance monitoring system, on an ad hoc basis. As such a temperature logger may be included in the shipment. Following receipt of a temperature logger, the site should follow instructions on the MTL Card Example form and the Proof of Receipt (POR), to report to Amgen.

To report any problems with drug supply or shipments, please email GCCS@amgen.com.

11.4 STORAGE

Lyophilized Carfilzomib for Injection must be stored at 2–8ºC (36°F - 46°F) in a securely locked area to which access is limited to appropriate study personnel. Retain vials in the original package to protect from light until use.

Stability of Carfilzomib

Storage Conditions of Reconstituted Carfilzomib	Stability[a] per Container		
	Vial	Syringe	IV Bag (D5W[b])
Refrigerated (2°C to 8°C; 36°F to 46°F)	24 hr	24 hr	24 hr
Room Temperature (15°C to 30°C; 59°F to 86°F)	4 hr	4 hr	4 hr

[a] Total time from reconstitution to administration should not exceed 24 hours

[b] 5% Dextrose Injection, USP

11.5 ACCOUNTABILITY

Amgen and the Site Principal Investigator will maintain records of each shipment of investigational product. The records will document shipment dates, method of shipment, batch numbers, and quantity of vials contained in the shipment. Upon receipt of the investigational product, the designated recipient at the study site will inspect the shipment, verify the number and condition of the vials, and prepare an inventory or drug accountability record.

Drug accountability records must be readily available for inspection by representatives of the POETIC DCC or designee, Amgen and by regulatory authorities.

Empty and partially used vials should be accounted for and destroyed at the study site in accordance with the internal standard operating procedures. Drug destruction records must be readily available for inspection by representatives of the POETIC DCC or designee, Amgen and by regulatory authorities.

Only sites that cannot destroy unused drug on-site will be required to return their unused supply of investigational product.

11.6 TOXICITY

Likely (>20%)	Less Likely (5-20%)	Rare But Serious (<5%)
• Fatigue (tiredness)<br>• Fever<br>• Headache<br>• Cough	• Anxiety<br>• Decrease white blood cell count, which may be with fever<br>• Confusion or changes in mental state	• Worsening liver function up to and including liver failure<br>• Cholestasis<br>• Multi-organ failure

• Shortness of breath (at rest or with exertion) which in rare cases may be life-threatening or resulting in death<br>• Nausea<br>• Vomiting<br>• Diarrhea<br>• Constipation<br>• Decreased red blood cell count which may lead to feeling tired<br>• Decreased platelet counts which may lead to increase bleeding or bruising<br>• Decreased white blood cell count which may decrease your ability to fight infection<br>• Respiratory tract infection<br>• Swelling of the hands, feet or ankles<br>• Back pain<br>• Stomach pain, discomfort, or swelling<br>• General weakness<br>• Chills<br>• Pneumonia<br>• Loss of or decreased appetite which may lead to weight loss<br>• Pain in the bones or joint pain<br>• Pain in limbs, hands or feet<br>• Muscle spasms<br>• Dizziness<br>• Insomnia (difficulty sleeping)<br>• Increase in blood pressure<br>• Changes to blood tests (decreased blood levels of potassium and/or magnesium, increased blood levels of sugar and/or creatinine)<br>• Infusion reactions (which can occur during or shortly after carfilzomib infusion) including flushing or feeling hot, fever, shakes, nausea, vomiting, weakness, shortness of breath, swelling of the face, pain in the muscles or	• Blurred or double vision<br>• Eye cataract<br>• Blood chemistry and electrolyte alterations<br>• Rash and/or itching, or dry skin<br>• Redness of the skin<br>• Pain, burning, or irritation at the infusion site<br>• Generalized pain<br>• Increased sweating<br>• Chest pain<br>• Heart failure*, and heart problems including rapid, strong or irregular heartbeat<br>• Blood clot in the lungs<br>• Fluid in the lungs<br>• Pain in the bones or joint pain<br>• Muscle spasm, pain, or weakness<br>• Indigestion (upset stomach)<br>• Inflammation of the liver (mild, reversible changes in liver function tests)<br>• Urinary tract infection<br>• Nosebleeds<br>• Dehydration<br>• Sore throat, inflammation of the nose and throat<br>• Change in voice or hoarseness<br>• Decrease in blood pressure<br>• Bronchitis<br>• Toothache<br>• Flu-like symptoms such as fever, chills, or shaking that may occur at any time but are more likely to occur on the day of or the day after carfilzomib infusion.<br>• Serious infection in the blood (sepsis)<br>• Viral infection<br>• Kidney problems, including decreased ability to make urine, increased creatinine in the blood, and kidney failure needing dialysis	• Decreased or worsening of heart function including chest pain, abnormal heart rhythm, heart attack.<br>• Bleeding, bruising, weakness, confusion, fever, nausea, vomiting and diarrhea, and acute kidney failure, which may be signs of a blood condition known as Thrombocytopenic Thrombotic Purpura/Haemolytic Uraemic Syndrome (TTP/HUS)<br>• Tumor lysis syndrome (TLS)<br>○ Tumor lysis syndrome is caused by rapid killing of tumor cells during treatment. When the tumor cells die, they release their contents into the bloodstream. If cell killing is very rapid, this can affect blood chemistries and the kidneys. In severe cases, this can lead to shutdown of kidney function requiring dialysis.<br>• Myelodysplastic syndromes (MDS)/ Acute Myeloid Leukemia (AML)<br>• Myelodysplastic syndromes refers to a disorder that develops when the cells in the bone marrow (the soft inner part of the bones, where new blood cells are made) do not work properly and have problems making new blood cells. A person with MDS may experience no symptoms or may experience fatigue, infection, easy bruising or bleeding. MDS can turn into a cancer of bone marrow cells called acute myeloid leukemia (AML).<br>• Posterior reversible encephalopathy syndrome (PRES) is a rare

joints, tightness or pain in the chest, and low blood pressure<br>• Numbness, tingling, or decreased sensation in hands and/or feet<br>• Runny nose or nasal congestion	• Changes to blood tests (decreased blood levels of sodium, protein, calcium or phosphate, increased blood levels of calcium, uric acid, potassium, or c-reactive protein)<br>• Blood clots in the veins<br>• Infusion site reaction (pain, redness or swelling where you received the injection into your vein)<br>• Wheezing<br>• Pulmonary hypertension	condition that causes swelling of the brain and affects how it functions. A person with PRES may experience headaches, confusion, loss or decreased level of consciousness, blurred vision or blindness, seizures, and possibly death. If caught early and treated, PRES may be reversed.<br>• Hypertension including hypertensive crises<br>• Pulmonary toxicities such as Interstitial Lung Disease (including pneumonitis), Acute Respiratory Failure and Acute Respiratory Distress Syndrome (ARDS)<br>• Gastrointestinal perforation<br>• Pericardial effusion<br>• Pericarditis<br>• Cerebrovascular accident<br>• Thrombotic microangiopathy<br>• Allergy to carfilzomib<br>• Laryngeal edema- swelling of the throat<br>• Cytomegalovirus chorioretinitis – infection of the back of the eye (cytomegalovirus)<br>• Progressive multifocal Leukoencephalopathy (PML), a viral infection that may cause clumsiness, trouble speaking, partial blindness and impaired brain function<br>• Hepatitis B Virus (HBV) reactivation, a serious liver infection that may come back if you have already had HBV<br>• Acute Pancreatitis-Inflammation of the pancreas gland<br>• Blockage of the intestines (bowel)

* There is an increased risk of heart failure in Asian-Pacific (China, Japan, Taiwan, Singapore, Republic of Korea, and Thailand) patients treated with carfilzomib

Cases of peritonitis have been reported from carfilzomib clinical studies and post marketing sources. A safety assessment on peritonitis in association with the use of carfilzomib could

not establish a causal association between the event and carfilzomib use. The event will continue to be monitored through routine pharmacovigilance activities.

Reproductive and development toxicity is an important potential risk for carfilzomib (see Appendix A of the Investigator's Brochure for details).

Another proteasome inhibitor (i.e. bortezomib) is known to produce transient decreases in CD4+ lymphocytes, which appear to play a major role in suppressing herpes zoster reactivation (Heider et al, 2010). However, it is not known whether carfilzomib has similar effects on herpes zoster reactivation to those observed with bortezomib. Most subjects included in studies with carfilzomib received antiviral prophylaxis and due to this fact it is not possible to calculate the true incidence of herpes zoster infection in subjects treated with carfilzomib. The risk of herpes zoster infections can be minimized by antiviral prophylaxis.

12 CYCLOPHOSPHAMIDE INJECTION (CYTOXAN) NSC #26271

12.1 DESCRIPTION

Cyclophosphamide is an alkylating agent related to nitrogen mustard. Cyclophosphamide is inactive until it is metabolized by P450 isoenzymes (CYP2B6, CYP2C9, and CYP3A4) in the liver to active compounds. The initial product is 4-hydroxycyclophosphamide (4-HC) which is in equilibrium with aldophosphamide which spontaneously releases acrolein to produce phosphoramide mustard. Phosphoramide mustard, which is an active bifunctional alkylating species, is 10 times more potent *in vitro* than is 4-HC and has been shown to produce interstrand DNA cross-link analogous to those produced by mechlorethamine. Approximately 70% of a dose of cyclophosphamide is excreted in the urine as the inactive carboxyphosphamide and 5-25% as unchanged drug. The plasma half-life ranges from 4.1 to 16 hours after IV administration.

12.2 FORMULATION:

Cyclophosphamide for injection is available as powder for injection or lyophilized powder for injection in 500 mg, 1 g, and 2 g vials. The powder for injection contains 82 mg sodium bicarbonate/100 mg cyclophosphamide and the lyophilized powder for injection contains 75 mg mannitol/100 mg cyclophosphamide.

If the drug will be administered as undiluted drug at the 20 mg/mL concentration, then reconstitute to 20 mg/mL with NS ONLY to avoid a hypotonic solution. If the drug will be further diluted prior to administration, then first reconstitute with NS, SWFI, or Bacteriostatic Water for Injection (paraben preserved only) to a concentration of 20 mg/mL. Following reconstitution further dilute in dextrose or saline containing solutions for IV use.

12. 3 DRUG SUPPLY:

This study will use commercial supply of cyclophosphamide which is available from various manufacturers.

12. 4 STORAGE:

Storage at or below 25ºC (77ºF) is recommended. The product will withstand brief exposures to temperatures up to 30ºC (86ºF).

12. 5 TOXICITY

	**Common**<br>Happens to 21-100 children out of every 100	**Occasional**<br>Happens to 5-20 children out of every 100	**Rare**<br>Happens to < 5 children out of every 100
**Immediate:**<br>Within 1-2 days of receiving drug	Anorexia, nausea & vomiting (acute and delayed)	Abdominal discomfort, Diarrhea	Transient blurred vision, nasal stuffiness with rapid administration, arrhythmias (rapid infusion), skin rash, anaphylaxis, SIADH
**Prompt:**<br>Within 2-3 weeks, prior to the next course	Leukopenia, alopecia, immune Suppression	Thrombocytopenia, anemia, hemorrhagic cystitis	Cardiac toxicity with high dose (acute – CHF hemorrhagic myocarditis, myocardial necrosis), hyperpigmentation, nail changes, impaired wound healing, infection secondary to immune suppression
**Delayed:**<br>Any time later during therapy	Gonadal dysfunction: azoospermia or oligospermia (prolonged or permanent)	Amenorrhea	Gonadal dysfunction: ovarian failure, interstitial pneumonitis, pulmonary fibrosis
**Late:**<br>Any time after completion of treatment			Secondary malignancy (ALL, ANLL, AML), bladder carcinoma (long term use > 2 years), bladder fibrosis
**Unknown Frequency and Timing**:	Fetal toxicities and teratogenic effects of cyclophosphamide (alone or in combination with other antineoplastic agents) have been noted in humans. Toxicities include: chromosomal abnormalities, multiple anomalies, pancytopenia, and low birth weight. Cyclophosphamide is excreted into breast milk. Cyclophosphamide is contraindicated during breast feeding because of reported cases of neutropenia in breast fed infants and the potential for serious adverse events		

12.1 ETOPOSIDE INJECTION (VEPESID®, ETOPOPHOS®, VP-16) NSC#141540

12.1.1 DESCRIPTION

A semisynthetic derivative of podophyllotoxin that forms a complex with topoisomerase II and DNA which results in single and double strand DNA breaks. Its main effect appears to be in the S and G_2 phase of the cell cycle. The initial $t_{½}$ is 1.5 hours and the mean terminal half-life is 4 to 11 hours. It is primarily excreted in the urine. In children, approximately 55% of the dose is excreted in the urine as etoposide in 24 hours. The mean renal clearance of etoposide is 7 to 10 mL/min/m² or about 35% of the total body clearance over a dose range of 80 to 600 mg/m². Etoposide, therefore, is cleared by both renal and non -renal processes, i.e., metabolism and biliary excretion. The effect of renal disease on plasma etoposide clearance is not known. Biliary excretion appears to be a minor route of etoposide elimination. Only 6% or less of an intravenous dose is recovered in the bile as etoposide. Metabolism accounts for most of the non-renal clearance of etoposide.

The maximum plasma concentration and area under the concentration time curve (AUC) exhibit a high degree of patient variability. Etoposide is highly bound to plasma proteins (~94%), primarily serum albumin. Pharmacodynamic studies have shown that etoposide systemic exposure is related to toxicity. Preliminary data suggests that systemic exposure for unbound etoposide correlates better than total (bound and unbound) etoposide. There is poor diffusion into the CSF < 5%.

12.1.2 FORMULATION:

Etoposide for Injection is available as a 20 mg/mL solution in sterile multiple dose vials (5 mL, 25 mL, or 50 mL each). The pH of the clear, nearly colorless to yellow liquid is 3 to 4. Each mL contains 20 mg etoposide, 2 mg citric acid, 30 mg benzyl alcohol, 80 mg modified polysorbate 80/tween 80, 650 mg polyethylene glycol 300, and 30.5 percent (v/v) alcohol. Vial headspace contains nitrogen.

Dilute etoposide to a final concentration ≤ 0.4 mg/mL in D5W or NS. Etoposide infusions are stable at room temperature for 96 hours when diluted to concentrations of 0.2 mg/mL; stability is 24 hours at room temperature with concentrations of 0.4 mg/mL. The time to precipitation is highly unpredictable at concentrations > 0.4 mg/mL. Use in-line filter during infusion secondary to the risk of precipitate formation. However, the use of an in-line filter is not mandatory since etoposide precipitation is unlikely at concentrations of 0.1-0.4 mg/mL. **Do not administer etoposide by rapid intravenous injection.** Slow rate of administration if hypotension occurs.

Leaching of diethylhexyl phthalate (DEHP) from polyvinyl chloride (PVC) bags occurred with etoposide0.4 mg/mL in NS. To avoid leaching, prepare the etoposide solution as close as possible, preferably within 4 hours, to the time of administration or alternatively as

per institutional policy; glass or polyethylene-lined (non-PVC) containers and polyethylene-lined tubing may be used to minimize exposure to DEHP.

12.1.3 DRUG SUPPLY:

This study will use commercial supply of etoposide which is available from various manufacturers.

12.1.4 STORAGE:

Unopened vials of etoposide are stable until expiration date on package at controlled room temperature (20˚-25˚C or 68°-77° F).

12.1.5 TOXICITY

	**Common**<br>Happens to 21-100 children out of every 100	**Occasional**<br>Happens to 5-20 children out of every 100	**Rare**<br>Happens to < 5 children out of every 100
**Immediate:**<br>Within 1-2 days of receiving drug	Nausea, vomiting	Anorexia	Transient hypotension during infusion; anaphylaxis (chills, fever, tachycardia, dyspnea, bronchospasm, hy-
**Prompt:**<br>Within 2-3 weeks, prior to next course	Myelosuppression (anemia, leukopenia), alopecia	Thrombocytopenia, diarrhea, abdominal pain, asthenia, malaise, rashes and urticaria	Peripheral neuropathy, mucositis, hepatotoxicity, chest pain, thrombophlebitis, congestive heart failure, Stevens-Johnson Syndrome, exfoliative dermatitis
**Delayed:**<br>Any time later during therapy			Dystonia, ovarian failure, amenorrhea, anovulatory cycles, hypomenorrhea, onycholysis of nails
**Late:**<br>Any time after completion of treatment			Secondary malignancy (preleukemic or leukemic syndromes)
**Unknown Frequency and Timing:**	Fetal toxicities and teratogenic effects of etoposide have been noted in animals at 1/20th of the human dose. It is unknown whether the drug is excreted in breast milk.		

12.2 CYTARABINE (CYTOSINE ARABANOSIDE, ARA C, CYTOSAR® NSC #63878)

12.2.1 DESCRIPTION

Cytarabine appears to act through the inhibition of DNA polymerase. A limited, but significant, incorporation of cytarabine into both DNA and RNA has also been reported. It exhibits cell phase specificity, primarily killing cells undergoing DNA synthesis (S-phase) and under certain conditions blocking the progression of cells from the G1 phase to the S-phase. Cytarabine is metabolized by deoxycytidine kinase and other nucleotide kinases to the nucleotide triphosphate (Ara-CTP), an effective inhibitor of DNA polymerase. Ara-CTP is inactivated by a pyrimidine nucleoside deaminase, which converts it to the nontoxic uracil derivative (Ara-U). It appears that the balance of kinase and deaminase levels may be an important factor in determining sensitivity or resistance of the cell to cytarabine. It has an initial distributive phase $t_{½}$ of about 10 minutes, with a secondary elimination phase $t_{½}$ of about 1 to 3 hours. Peak levels after intramuscular or subcutaneous administration of cytarabine occur about 20 to 60 minutes after injection and are lower than IV administration. Intrathecally administered doses are metabolized and eliminated more slowly with a $t_{½}$ of about 2 hours.

12.2.2 FORMULATION:

Cytarabine for Injection is available in vials of 100 mg, 500 mg, 1 g, and 2 g containing a sterile powder for reconstitution. It is also available at a 20 mg/mL concentration with benzyl alcohol (25 mL per vial) or as a preservative free solution (5 mL, 50 mL per vial), and at a 100 mg/mL concentration with benzyl alcohol (20 mL vial) or as preservative free solution (20 mL vial). Hydrochloric acid and/or sodium hydroxide may be added to adjust the pH.

***For **intrathecal** administration, dilute with 5-10 mL (or volume per institutional practice) **preservative free** 0.9% sodium chloride injection, lactated Ringer's injection, or Elliot's B solution. The volume of CSF removed should be equal to at least ½ the volume delivered.

Patient Age (years)	Recommended Volume	10% CSF volume	CSF Volume *
1 – 1.99	5 – 10 mL	5 mL	50 +/- 10 mL (babies)
2 – 2.99	5 – 10 mL	8 mL	80 +/- 20 mL (younger children)
3 – 8.99	5 – 10 mL	10 mL	100 +/- 20 mL (older children)
9 or greater	5 – 10 mL	13 mL	130+/- 30 mL (adults)

*Rieselbach, R.E. et.al. Subarachnoid distribution of drugs after lumbar injection; N Engl J Med. 1962 Dec 20; 267:1273-8

12.2.3 DRUG SUPPLY:

Commercially available from various manufacturers

12.2.4 STORAGE:

Store at 25°C (77°F); excursions permitted to 15°-30°C (59°- 86°F). Cytarabine solutions should be protected from light.

Intrathecal cytarabine mixed in NS, lactated Ringer's injection, or Elliot's B solution is stable for 24 Hours at 25ºC but contains no preservative and should be administered as soon as possible after preparation.

12.2.5 TOXICITY (Intrathecal):

	**Common**<br>Happens to 21-100 children out of every 100	**Occasional**<br>Happens to 5-20 children out of every 100	**Rare**<br>Happens to < 5 children out of every 100
**Immediate:**<br>Within 1-2 days of receiving drug	Nausea, vomiting, fever, headache	Arachnoiditis	Rash, somnolence, meningismus, convulsions, paresis
**Prompt:**<br>Within 2-3 weeks, prior to the next course			Myelosuppression, ataxia
**Delayed:**<br>Any time later during therapy, excluding the above condition			Necrotizing leukoencephalopathy, paraplegia, blindness (in combination with XRT & systemic therapy)

12.3 METHOTREXATE (MTX, AMETHOPTERIN, TREXALL®) NSC#000740

12.3.1 DESCRIPTION

A folate analogue which reversibly inhibits dihydrofolate reductase, the enzyme that reduces folic acid totetrahydrofolic acid. Inhibition of tetrahydrofolate formation limits the availability of one carbon fragments necessary for the synthesis of purines and the conversion of deoxyuridylate to thymidylate in the synthesis of DNA and cell reproduction. The polyglutamated metabolites of MTX also contribute to the cytotoxic effect of MTX on DNA repair and/or strand breaks. MTX cytotoxicity is highly dependent on the absolute drug concentration and the duration of drug exposure. MTX is actively transported across cell membranes. At serum methotrexate concentrations exceeding 0.1 µmol/mL, passive diffusion becomes a major means of intracellular transport of MTX. The drug is widely distributed throughout the body with the highest concentration in the kidney, liver, spleen, gallbladder and skin. Plasma concentrations following high dose IV MTX decline in a biphasic manner with an initial half-life of 1.5-3.5 hours, and a terminal half life of 8-15 hours. About 50% is bound to protein. After oral administration, approximately 60% of a 30 mg/m^2 dose is rapidly absorbed from the GI tract, with peak blood levels at 1 hour. At doses > 30 mg/m^2 absorption decreases significantly. Even at low doses absorption may be very erratic, varying between 23% and 95%. The elimination of MTX from the CSF after an intrathecal dose is characterized by a biphasic curve with half-lives of 4.5 and 14 hours. After intrathecal administration of 12 mg/m^2, the lumbar concentration of MTX is ~100 times higher than in plasma. (Ventricular concentration is ~ 10% of lumbar concentration). MTX is excreted primarily by the kidneys via glomerular filtration and active secretion into the proximal tubules. Renal clearance usually equals or exceeds creatinine clearance. Small amounts are excreted in the feces. There is significant entero-hepatic circulation of MTX. The distribution of MTX into third-space fluid collections, such as pleural effusions and ascitic fluid, can substantially alter MTX pharmacokinetics. The slow release of accumulated MTX from these third spaces over time prolongs the terminal half-life of the drug, leading to potentially increased clinical toxicity.

12.3.2 FORMULATION:

Methotrexate for Injection is available as a lyophilized powder for injection in 1000 mg vials. The powder for injection contains approximately 7 mEq sodium in the 1000 mg vial. Methotrexate for Injection is also available as a 25 mg/mL solution in 2, 4, 8, 10, and 40 mL preservative free vials and 2 and 10 mL vials with preservative. The 2, 4, 8, 10, and 40 mL solutions contain approximately 0.43, 0.86, 1.72, 2.15, and 8.6 mEq sodium per vial, respectively. The preserved vials contain 0.9% benzyl alcohol as a preservative.

***__For Intrathecal use__: Use **preservative free** 25 mg/mL solution. Dilute with 5-10 mL preservative free NS, lactated Ringer's, or Elliot's B solution as per institutional standard of practice. The volume of CSF removed should be equal to at least half the volume delivered.

*Rieselbach, R.E. et.al. Subarachnoid distribution of drugs after lumbar injection; N Engl J Med. 1962 Dec 20;

Patient Age (years)	Methotrexate dose	Recommended volume	10% CSF volume	CSF Volume *
1–1.99	8 mg	5–10 mL	5 mL	50 +/- 10 mL (babies)
2–2.99	10 mg	5-10 mL	8 mL	80 +/- 20 mL (younger children)
3–8.99	12 mg	5-10 mL	10 mL	100 +/- 20 mL (older children)
9 or greater	15 mg	5-10 mL	13 mL	130 +/- 30 mL (adults)

267:1273-8

12.3.3 DRUG SUPPLY:

Commercially available from various manufacturers.

12.3.4 STORAGE:

Sterile methotrexate powder or solution is stable at 20º-25ºC (68º-77ºF); excursions permitted to 15º-30ºC (59º- 86 Fº). Protect from light.

Diluted methotrexate for intrathecal administration is stable for 24 hours at 25ºC but contains no preservative and should be administered as soon as possible after preparation.

12.3.5 TOXICITY (Intrathecal):

	**Common**<br>Happens to 21-100 children out of every 100	**Occasional**<br>Happens to 5-20 children out of every 100	**Rare**<br>Happens to < 5 children out of every 100
**Immediate:**<br>Within 1-2 days of receiving drug	Nausea, headache	Arachnoiditis: (headache, fever, vomiting, meningismus, nuchal rigidity, and pleocytosis)	Anaphylaxis, vomiting, seizures(L), malaise, confusion, back pain, rash, bleeding into subarachnoid or subdural space (risk > with platelet counts < 20,000),
**Prompt:**<br>Within 2-3 weeks, prior to the next course			Myelosuppression, ataxia, somnolence, cranial nerve palsy, subacute myelopathy (paraparesis/paraplegia), speech disorders, pain in the legs, bladder dysfunction
**Delayed:**<br>Any time later during therapy, excluding the above condition		Cognitive disturbances (L)[1,] learning disability (L)[1]	Leukoencephalopathy[1] (L)
**Late:**<br>Any time after the completion of treatment			Progressive CNS deterioration[1]

[1] May be enhanced by HDMTX and/or cranial irradiation.
(L) Toxicity may also occur later.

13 REGULATORY OBLIGATIONS

13.1 INFORMED CONSENT

Before protocol-specified procedures are carried out, consenting professionals will explain full details of the protocol and study procedures as well as the risks involved to legal guardian/participants prior to their inclusion in the study. Participants/legal guardians will also be informed that they are free to withdraw from the study at any time. All participants/legal guardians (if patient is < 18 years old) must sign an IRB/PB-approved consent form indicating their consent to participate. This consent form meets the requirements of the Code of Federal Regulations and the Institutional Review Board/Privacy Board. The consent form will include the following:

1. The nature and objectives, potential risks and benefits of the intended study.
2. The length of study and the likely follow-up required.
3. Alternatives to the proposed study. (This will include available standard and investigational therapies. In addition, patients will be offered an option of supportive care for therapeutic studies.)
4. The name of the investigator(s) responsible for the protocol.
5. The right of the participant to accept or refuse study interventions/interactions and to withdraw from participation at any time.

If applicable, an assent form must be signed by patients <18 yo as per the regulations of each local institution's IRB.

Before any protocol-specific procedures can be carried out, the consenting professional will fully explain the aspects of patient privacy concerning research specific information. In addition to signing the IRB Informed Consent, all patients/legal guardians must agree to the Research Authorization component of the informed consent form.

Each participant/legal guardian and consenting professional will sign the consent form. The participant/legal guardian must receive a copy of the signed informed consent form.

13.2 COMPLIANCE WITH LAWS AND REGULATIONS

The study will be conducted in accordance with U.S. Food and Drug Administration (FDA) and International Conference on Harmonization (ICH) Guidelines for Good Clinical Practice (GCP), the Declaration of Helsinki, Health Canada, any applicable local health authority, and Institutional Review Board (IRB) or Ethics Committee requirements.

This study must have the approval of a properly constituted IRB or Ethics Committee. Before the investigational drug is shipped to the Investigator, the Investigator or designee will provide Amgen with a copy of the IRB or Ethics Committee approval letter stating that the study protocol and any subsequent amendments and informed consent form have been reviewed and approved.

The Site Investigator or designee will be responsible for obtaining annual IRB or Ethics Committee re-approval throughout the duration of the study. Copies of the Investigator's annual report to the IRB or Ethics Committee and copies of the IRB or Ethics Committee continuance of approval may be provided to Amgen as follows:

Amgen
Regulatory Department
One Amgen Center Drive
Thousand Oaks, CA 91320-1799

The Investigator is also responsible for notifying their IRB or Ethics Committee of any significant adverse events that are serious and/or unexpected.

Amgen will provide study sites with any expedited safety reports generated from any ongoing studies with carfilzomib, changes to the Investigator's Brochure, and any other safety information which changes the risk/benefit profile of carfilzomib during the conduct of the study, to allow him/her to fulfill his/her obligation for timely reporting to the IRB/ECs and other Investigators participating in the study.

Upon completion of the trial, the Investigator must provide the IRB or Ethics Committee and Amgen with a summary of the trial's outcome.

13.3 PRE-STUDY DOCUMENTATION REQUIREMENTS

Prior to implementing this protocol at the participating centers, approval must be obtained from the participating center's Local IRB of Record. The following documents must be provided to POETIC DCC before the participating site can be initiated and begin enrolling participants:

- Local IRB of Record approval(s) for the protocol, appendices, informed consent form, short form consent process, HIPAA authorization, Investigator's Brochures (IBs) and any other materials that will be handed to patients.
- Local IRB of Record membership
- Local IRB of Record's Federal Wide Assurance number and OHRP Registration number
- Curriculum vitae and medical license for each investigator and consenting professional
- Documentation of Human Subject Research Certification and HIPAA training for investigators and key staff members
- Documentation of Good Clinical Practice (GCP) training for the PI and co-PI at each participating site.
- Signed and dated FDA Related Forms 1572/1571 (if applicable)
- Lab Certifications and Reference Ranges for each lab listed on the 1572
- Signed Financial Disclosure forms (FDFs)
- Site contact list
- Delegation of Authority (DOA) / Delegation of Responsibility (DOR)
- Study Specific Training Documentation

- Signed Protocol Signature Page for Principal Investigator

Upon receipt of the required documents, the DCC will formally contact the site and grant permission to proceed with enrollment.

13.4 SUBJECT CONFIDENTIALITY

All institutional, FDA, and NCI requirements for human subjects must be met. This study will be carried out in compliance with the regulations of the Health Insurance Portability and Accountability Act (HIPAA). Each participating institution will have an appropriate assurance on file with the Office for Human Research Protection (OHRP), NIH. The Data and Coordinating Center is responsible for assuring that each participating institution has an OHRP assurance and must maintain copies of IRB approvals from each participating site. The Data and Coordinating Center is responsible for assuring that IRB approval has been obtained at each participating site prior to the first patient registration from that site.

Subject medical information obtained as part of this study is confidential, and must not be disclosed to third parties, except as noted below. The subject may request in writing that medical information be given to his/her personal physician.

The Investigator/Institution will permit direct access to source data and documents by the Data and Coordinating Center or designee, Amgen, its designee, the FDA and/or other applicable regulatory authority. The access may consist of trial-related monitoring, audits, IRB or Ethics Committee reviews, and FDA inspections.

Release of research results should preserve the privacy of medical information and must be carried out in accordance with Department of Health and Human Services Standards for Privacy of Individually Identifiable Health Information, 45 CFR 164.508.

14 ADMINISTRATIVE AND LEGAL OBLIGATIONS

14.1 PROTOCOL AMENDMENTS, IRB CORRESPONDENCE, AND STUDY TERMINATION

14.1.1 PROTOCOL AMENDMENTS/STATUS CHANGES

Each change to the protocol must be organized and documented by the POETIC DCC. After IRB approval at Stanford University, the POETIC DCC will distribute the amendment to the FDA and subsequently to the participating institutions . Participating sites must obtain approval for amendments from their IRB within 60 calendar days of receipt. If the amendment is the result of a safety issue or makes eligibility criteria more restrictive, sites will not be permitted to continue enrolling new participants until the participating site IRB approval has been granted. The participating sites will ensure that documentation for all IRB approved amendments are sent to the DCC and are maintained in the regulatory binder. This documentation will include the IRB approval letter referencing the protocol version date and amendment number, IRB approved protocol, IRB approved appendices and IRB approved consent forms.

The amendment will be written so that no other institution will need to reformat the information but can simply copy and distribute. An amendment memo as well as highlighted and clean copies of the protocol, appendices and consent forms will be distributed to the participating sites. The consent form will be a sample which may be edited in order to adhere to local IRB guidelines. The amendment number and version date will also be displayed on each amendment.

14.1.2 ADDITIONAL IRB CORRESPONDENCE

Continuing Review Approval

The Continuing Review Approval letter from the participating site's IRB and the most current approved version of the informed consent form should be submitted to the POETIC DCC within 7 days of expiration. Failure to submit the re-approval in the stated timeline will result in suspension of study activities.

Deviations

A protocol deviation is any change or departure from an IRB approved research protocol. Deviations may be either prospective or retrospective.

A prospective protocol deviation on this study is defined as a request to treat a research participant who does not meet all the eligibility criteria, pretreatment evaluation, or who requires alteration in their study plan. If a deviation from this protocol is proposed for a potential or existing participant enrolled on this study, written approval must be obtained from the study Principal Investigator and sent to the DCC prior to the action. All deviations need to be reported to the local site's IRB as per local institutional IRB policy.

A retrospective deviation is any change or departure from the research protocol that occurred without prior written approval from the study Principal Investigator.

For deviations that are identified after they occur, the participating site should report to the POETIC DCC as soon as possible.

Participating sites should report deviations and violations to their institution's IRB as soon as possible per that site's institutional guidelines. Approvals/acknowledgments from the participating site IRB for protocol deviations and violations should be submitted to POETIC DCC as received.

Other correspondence

Participating sites should submit other correspondence to their institution's IRB according to local guidelines, and submit copies of that correspondence to the POETIC DCC.

14.2 STUDY DOCUMENTATION AND ARCHIVE

Source documents must be provided to the DCC to ensure that real-time monitoring can be accomplished. Source documentation refers to original records of observations, clinical findings and evaluations that are subsequently recorded as data. Source documentation should be consistent with data entered into eCRFs, and should include a minimum of two identifiers to allow for data verification.

The DCC and participating site PI will maintain adequate and accurate records to enable the implementation of the protocol to be fully documented and the data to be subsequently verified.

The participating sites will ensure that all regulatory documents and participating site IRB correspondences are maintained in an onsite regulatory binder and sent to the DCC as outlined within the protocol. The DCC will also maintain a regulatory binder for each site; this binder may be paper or electronic.

Audits will be conducted by the DCC or designee for protocol and regulatory compliance, data verification and source documentation. Audits may be accomplished in one of two ways: (1) source documents and research records for selected patients are brought from participating sites to the DCC for audit, or (2) selected patient records may be audited on-site or via remote monitoring at participating sites per site institutional guidelines. The number of participants audited will be determined by available time and the complexity of the protocol. Each audit will be summarized and a final report will be sent to the PI at the audited participating site within 30 days of the audit.

These audits will be performed by persons who are qualified by training and experience to monitor the progress of the investigation. During these audits the following activities will take place:

- Review regulatory binders for protocol documentation;
- Ensure that case report forms are source data verified according to the monitoring plan
- Verify drug accountability is complete and accurate; and
- Verify compliance to GCPs, ICH guidelines, FDA regulations, and applicable SOPs.

If there is ever an audit at the DCC, then the DCC is responsible for having all source documents, research records, all IRB approval documents, Drug Accountability Record Forms, patient registration lists, response assessments scans, x-rays, etc. available for the audit.

14.3 STUDY MONITORING AND DATA COLLECTION

Clinical Research Staff at the POETIC DCC will be assigned to the study. The responsibilities of the POETIC DCC include project compliance, data collection, abstraction, data reporting, regulatory monitoring, problem solving and prioritization of study activities. The POETIC DCC will communicate with the Research Staff at the following member institutions: Alberta Children's Hospital, Memorial Sloan Kettering Cancer Center, Phoenix Children's Hospital, Lucile Packard Children's Hospital at Stanford, Dana Farber Cancer Institute, Arkansas Children's Hospital, Pennsylvania State University College of Medicine, University of Texas Health Science Center at San Antonio, Huntsman Cancer Institute and Arnold Palmer Hospital for Children.

Participating sites will enter data directly into the Medidata database and complete study-specific paper CRFs (if applicable). Source documentation must be sent to the DCC and should include a cover page listing relevant records enclosed per participant. The participating Site PI is responsible for ensuring these documents are completed accurately and legibly. The Site PI is also responsible for ensuring that all electronic CRFs and corresponding source documentation are completed and submitted within one week of the end of a treatment cycle.

The data collected for this study will be entered into a secure electronic database by the participating site staff. Data will be stored, monitored, and queried at an institutional level via the database and will be monitored and queried by the POETIC DCC staff or designee.

- Variables that will be recorded include the patient's birth date, date of diagnosis, date of study entry and histologic diagnosis.
- The results of the pretreatment and end of therapy evaluations, including the extent of disease evaluation (history, physical examination and imaging studies), baseline laboratory values, renal and hepatic function, as defined per protocol, will be recorded.
- All study related treatment data and concomitant drugs will be recorded.
- The presence of toxicity at baseline, during and for 30 days after administration of the investigational agent will be monitored and recorded.
- The results of the extent of disease evaluation (history, physical examination and imaging studies) following each course of treatment will be recorded.
- The patient's disease status and last follow-up will be recorded. If disease progresses or recurs, the results of the repeat extent of disease evaluation will be recorded.

Site research staff will be assigned at Alberta Children's Hospital, Memorial Sloan Kettering Cancer Center, Phoenix Children's Hospital, Lucile Packard Children's Hospital at Stanford, Dana Farber Cancer Institute, Arkansas Children's Hospital, Pennsylvania State University College of Medicine and theUniversity of Texas Health Science Center at San Antonio. Their responsibilities will include project compliance, data collection, abstraction and entry, data reporting, regulatory monitoring, problems and prioritization, maintaining file documentation of data for the clinical trial, pharmacokinetic or other biologic correlative study collection, and analysis as outlined for each patient enrolled on study. They will also be responsible for maintaining a regulatory binder for each protocol. The designated research staff will also be responsible for submitting required de-identified source documentation to the POETIC DCC via fax/secure email/electronic database or mail to the address below, within one week after the completion of each study cycle.

If a participating site is noncompliant with the protocol document, accrual privileges may be suspended until the outstanding issues have been resolved.

POETIC Data Coordinating Center
Pediatric Hematology/Oncology
Stanford University
455 Broadway Ave.
Redwood City, CA 94063
Phone: 650-736-0269
Fax: 650-736-3731
Email: poeticdcc@stanford.edu

15 REFERENCES

1. Demo SD, Kirk CJ, Aujay MA, Buchholz TJ, Dajee M, Ho MN, Jiang J, Laidig GJ, Lewis ER, Parlati F, Shenk KD, Smyth MS, Sun CM, Vallone MK, Woo TM, Molineaux CJ, Bennett MK. Anti-tumor activity of PR-171, a novel irreversible inhibitor of the proteasome. Cancer Res. 2007; 67(13):6383-91.

2. Arastu-Kapur S, Shenk K, Parlati F and Bennett M. Non-Proteasomal Targets of Proteasome Inhibitors Bortezomib and Carfilzomib. Blood (ASH Annual Meeting Abstracts), Nov 2008; 112: 2657.

3. Kirk CJ, Jiang J, Muchamuel T, Dajee M, Swinarski D, Aujay M, Bennett MK, Yang J, Lewis E, Laidig G and Molineaux CJ. The Selective Proteasome Inhibitor Carfilzomib Is Well Tolerated in Experimental Animals with Dose Intensive Administration. Blood (ASH Annual Meeting Abstracts), Nov 2008; 112: 2765.

4. Bortezomib Summary Basis of Approval (www.fda.gov/cder/foi/nda/2003/21602_Velcade.htm)

5. Bross PF, Kane R, Farrell AT, Abraham S, Benson K, Brower ME, Bradley S, Gobburu JV, Goheer A, Lee SL, Leighton J, Liang CY, Lostritto RT, McGuinn WD, Morse DE, Rahman A, Rosario LA, Verbois SL, Williams G, Wang YC, Pazdur R Approval summary for bortezomib for injection in the treatment of multiple myeloma. Clin Cancer Res. 2004 10(12 Pt 1):3954-64.

6. Kuhn DJ, Chen Q, Voorhees PM, Strader JS, Shenk, KD, Sun CM, Demo SD, Bennett, SD, van Leeuwen, F, Chanan-Khan, A and Orlowski, RZ. Potent activity of carfilzomib, a novel, irreversible inhibitor of the ubiquitin-proteasome pathway, against preclinical models of multiple myeloma. Blood. 2007 Nov 1;110(9): 3281-90.

7. O'Connor, O, Stewart AK, Vallone M, Molineaux CJ, Kunkel LA, Gerecitano JF, Orlowski RZ. A phase 1 dose escalation study of the safety and pharmacokinetics of the novel proteasome inhibitor carfilzomib (PR-171) in patients with hematologic malignancies. Clin Cancer Res. 2009; 15(22): 7085-7091.

8. Alsina M, Trudel S, Vallone M, Molineaux C, Kunkel L and Goy A. Phase 1 Single Agent Antitumor Activity of Twice Weekly Consecutive Day Dosing of the Proteasome Inhibitor Carfilzomib (PR-171) in Hematologic Malignancies. Blood (ASH Annual Meeting Abstracts), 2007; 110: 411.

9. Jagannath S, Vij R, Stewart K, Somlo G, Jakubowiak A, Trudel S, Schwartz R, Siegel D, Kunkel L, The Multiple Myeloma Research Consortium (MMRC). Final results of PX-171-003-A0, part 1 of an open-label, single-arm, phase II study of carfilzomib (CFZ) in patients (pts) with relapsed and refractory multiple myeloma (MM). J Clin Oncol. 2009 27:15s (suppl; abstr 8504).

10. Wang L, Siegel D, Kaufman JL, Stewart AK, Jakubowiak AJ, Alsina M, Kukreti V, Bahlis NJ, McDonagh KT, Belch A, Sebag M, Gabrail N, Le MH, Bennett MK, Kunkel L, Kauffman M, Orlowski RZ, Vij R, and The Multiple Myeloma Research Consortium (MMRC). Updated Results of Bortezomib-Naïve Patients in PX-171-004, An Ongoing Open-Label, Phase II Study of Single-Agent Carfilzomib (CFZ) in Patients with Relapsed or Refractory Myeloma (MM). Blood (ASH Annual Meeting Abstracts), Nov 2009; 114: 302.

11. Siegel D, Wang L, Orlowski RZ, Kaufman JL, Stewart AK, Kukreti V, Alsina M, Jakubowiak AJ, Jagannath D, McDonagh KT, Belch A, Bahlis NJ, Shustik C, Le MH, Kunkel L, Bennett MK, Kauffman M, Vij R, and The Multiple Myeloma Research Consortium (MMRC) PX-171-004, An Ongoing Open-Label, Phase II Study of Single-Agent Carfilzomib (CFZ) in Patients with Relapsed or Refractory Myeloma (MM); Updated Results From the Bortezomib-Treated Cohort.Blood (ASH Annual Meeting Abstracts), Nov 2009; 114: 303

12. Niesvizky R, Wang L, Orlowski RZ, Bensinger W, Alsina M, Gabrail N, Gutierrez A, Lori Kunkel, Michael Kauffman, and The Multiple Myeloma Research Consortium (MMRC) Phase Ib Multicenter Dose Escalation Study of Carfilzomib Plus Lenalidomide and Low Dose Dexamethasone (CRd) in Relapsed and Refractory Multiple Myeloma (MM). Blood (ASH Annual Meeting Abstracts), Nov 2009; 114: 304.

13. Badros AZ, Vij R, Martin T, Zonder JA, Woo T, Wang, Lee S, Wong A, and Niesvizky R. Phase I Study of Carfilzomib in Patients (Pts) with Relapsed and Refractory Multiple Myeloma (MM) and Varying Degrees of Renal Insufficiency. Blood (ASH Annual Meeting Abstracts), Nov 2009; 114: 3877.

14. Ruan Y, Liu D, Jayanthan A, Truong T, Boklan J, Narendran A. Cytotoxicity and target modulation in pediatric solid tumors by the proteasome inhibitor carfilzomib. AACR-NCI-EORTC International Conference on Molecular Targets and Cancer Therapeutics. Boston, MA, October 19-23 (2013).

15. Jayanthan A, Ruan Y, Hagerty M, Shah R, Truong T, Lewis VA, Boklan J, Trippett T, Gore L, Narendran A. In vitro growth inhibition, target modulation and drug synergy in pediatric leukemia by the novel proteasome inhibitor carfilzomib. American Society of Hematology Annual Meeting, New Orleans, LA, December 7-10 (2013).

16. O'Connor OA1, Smith EA, Toner LE, Teruya-Feldstein J, Frankel S, Rolfe M, Wei X, Liu S, Marcucci G, Chan KK, Chanan-Khan A. The combination of the proteasome inhibitor bortezomib and the bcl-2 antisense molecule oblimersen sensitizes human B-cell lymphomas to cyclophosphamide. Clin Cancer Res. 2006 May 1;12(9):2902-11.

17. Papadopoulos KP, Lee P, Singhal S, Holahan JR, Tolcher AW, Patnaik A, Vesole DH, Rosen S, Rosen PJ, Bilotti, Woo T, Lee S, Hannah AL, Siegel DS. PX-171-007: A phase 1b study evaluating the safety and efficacy of a 30-minute IV infusion of carfilzomib in patients with relapsed and/or refractory multiple myeloma. 16th Congress of European Hematology Association; June 9-12, 2011; London, UK.

18. Stewart AK, Rajkumar SV, Dimopoulos MA, et al. for the ASPIRE investigators. Carfilzomib, Lenalidomide, and Dexamethasone for Relapsed Multiple Myeloma. N Engl J Med. 2015; 372(2): 142-152.

19. Skolnik JM, Barrett JS, Jayaraman B, Patel D, Adamson PC. Shortening the timeline of pediatric phase I trials: the rolling six design. J Clin Oncol. 2008; 26:190-5.

20. Eisenhauer EA, Therasse P, Bogaerts J, Schwartz LH, Sargent D, Ford R, Dancey J, Arbuck S, Gwyther S, Mooney M, Rubinstein L, Shankar L, Dodd L, Kaplan R, Lacombe D, Verweij J. New response evaluation criteria in solid tumours: revised RECIST guideline (version 1.1). Eur J Cancer, 2009; 45(2):228-47.

21. Young H, Baum R, Cremerius U, Herholz K, Hoekstra O, Lammertsma AA, Pruim J, Price P. Measurement of clinical and subclinical tumour response using [18F]-fluorodeoxyglucose and positron emission tomography: review and 1999 EORTC recommendations. European Organization for Research and Treatment of Cancer (EORTC) PET Study Group. Eur J Cancer. 1999; 35:1773–1782.

22. Cheson BD, Pfistner B, Juweid ME, Gascoyne RD, Specht L, Horning SJ, Coiffier B, Fisher RI, Hagenbeek A, Zucca E, Rosen ST, Stroobants S, Lister TA, Hoppe RT, Dreyling M, Tobinai K, Vose JM, Connors JM, Federico M, Diehl V; International Harmonization Project on Lymphoma. Revised Response Criteria for Malignant Lymphoma. J Clin Oncol, 2007; 25: 579-586.

23. Jakob C1, Egerer K, Liebisch P, Türkmen S, Zavrski I, Kuckelkorn U, Heider U, Kaiser M, Fleissner C, Sterz J, Kleeberg L, Feist E, Burmester GR, Kloetzel PM, Sezer O. Circulating proteasome levels are an independent prognostic factor for survival in multiple myeloma. Blood. 2007 Mar 1;109 (5):2100-5.

24. Manasach EE, de Larrea CF, Zingone A, Korde N, Tageja N, Bhutani M, Kazandijian D, Wu P, Costello R, Burton D, Carter G, Zuchlinski D, Mulquin M, Maric I, Calvo K, Yuan C, Stetler-Stevenson M, Rocschewski M, Landgren O. Enzymatic activities of circulating plasma proteasomes (cProt) in newly diagnosed multiple myeloma (NDMM) patients undergoing treatment with carfilzomib, lenalidomide and dexamethasone (CRd). American Society of Hematology Annual Meeting, New Orleans, LA, December 7-10 (2013).

25. de Wilt LH1, Jansen G, Assaraf YG, van Meerloo J, Cloos J, Schimmer AD, Chan ET, Kirk CJ, Peters GJ, Kruyt FA. Proteasome-based mechanisms of intrinsic and acquired bortezomib resistance in non-small cell lung cancer. Biochem Pharmacol. 2012 Jan 15;83(2):207-17

APPENDIX A: NCI-CTCAE VERSION 4.03

Common Terminology Criteria for Adverse Events (CTCAE) of the
National Cancer Institute (NCI) v4.03

Publish Date: June 14, 2010

http://evs.nci.nih.gov/ftp1/CTCAE/CTCAE_4.03_2010-06-14_QuickReference_5x7.pdf

APPENDIX B: PERFORMANCE SCALE

Karnofsky		Lansky	
Score	Description	Score	Description
100	Normal, no complaints, no evidence of disease	100	Fully active, normal
90	Able to carry on normal activity, minor signs or symptoms of disease	90	Minor restrictions in physically strenuous activity
80	Normal activity with effort; some signs or symptoms of disease	80	Active, but tires more quickly
70	Cares for self, unable to carry on normal activity or do active work	70	Both greater restriction of and less time spent in play activity
60	Required occasional assistance, but is able to care for most of his/her needs	60	Up and around, but minimal active play; keeps busy with quieter activity
50	Requires considerable assistance and frequent medical care	50	Gets dressed, but lies around much of the day; no active play, able to participate in all quiet play and activities
40	Disabled, requires special care and assistance	40	Mostly in bed; participates in quiet activities
30	Severely disabled, hospitalization indicated. Death not imminent	30	In bed; needs assistance even for quiet play
20	Very sick, hospitalization indicated. Death not imminent	20	Often sleeping; play entirely limited to very passive activities
10	Moribund, fatal processes progressing rapidly	10	No play; does not get out of bed

APPENDIX C:

OPTIONAL SPECIMEN COLLECTION AND SHIPPING

Buccal Swab:

Buccal swab for genetic testing will be obtained at screening only.

Blood:

2 samples of 5 mL in Na Heparin to be drawn at each time point listed below. If patient is <10kg, 3mL of blood per sample is acceptable.

1 tube to be sent to Phoenix Children's Hospital Lab

1 tube to be sent to Dr. Narendran's lab

BLOOD CORRELATIVE STUDIES TIME POINTS	
**CYCLE 1:**	
Day 1	Hour 0 (pre-chemo)
Day 1	Hour 3 (between end of chemo and start of carfilzomib)
Day 2	Hour 0 (pre-chemo)
Day 3	Hour 0 (pre-chemo)
Day 8	If the patient is receiving carfilzomib on Day 8, then the sample should be drawn prior to administering the carfilzomib dose. If the patient is not receiving carfilzomib that day, then the sample can be drawn at any time.

CYCLE 2:	
Day 1	Hour 0 (pre-chemo)
Day 2	Hour 0 (pre-chemo)

Bone Marrow:

For patients with leukemia or solid tumors with bone marrow involvement, samples will be collected and sent at screening, and with each subsequent bone marrow aspirate. Each sample to consist of approximately 5 mL of bone marrow in Na Heparin

Tumor Sample:

For solid tumor patients, tumor tissue sample, if available, should be sent at screening and from any subsequent biopsies that are performed

OPTIONAL SPECIMEN REQUISITION–PHOENIX CHILDREN'S HOSPITAL LAB
BUCCAL SWAB

Buccal swab kits will be provided by the Phoenix Children's Hospital. Please follow instructions supplied in the kit.

- ☐ Samples should be sent ambient Monday – Thursday on the same day they are obtained.
- ☐ If a sample is collected on a Friday, or the day before a holiday, ship on the next business day.
- ☐ Include a copy of THIS requisition sheet in the shipping box. Maintain the original at the study site.
- ☐ Sample should be labeled with:
  - Patient ID #
  - Time point (Screening)
  - Date obtained

Patient ID:_____________

Sample: Buccal swab

Study time point: Screening

Date obtained:___/___/___

All samples should be shipped overnight to:

Phoenix Children's Hospital
Attn: Biorepository, 3rd Floor Main Lab
1919 E Thomas Road
Phoenix, AZ, 85016 USA
Tel: 602-933-1302 (lab)
602-933-0065 (office)
608-347-6136 (cell)

Please email the PCH Biorepository at PCHbiorepository@phoenixchildrens.com the business day prior to shipping to inform the lab personnel.

For further information on buccal specimens and directions for shipping, you may contact Matt Pancratz at mpancratz@phoenixchildrens.com.

OPTIONAL SPECIMEN REQUISITION–PHOENIX CHILDREN'S HOSPITAL LAB BLOOD

Make copies of this form as needed, and complete it as indicated. Maintain the original at the study site, and include a copy with the samples being shipped.

- ☐ Do **NOT** batch ship multiple days. Send each day's sample(s) on ice on the same day drawn.
- ☐ Samples should **ONLY** be shipped Monday – Thursday.
- ☐ If samples are drawn on a Friday or the day before a holiday, they need to be refrigerated at 4°C and shipped on the next business day.

All samples should consist of 5mL whole blood collected in a sodium heparin tube (if patient is <10kg, 3mL per sample is acceptable). Each sample must be labeled with the following:

- Patient ID #
- Time point (Cycle, Day, Hour)
- Date drawn
- Time drawn

PATIENT ID#____________

**CYCLE 1:**				
**Time Point**	**Date**	**Time**	**Date Shipped**	**Tracking #**
Day 1, Hour 0	___/___/___	____:____	___/___/___	
Day 1, Hour 3	___/___/___	____:____	___/___/___	
Day 2, Hour 0	___/___/___	____:____	___/___/___	
Day 3, Hour 0	___/___/___	____:____	___/___/___	
Day 8, Hour 0	___/___/___	____:____	___/___/___	

**CYCLE 2:**				
**Time Point**	**Date**	**Time**	**Date Shipped**	**Tracking #**
Day 1, Hour 0	___/___/___	____:____	___/___/___	
Day 2, Hour 0	___/___/___	____:____	___/___/___	

All samples should be packed with ice packs and shipped overnight to:

Phoenix Children's Hospital
Attn: Biorepository, 3rd Floor Main Lab
1919 E Thomas Road
Phoenix, AZ, 85016 USA

Tel: 602-933-1302 (lab)
602-933-0065 (office)
608-347-6136 (cell)

Please email the PCH Biorepository at PCHbiorepository@phoenixchildrens.com the business day prior to shipping to inform the lab personnel.

For further information on blood specimen collection and directions for shipping, you may contact Matt Pancratz at mpancratz@phoenixchildrens.com.

OPTIONAL SPECIMEN REQUISITION–PHOENIX CHILDREN'S HOSPITAL LAB
BONE MARROW

Make copies of this form as needed, and complete it as indicated. Maintain the original at the study site, and include a copy with the samples being shipped.

- ☐ Do **NOT** batch ship multiple days. Send each day's sample on ice on the same day drawn.
- ☐ Samples should **ONLY** be shipped Monday – Thursday.
- ☐ If samples are drawn on a Friday or the day before a holiday, they need to be refrigerated at 4˚C and shipped on the next business day.

5mL of bone marrow should be collected in a sodium heparin tube. The tube must be labeled with the following:

- Patient ID #
- Time point (Cycle, Day, Hour)
- Date obtained
- Time obtained

PATIENT ID#____________

BONE MARROW				
**Time Point**	**Date**	**Time**	**Date Shipped**	**Tracking #**
Screening	___/___/___	____:____	___/___/___	
Cycle___, Day____	___/___/___	____:____	___/___/___	

All samples should be packed with ice packs and shipped overnight to:

Phoenix Children's Hospital
Attn: Biorepository, 3rd Floor Main Lab
1919 E Thomas Road
Phoenix, AZ, 85016 USA

Tel: 602-933-1302 (lab)
602-933-0065 (office)
608-347-6136 (cell)

Please email the PCH Biorepository at PCHbiorepository@phoenixchildrens.com the business day prior to shipping to inform the lab personnel.

For further information on bone marrow specimens and directions for shipping, you may contact Matt Pancratz at mpancratz@phoenixchildrens.com.

OPTIONAL SPECIMEN REQUISITION–PHOENIX CHILDREN'S HOSPITAL LAB TUMOR SAMPLES

Make copies of this form as needed, and complete it as indicated. Maintain the original at the study site, and include a copy with the samples being shipped.

☐ Samples should **ONLY** be shipped Monday – Thursday.

Snap frozen tissue for DNA/RNA extraction: at least 20 mg (wet weight) of tumor tissue that is representative of the malignancy (0.25 g is approximately 1.1 x 0.9 x 0.5 cm).

Each tube must be labeled with the following:
- Patient ID #
- Time point (Cycle, Day)
- Date obtained
- Time obtained

PATIENT ID#____________

PATIENT'S DISEASE__________________________________

TUMOR SAMPLE			
**Time Point**	**Date Obtained**	**Date Shipped**	**Tracking #**
Screening	___/___/___	___/___/___	
**If subsequent samples obtained**			
	___/___/___	___/___/___	
	___/___/___	___/___/___	

All samples should be shipped overnight to:

Phoenix Children's Hospital
Attn: Biorepository, 3rd Floor Main Lab
1919 E Thomas Road
Phoenix, AZ, 85016 USA

Tel: 602-933-1302 (lab)
602-933-0065 (office)
608-347-6136 (cell)

Please email the PCH Biorepository at PCHbiorepository@phoenixchildrens.com the business day prior to shipping to inform the lab personnel.

For further information on acceptable tumor specimens and directions for tumor shipping, you may contact Matt Pancratz at mpancratz@phoenixchildrens.com.

OPTIONAL SPECIMEN REQUISITION – DR. ARU NARENDRAN
BLOOD

Make copies of this form as needed, and complete it as indicated. Maintain the original at the study site, and include a copy with the samples being shipped.

- ☐ Include the **Canada Customs Information sheet** (next page) in the address pouch of the package.
- ☐ Do **NOT** batch ship multiple days. Send each day's sample(s) on ice on the same day drawn.
- ☐ Samples should **ONLY** be shipped Monday – Thursday.
- ☐ If samples are drawn on a Friday or the day before a holiday, they need to be refrigerated at 4˚C and shipped on the next business day.

All samples should consist of 5mL whole blood collected in a sodium heparin tube (if patient is <10kg, 3mL per sample is acceptable). Each sample must be labeled with the following:

- Patient ID #
- Time point (Cycle, Day, Hour)
- Date drawn
- Time drawn

PATIENT ID#____________

**CYCLE 1:**				
**Time Point**	**Date**	**Time**	**Date Shipped**	**Tracking #**
Day 1, Hour 0	___/___/___	____:____	___/___/___	
Day 1, Hour 3	___/___/___	____:____	___/___/___	
Day 2, Hour 0	___/___/___	____:____	___/___/___	
Day 3, Hour 0	___/___/___	____:____	___/___/___	
Day 8, Hour 0	___/___/___	____:____	___/___/___	

**CYCLE 2:**				
**Time Point**	**Date**	**Time**	**Date Shipped**	**Tracking #**
Day 1, Hour 0	___/___/___	____:____	___/___/___	
Day 2, Hour 0	___/___/___	____:____	___/___/___	

SPECIMEN REQUISITION – DR. ARU NARENDRAN (continued)

All samples should be packed with ice packs, include this form inside the shipping box, Canada custom form in the address pouch on the outside of the box, and ship overnight to:

Dr. Aru Narendran
University of Calgary
Laboratory HM336 AN
2500 University Drive NW
Calgary AB T2N1N4 Canada
Tel: (403) 210-6402 or 403-210-6418

For: Canada Customs Information

Date:

Description: Cells for research
Research reagent only
Origin: USA
Not known to be infectious
Not radioactive
No commercial value.

Transport/ Packaging: ICE Pack

To: Dr. Aru Narendran
Associate Professor of Oncology and Pediatrics
University of Calgary
Laboratory HM336 AN
2500 University Drive NW
Calgary AB T2N1N4 Canada
(403) 210-6402
Or Alberta Children's Hospital (403) 955-7211 pager 2700

From:

APPENDIX D: Roadmaps

Phase I study of carfilzomib in combination with cyclophosphamide and etoposide for children with relapsed and refractory solid tumors and leukemias

Patient Label

Cycle:___1__Dose Level:______ (assigned at enrollment)
The dosing schedule for this study will consist of a 28-day cycle.
See section 3.12 and 4.1 – 4.3 for laboratory criteria required to begin each treatment cycle.

Leukemia: First Cycle
(Carfilzomib dosing Days 1 – 5)

Drug	Route & Time	Dose		Days	Important Notes	Observations
IT Chemotherapy<br>ALL: IT Methotrexate (MTX)<br>AML or MPAL: IT Cytarabine	Intrathecal	**IT Methotrexate**<br>0.5-0.99 yo: 6 mg<br>1-1.99 yo : 8 mg<br>2-2.99 yo : 10 mg<br>3-8.99 yo : 12 mg<br>≥9 yo: 15 mg	**IT Cytarabine**<br>0.5-0.99 yo: 20 mg<br>1-1.99 yo: 30 mg<br>2-2.99 yo: 50 mg<br>> 3 yo:70 mg	Up to 14 days prior to IV chemo start	Patient must be documented CNS1 or CNS2 prior to study enrollment.<br>Intrathecal chemotherapy can be given during diagnostic lumbar puncture.	**A: Physical exam**<br>**B: Ht, Wt, BSA**<br>**C: CBC w/differential**<br>**D: Blood chemistry**<br>**E: Urinalysis**<br>**F: PT/PTT/Fibrinogen**[1]<br>**G: Vital signs w/ pulse oximetry**<br>**H: Serum pregnancy**[2]<br>**I: Bone marrow aspirate**[3]<br>**J: CSF**<br>**K: EKG & ECHO**<br>**L: Radiologic exam**[4]<br>**M: Correlative studies-blood**<br>[1] Repeat weekly if abnormal until normalized<br>[2] For females of childbearing potential<br>[3]If bone marrow aspirate unattainable, bone marrow biopsy may be substituted<br>[4]Appropriate radiologic exam for patients with extramedullary involvement
Pre- and post-hydration required: See section 6.1 for details.						
Dexamethasone 0.1mg/kg (max 4mg) IV/PO pre-medication required. See section 6.1 and 6.4 for details.						
Cyclophosphamide (CPM)	IV<br>over 60 minutes	______mg/m2 or _______mg/kg if <10kg		1 - 5	Administer Hour 0 – 1<br>If <10kg, see section 3.12 of protocol for dosing	
Etoposide (ETOP)	IV<br>over 120 minutes	______mg/m2 or _______mg/kg if <10kg		1 - 5	Administer Hour 1 – 3<br>If <10kg, see section 3.12 of protocol for dosing	
Carfilzomib	IV<br>over 30 minutes	_______mg/m2 or _______mg/kg if <10kg		1 - 5	Administer Hour 3 – 3.5<br>If <10kg, see section 3.12 of protocol for dosing	
G-CSF<br>or<br>PEG G-CSF	Subcutaneous injection	G-CSF: 5mcg/kg daily<br>or<br>PEG G-CSF: 0.1mg/kg (max 6mg) x1 dose		Start on Day 6	For daily G-CSF: administer until ANC is at least ≥1000 post nadir	

Therapy Delivery Map: **Ht:_______cm Wt:________kg BSA:________m²**

Protocol Day	Date Due	Date Given	IT MTX or IT Cytarabine _______mg	CPM _______mg	ETOP _______mg	Carfilzomib _______mg	☐ G-CSF ______mcg or ☐ PEG G-CSF _____mg	Observations
**–13 to 0**			**_______mg**					**J**
**Screening**	**Procedures to be completed within 7 days prior to enrollment, with the exception of EKG, ECHO, bone marrow aspirate/biopsy, and any radiologic disease evaluations, which can be performed within 14 days prior to enrollment.**							**A, B, C, D, E, F, G, H, I, K, L**
**1***				______mg	______mg	______mg		**A, B, C, D, E, G, M**
**2**				______mg	______mg	______mg		**A, C, D, E, G, M**
**3**				______mg	______mg	______mg		**A, C, D, E, G, M**
**4**				______mg	______mg	______mg		**A, C, D, E, G**
**5**				______mg	______mg	______mg		**A, C, D, E, G**
**6**							_____mcg or _____mg	
**8**							↓	**A, C, D, F, G, M**
**15**							↓	**A, C, D, F, G**
**22**							↓	**A, C, D, F, G**
**28**							**Date of last dose of G-CSF:___/___/___**	**A, C, D, F, G, I, L**

***IV chemotherapy should begin within 72 hours of study enrollment.**

Phase I study of carfilzomib in combination with cyclophosphamide and etoposide for children with relapsed and refractory solid tumors and leukemias

Patient Label

Cycle:_______Dose Level:________ (assigned at enrollment)
The dosing schedule for this study will consist of a 28-day cycle.
See section 3.12 and 4.1 – 4.3 for laboratory criteria required to begin each treatment cycle.

<u>Leukemia:</u> Subsequent Cycles
(Carfilzomib dosing Days 1 – 5)

Drug	Route & Time	Dose		Days	Important Notes
IT Chemotherapy<br>ALL: IT Methotrexate (MTX)<br>AML or MPAL: IT Cytarabine	Intrathecal	**IT Methotrexate**<br>0.5-0.99 yo: 6 mg<br>1-1.99 yo : 8 mg<br>2-2.99 yo : 10 mg<br>3-8.99 yo : 12 mg<br>≥9 yo: 15 mg	**IT Cytarabine**<br>0.5-0.99 yo: 20 mg<br>1-1.99 yo: 30 mg<br>2-2.99 yo: 50 mg<br>> 3 yo: 70 mg	1	Patient must be documented CNS1 or CNS2 prior to continuing on study treatment. Intrathecal chemotherapy can be given with bone marrow aspirate at end of previous cycle to avoid second anesthesia.
Pre- and post-hydration required: See section 6.1 for details.					
Dexamethasone 0.1mg/kg (max 4mg) IV/PO pre-medication may be required for certain patients. See section 6.1 and 6.4 for details.					
Cyclophosphamide (CPM)	IV<br>over 60 minutes	______mg/m2 or ______mg/kg if <10kg		1 - 5	Administer Hour 0 – 1<br>If <10kg, see section 3.12 of protocol for dosing
Etoposide (ETOP)	IV<br>over 120 minutes	______mg/m2 or ______mg/kg if <10kg		1 - 5	Administer Hour 1 – 3<br>If <10kg, see section 3.12 of protocol for dosing
Carfilzomib	IV<br>over 30 minutes	______mg/m2 or ______mg/kg if <10kg		1 - 5	Administer Hour 3 – 3.5<br>If <10kg, see section 3.12 of protocol for dosing
G-CSF<br>or<br>PEG G-CSF	Subcutaneous injection	G-CSF: 5mcg/kg daily<br>or<br>PEG G-CSF: 0.1mg/kg (max 6mg) x1 dose		Start on Day 6	For daily G-CSF: administer until ANC is at least ≥1000 post nadir

Observations

A: Physical
B: Ht, Wt, BSA
C: CBC w/differential
D: Blood chemistry
E: Urinalysis
F: Vital signs w/ pulse oximetry
G: Serum pregnancy[1]
H: Bone marrow aspirate[2, 3]
I: CSF[3]
J: EKG & ECHO
K: Radiologic exam[4]
L: Correlative Studies-blood[5]

[1] For females of childbearing potential
[2] If bone marrow aspirate unattainable, bone marrow biopsy may be substituted
[3] Bone marrow and CSF may be done at same time to avoid second anesthesia
[4] Appropriate radiologic exam for patients with extramedullary involvement
[5] Cycle 2 only

Therapy Delivery Map **Ht:_______cm** **Wt:________kg** **BSA:________m^2**

Protocol Day	Date Due	Date Given	IT MTX or IT Cytarabine _______mg	CPM _______mg	ETOP _______mg	Carfilzomib _______mg	☐ G-CSF ________mcg or ☐ PEG G-CSF _______mg	Observations
**Date of IT chemo**			________mg					**H, I**
**1****				______mg	______mg	______mg		**A, B, C, D, E, F, G, L**
**2**				______mg	______mg	______mg		**F, L**
**3**				______mg	______mg	______mg		**F**
**4**				______mg	______mg	______mg		**F**
**5**				______mg	______mg	______mg		**F**
**6**							_____mcg or _____mg	
**28**							**Date of last dose of G-CSF:___/___/___**	**A, C, D, F, K**

****Observations do not need to be repeated on Day 1 of the new cycle if they were completed within 72 hours of Day 1, unless otherwise specified by the protocol****

Phase I study of carfilzomib in combination with cyclophosphamide and etoposide for children with relapsed and refractory solid tumors and leukemias

Patient Label

Cycle:___1___ Dose level:________ (assigned at enrollment)

Solid Tumors and Lymphomas: First cycle

The dosing schedule for this study will consist of a 28-day cycle. **(Carfilzomib dosing Days 1 – 5)**

See section 3.12 and 4.1 – 4.3 for hematologic/serum chemistries required to begin each treatment cycle.

Drug	Route & Time	Dose	Days	Important Notes
Methotrexate (MTX)	Intrathecal	**IT Methotrexate**<br>0.5-0.99 yo: 6 mg<br>1-1.99 yo : 8 mg<br>2-2.99 yo : 10 mg<br>3-8.99 yo : 12 mg<br>≥9 yo: 15 mg	1	**FOR NON-HODGKIN'S LYMPHOMA PATIENTS ONLY**<br>Intrathecal chemotherapy can be given during diagnostic lumbar puncture<br>Can be done with bone marrow, if indicated, to avoid second anesthesia
Pre- and post-hydration required: See section 6.1 for details.				
Dexamethasone 0.1mg/kg (max 4mg) IV/PO pre-medication required. See section 6.1 and 6.4 for details.				
Cyclophosphamide (CPM)	IV<br>over 60 minutes	______mg/m2 or _______mg/kg if <10kg	1 - 5	Administer Hour 0 – 1<br>If <10kg, see section 3.12 of protocol for dosing
Etoposide (ETOP)	IV<br>over 120 minutes	______mg/m2 or _______mg/kg if <10kg	1 - 5	Administer Hour 1 – 3<br>If <10kg, see section 3.12 of protocol for dosing
Carfilzomib	IV<br>over 30 minutes	_______mg/m2 or _______mg/kg if <10kg	1 - 5	Administer Hour 3 – 3.5<br>If <10kg, see section 3.12 of protocol for dosing
G-CSF<br>Or<br>PEG G-CSF	Subcutaneous injection	G-CSF: 5mcg/kg<br>Or<br>PEG G-CSF: 0.1mg/kg, max 6mg	Start on Day 6	Administer G-CSF until ANC at least 1,000 post nadir;<br>Or<br>Administer PEG G-CSF x1 dose

Observations

A: Physical exam
B: Ht, Wt, BSA
C: CBC w/differential
D: Blood chemistry
E: Urinalysis
F: PT/PTT/Fibrinogen[1]
G: Vital signs w/ pulse oximetry
H: Serum pregnancy[2]
I: Tumor assessment
J: CSF[3]
K: EKG & ECHO
L: Correlative studies-blood

[1] Repeat weekly if abnormal until normalized
[2] For females of childbearing potential
[3] <u>Non-Hodgkin's lymphoma patients ONLY</u>

Therapy Delivery Map **Ht:_______cm Wt:________kg BSA:________m^2**

Protocol Day	Date Due	Date Given	IT MTX _____mg (For Non-Hodgkin's lymphoma ONLY)	CPM _____mg	ETOP _____mg	Carfilzomib _____mg	☐ G-CSF _____mcg or ☐ PEG G-CSF _____mg	Observations
– 13 to 0			______mg					J
Screening	Procedures to be completed within 7 days prior to enrollment, with the exception of EKG, ECHO, and tumor assessment, which can be performed within 14 days prior to enrollment.							A, B, C, D, E, F, G, H, I, K
1*				______mg	______mg	______mg		A, B, C, D, E, G, L
2				______mg	______mg	______mg		A, C, D, E, G, L
3				______mg	______mg	______mg		A, C, D, E, G, L
4				______mg	______mg	______mg		A, C, D, E, G
5				______mg	______mg	______mg		A, C, D, E, G
6							_____mcg OR _____mg	
8							↓	A, C, D, F, G, L
15							↓	A, C, D, F, G
22							↓	A, C, D, F, G
28							Date of last dose of G-CSF:___/___/___	A, C, D, F, G

*** IV chemotherapy should begin within 72 hours of study enrollment.**

Patient Label

Cycle:_______ Dose level:________ (assigned at enrollment)

Solid Tumors and Lymphomas: Subsequent cycles

The dosing schedule for this study will consist of a 28-day cycle. **(Carfilzomib dosing Days 1 – 5)**

See section 3.12 and 4.1 – 4.3 for hematologic/serum chemistries required to begin each treatment cycle.

Drug	Route & Time	Dose	Days	Important Notes
Methotrexate (MTX)	Intrathecal	**IT Methotrexate**<br>0.5-0.99 yo: 6 mg<br>1-1.99 yo : 8 mg<br>2-2.99 yo : 10 mg<br>3-8.99 yo : 12 mg<br>≥9 yo: 15 mg	1	**FOR NON-HODGKIN'S LYMPHOMA PATIENTS ONLY**<br>Intrathecal chemotherapy can be given during response assessment lumbar puncture<br>Can be done with bone marrow, if indicated, to avoid second anesthesia
Pre- and post-hydration required: See section 6.1 for details.				
Dexamethasone 0.1mg/kg (max 4mg) IV/PO pre-medication may be required for certain patients. See section 6.1 and 6.4 for details.				
Cyclophosphamide (CPM)	IV<br>over 60 minutes	______mg/m2 or _______mg/kg if <10kg	1 - 5	Administer Hour 0 – 1<br>If <10kg, see section 3.12 of protocol for dosing
Etoposide (ETOP)	IV<br>over 120 minutes	______mg/m2 or _______mg/kg if <10kg	1 - 5	Administer Hour 1 – 3<br>If <10kg, see section 3.12 of protocol for dosing
Carfilzomib	IV<br>over 30 minutes	_______mg/m2 or _______mg/kg if <10kg	1 - 5	Administer Hour 3 – 3.5<br>If <10kg, see section 3.12 of protocol for dosing
G-CSF<br>Or<br>PEG G-CSF	Subcutaneous injection	G-CSF: 5mcg/kg<br>or<br>PEG G-CSF: 0.1mg/kg, max 6mg	Start on Day 6	Administer G-CSF until ANC at least 1,000 post nadir;<br>or<br>Administer PEG G-CSF x1 dose

Observations

A: Physical exam
B: Ht, Wt, BSA
C: CBC w/differential
D: Blood chemistry
E: Urinalysis
F: PT/PTT/Fibrinogen[1]
G: Vital signs w/ pulse oximetry
H: Serum pregnancy[2]
I: Tumor assessment[3]
J: CSF[4]
K: EKG & ECHO
L: Correlative studies-blood[5]

[1] Repeat weekly if abnormal until normalized
[2] For females of childbearing potential
[3] Following cycles 2, 4, 6, then every 3 cycles unless otherwise clinically indicated; Include bone marrow evaluation if tumor with bone marrow involvement at the time of study enrollment.
[4] **Non-Hodgkin's lymphoma patients ONLY**
[5] Cycle 2 only

Therapy Delivery Map **Ht:________cm** **Wt:_______kg** **BSA:_______m2**

Protocol Day	Date Due	Date Given	IT MTX _____mg (for Non-Hodgkin's lymphoma ONLY)	CPM _____mg	ETOP _____mg	Carfilzomib _____mg	☐ G-CSF _____mcg or ☐ PEG G-CSF _____mg	Observations
**Date of IT chemo**			______mg					J
1**				______mg	______mg	______mg		A, B, C, D, E, G, H, L
2				______mg	______mg	______mg		G, L
3				______mg	______mg	______mg		G
4				______mg	______mg	______mg		G
5				______mg	______mg	______mg		G
6							_____mcg OR _____mg	
28							Date of last dose of G-CSF:___/___/___	A, C, D, G, I

****Observations do not need to be repeated on Day 1 of the new cycle if they were completed within 72 hours of Day 1, unless otherwise specified by the protocol****

Patient Label

Cycle:___1__Dose Level:______ (assigned at enrollment)
The dosing schedule for this study will consist of a 28-day cycle.

Leukemia: First Cycle
(Carfilzomib twice weekly dosing)

See section 3.12 and 4.1 – 4.3 for laboratory criteria required to begin each treatment cycle.

Drug	Route & Time	Dose		Days	Important Notes
IT Chemotherapy<br>ALL: IT Methotrexate (MTX)<br>AML or MPAL: IT Cytarabine	Intrathecal	**IT Methotrexate**<br>0.5-0.99 yo: 6 mg<br>1-1.99 yo : 8 mg<br>2-2.99 yo : 10 mg<br>3-8.99 yo : 12 mg<br>≥9 yo: 15 mg	**IT Cytarabine**<br>0.5-0.99 yo: 20 mg<br>1-1.99 yo: 30 mg<br>2-2.99 yo: 50 mg<br>> 3 yo: 70 mg	Up to 14 days prior to IV chemo start	Patient must be documented CNS1 or CNS2 prior to study enrollment.<br>Intrathecal chemotherapy can be given during diagnostic lumbar puncture.
Pre- and post-hydration required: See section 6.1 for details.					
Dexamethasone 0.1mg/kg (max 4mg) IV/PO pre-medication required. See section 6.1 and 6.4 for details.					
Cyclophosphamide (CPM)	IV<br>over 60 minutes	______mg/m2 or _______mg/kg if <10kg		1 - 5	Administer Hour 0 – 1<br>If <10kg, see section 3.12 of protocol for dosing
Etoposide (ETOP)	IV<br>over 120 minutes	______mg/m2 or _______mg/kg if <10kg		1 - 5	Administer Hour 1 – 3<br>If <10kg, see section 3.12 of protocol for dosing
Carfilzomib	IV<br>over 30 minutes	_______mg/m2 or _______mg/kg if <10kg		1 – 2<br>8 – 9<br>15 – 16	Administer Hour 3 – 3.5 on Days 1 & 2<br>If <10kg, see section 3.12 of protocol for dosing
G-CSF<br>or<br>PEG G-CSF	Subcutaneous injection	G-CSF: 5mcg/kg daily<br>or<br>PEG G-CSF: 0.1mg/kg (max 6mg) x1 dose		Start on Day 6	For daily G-CSF: administer until ANC is at least ≥1000 post nadir

Observations

A: Physical exam
B: Ht, Wt, BSA
C: CBC w/differential
D: Blood chemistry
E: Urinalysis
F: PT/PTT/Fibrinogen[1]
G: Vital signs w/ pulse oximetry
H: Serum pregnancy[2]
I: Bone marrow aspirate[3]
J: CSF
K: EKG & ECHO
L: Radiologic exam[4]
M: Correlative Studies-blood

[1] Repeat weekly if abnormal until normalized
[2] For females of childbearing potential
[3] If bone marrow aspirate unattainable, bone marrow biopsy may be substituted
[4] Appropriate radiologic exam for patients with extramedullary involvement

Therapy Delivery Map **Ht:_______cm Wt:________kg BSA:________m²**

Protocol Day	Date Due	Date Given	IT MTX or IT Cytarabine ______mg	CPM ________mg	ETOP ________mg	Carfilzomib ________mg	☐ G-CSF _____mcg or ☐ PEG G-CSF ____mg	Observations
– 13 to 0			______mg					J
Screening	Procedures to be completed within 7 days prior to enrollment, with the exception of EKG, ECHO, bone marrow aspirate/biopsy, and any and/or radiologic disease evaluation, which can be performed within 14 days prior to enrollment.							A, B, C, D, E, F, G, H, I, K, L
1*				______mg	______mg	______mg		A, C, D, E, G, M
2				______mg	______mg	______mg		A, C, D, E, G, M
3				______mg	______mg			A, C, D, E, G, M
4				______mg	______mg			A, C, D, E, G
5				______mg	______mg			A, C, D, E, G
6							____mcg OR ____mg	
8						______mg	↓	A, C, D, F, G, M
9						______mg		G
15						______mg		A, C, D, F, G
16						______mg		G
22							Date of last dose of G-CSF___/___/___	A, C, D, F, G
28								A, C, D, F, G, I, L

***IV chemotherapy should begin within 72 hours of study enrollment.**

Patient Label

Cycle:_______Dose Level:________ (assigned at enrollment)
The dosing schedule for this study will consist of a 28-day cycle
See section 3.12 and 4.1 – 4.3 for laboratory criteria required to begin each treatment cycle.

Leukemia: Subsequent Cycles
(Carfilzomib twice weekly dosing)

Drug	Route & Time	Dose		Days	Important Notes
IT Chemotherapy<br>ALL: IT Methotrexate (MTX)<br>AML or MPAL: IT Cytarabine	Intrathecal	**IT Methotrexate**<br>0.5-0.99 yo: 6 mg<br>1-1.99 yo : 8 mg<br>2-2.99 yo : 10 mg<br>3-8.99 yo : 12 mg<br>≥9 yo: 15 mg	**IT Cytarabine**<br>0.5-0.99 yo: 20 mg<br>1-1.99 yo: 30 mg<br>2-2.99 yo: 50 mg<br>> 3 yo: 70 mg	1	Patient must be documented CNS1 or CNS2 prior to continuing on study treatment. Intrathecal chemotherapy can be given with bone marrow aspirate at end of previous cycle to avoid second anesthesia.
Pre- and post-hydration required: See section 6.1 for details.					
Dexamethasone 0.1mg/kg (max 4mg) IV/PO pre-medication may be required for certain patients. See section 6.1 and 6.4 for details.					
Cyclophosphamide (CPM)	IV<br>over 60 minutes	____mg/m2 or ____mg/kg if <10kg		1 - 5	Administer Hour 0 – 1<br>If <10kg, see section 3.12 of protocol for dosing
Etoposide (ETOP)	IV<br>over 120 minutes	____mg/m2 or ____mg/kg if <10kg		1 - 5	Administer Hour 1 – 3<br>If <10kg, see section 3.12 of protocol for dosing
Carfilzomib	IV<br>over 30 minutes	_______mg/m2 or ____mg/kg if <10kg		1 – 2<br>8 – 9<br>15 – 16	Administer Hour 3 – 3.5 on Days 1 & 2<br>If <10kg, see section 3.12 of protocol for dosing
G-CSF<br>or<br>PEG G-CSF	Subcutaneous injection	G-CSF: 5mcg/kg daily<br>or<br>PEG G-CSF: 0.1mg/kg (max 6mg) x1 dose		Start on Day 6	For daily G-CSF: administer until ANC is at least ≥1000 post nadir

Observations

A: Physical
B: Ht, Wt, BSA
C: CBC w/differential
D: Blood chemistry
E: Urinalysis
F: Vital signs w/ pulse oximetry
G: Serum pregnancy[1]
H: Bone marrow aspirate[2, 3]
I: CSF[3]
J: EKG & ECHO
K: Radiologic exam[3]
L: Correlative Studies-blood[5]

[1] For females of childbearing potential
[2]If bone marrow aspirate unattainable, bone marrow biopsy may be substituted
[3]Bone marrow and CSF may be done at same time to avoid second anesthesia
[4] Appropriate radiologic exam for patients with extramedullary involvement
[5] Cycle 2 only

Therapy Delivery Map **Ht:_______cm** **Wt:________kg** **BSA:________m²**

Protocol Day	Date Due	Date Given	IT MTX or IT Cytarabine _______mg	CPM ________mg	ETOP ________mg	Carfilzomib ________mg	☐ G-CSF ______mcg or ☐ PEG G-CSF _____mg	Observations
Date of IT chemo			_______mg					H, I
1**				______mg	______mg	______mg		A, B, C, D, E, F, G, K, L
2				______mg	______mg	______mg		F, L
3				______mg	______mg			
4				______mg	______mg			
5				______mg	______mg			
6							____mcg OR ____mg	
8						______mg	↓	A, C, D, F
9						______mg		F
15						______mg		A, C, D, F
16						______mg		F
28							Date of last dose of G-CSF:___/___/___	A, C, D, F, K

****Observations do not need to be repeated on Day 1 of the new cycle if they were completed within 72 hours of Day 1, unless otherwise specified by the protocol****

POETIC

Phase I study of carfilzomib in combination with cyclophosphamide and etoposide for children with relapsed and refractory solid tumors and leukemias

Patient Label

Cycle:___1___ Dose level:________ (assigned at enrollment)

Solid Tumors and Lymphomas: First cycle

The dosing schedule for this study will consist of a 28-day cycle **(Carfilzomib twice weekly dosing)**

See section 3.12 and 4.1 – 4.3 for hematologic/serum chemistries required to begin each treatment cycle.

Drug	Route & Time	Dose	Days	Important Notes
Methotrexate (MTX)	Intrathecal	**IT Methotrexate**<br>0.5-0.99 yo: 6 mg<br>1-1.99 yo : 8 mg<br>2-2.99 yo : 10 mg<br>3-8.99 yo : 12 mg<br>≥9 yo: 15 mg	1	**FOR NON-HODGKIN'S LYMPHOMA PATIENTS ONLY**<br>Intrathecal chemotherapy can be given during diagnostic lumbar puncture<br>Can be done with bone marrow, if indicated, to avoid second anesthesia
Pre- and post-hydration required: See section 6.1 for details.				
Dexamethasone 0.1mg/kg (max 4mg) IV/PO pre-medication required. See section 6.1 and 6.4 for details.				
Cyclophosphamide (CPM)	IV<br>over 60 minutes	______mg/m2 or _______mg/kg if <10kg	1 - 5	Administer Hour 0 – 1<br>If <10kg, see section 3.12 of protocol for dosing
Etoposide (ETOP)	IV<br>over 120 minutes	______mg/m2 or _______mg/kg if <10kg	1 - 5	Administer Hour 1 – 3<br>If <10kg, see section 3.12 of protocol for dosing
Carfilzomib	IV<br>over 30 minutes	_______mg/m2 or _______mg/kg if <10kg	1 – 2<br>8 – 9<br>15 – 16	Administer Hour 3 – 3.5 on Days 1 & 2<br>If <10kg, see section 3.12 of protocol for dosing
G-CSF<br>Or<br>PEG G-CSF	Subcutaneous injection	G-CSF: 5mcg/kg<br>or<br>PEG G-CSF: 0.1mg/kg, max 6mg	Start on Day 6	Administer G-CSF until ANC at least 1,000 post nadir; or<br>Administer PEG G-CSF x1 dose

Observations

A: Physical exam
B: Ht, Wt, BSA
C: CBC w/differential
D: Blood chemistry
E: Urinalysis
F: PT/PTT/Fibrinogen[1]
G: Vital signs w/ pulse oximetry
H: Serum pregnancy[2]
I: Tumor assessment
J: CSF[3]
K: EKG & ECHO
L: Correlative Studies-blood

[1] Repeat weekly if abnormal until normalized
[2] For females of childbearing potential
[3] **Non-Hodgkin's lymphoma patients ONLY**

Therapy Delivery Map **Ht:_______cm** **Wt:________kg** **BSA:________m2**

Protocol Day	Date Due	Date Given	IT MTX _____mg (for Non-Hodgkin's lymphoma ONLY)	CPM _____mg	ETOP _____mg	Carfilzomib _____mg	☐ G-CSF _____mcg or ☐ PEG G-CSF _____mg	Observations
– 13 to 0			______mg					J
Screening	Procedures to be completed within 7 days prior to enrollment, with the exception of EKG, ECHO, and tumor assessment, which can be performed within 14 days prior to enrollment.							A, B, C, D, E, F, G, H, I, K
1*				______mg	______mg	______mg		A, B, C, D, E, G, L
2				______mg	______mg	______mg		A, C, D, E, G, L
3				______mg	______mg			A, C, D, E, G, L
4				______mg	______mg			A, C, D, E, G
5				______mg	______mg			A, C, D, E, G
6							_____mcg OR _____mg	
8						______mg	↓	A, C, D, F, G, L
9						______mg		G
15						______mg		A, C, D, F, G
16						______mg		G
22							Date of last dose of G-CSF:___/___/___	A, C, D, F, G
28								A, C, D, F, G

*** IV chemotherapy should begin within 72 hours of study enrollment.**

Patient Label

Cycle:______ Dose level:________ (assigned at enrollment)

Solid Tumors and Lymphomas: Subsequent Cycles

The dosing schedule for this study will consist of a 28-day cycle. **(Carfilzomib twice weekly dosing)**

See section 3.12 and 4.1 – 4.3 for hematologic/serum chemistries required to begin each treatment cycle.

Drug	Route & Time	Dose	Days	Important Notes
Methotrexate (MTX)	Intrathecal	**IT Methotrexate**<br>0.5-0.99 yo: 6 mg<br>1-1.99 yo : 8 mg<br>2-2.99 yo : 10 mg<br>3-8.99 yo : 12 mg<br>≥9 yo: 15 mg	Prior to Day 1	**FOR NON-HODGKIN'S LYMPHOMA PATIENTS ONLY**<br>Intrathecal chemotherapy can be given during response assessment lumbar puncture<br>Can be done with Bone Marrow, if indicated, to avoid second anesthesia
Pre- and post-hydration required: See section 6.1 for details.				
Dexamethasone 0.1mg/kg (max 4mg) IV/PO pre-medication may be required for certain patients. See section 6.1 and 6.4 for details.				
Cyclophosphamide (CPM)	IV<br>over 60 minutes	______mg/m2 or _______mg/kg if <10kg	1 - 5	Administer Hour 0 – 1<br>If <10kg, see section 3.12 of protocol for dosing
Etoposide (ETOP)	IV<br>over 120 minutes	______mg/m2 or _______mg/kg if <10kg	1 - 5	Administer Hour 1 – 3<br>If <10kg, see section 3.12 of protocol for dosing
Carfilzomib	IV<br>over 30 minutes	_______mg/m2 or _______mg/kg if <10kg	1 – 2<br>8 – 9<br>15 – 16	Administer Hour 3 – 3.5 on Days 1 & 2<br>If <10kg, see section 3.12 of protocol for dosing
G-CSF<br>Or<br>PEG G-CSF	Subcutaneous injection	G-CSF: 5mcg/kg<br>or<br>PEG G-CSF: 0.1mg/kg, max 6mg	Start on Day 6	Administer G-CSF until ANC at least 1,000 post nadir; or<br>Administer PEG G-CSF x1 dose

Observations

A: Physical exam
B: Ht, Wt, BSA
C: CBC w/differential
D: Blood Chemistry
E: Urinalysis
F: PT/PTT/Fibrinogen
G: Vital signs w/ pulse oximetry
H: Serum pregnancy[1]
I: Tumor assessment[2]
J: CSF[3]
K: EKG & ECHO
L: Correlative Studies-blood[4]

[1] For females of childbearing potential
[2] Following cycles 2, 4, 6, then every 3 cycles unless otherwise clinically indicated; Include bone marrow evaluation if tumor with bone marrow involvement at the time of study enrollment.
[3] **Non-Hodgkin's lymphoma patients ONLY**
[4] Cycle 2 only

Therapy Delivery Map **Ht:_______cm** **Wt:________kg** **BSA:________m²**

Protocol Day	Date Due	Date Given	IT MTX _____mg (for Non-Hodgkin's lymphoma ONLY)	CPM _____mg	ETOP _____mg	Carfilzomib _____mg	☐ G-CSF _____mcg or ☐ PEG G-CSF _____mg	Observations
**Date of IT Chemo**			**______mg**					**J**
**1****				______mg	______mg	______mg		**A, B, C, D, E, G, H, L**
**2**				______mg	______mg	______mg		**G, L**
**3**				______mg	______mg			**G**
**4**				______mg	______mg			**G**
**5**				______mg	______mg			**G**
**6**							_____mcg OR _____mg	
**8**						______mg	↓	**A, C, D, G**
**9**						______mg	↓	**G**
**15**						______mg	↓	**A, C, D, G**
**16**						______mg	↓	**G**
**28**							**Date of last dose of G-CSF:___/___/___**	**A, C, D, G, I**

****Observations do not need to be repeated on Day 1 of the new cycle if they were completed within 72 hours of Day 1, unless otherwise specified by the protocol****
