# Supplementary material for: A Phase I Study of Carfilzomib with Cyclophosphamide and Etoposide in Relapsed and Refractory Leukemia and Solid Tumors
Source: Cancers (Basel). 2025 Sep 6;17(17):2924. doi: 10.3390/cancers17172924 (PMC12428389; doi:10.3390/cancers17172924)
Supplement: Supplementary file 1 [file cancers-17-02924-s001.zip › POE14-01 Publication Supplement_29Aug2025.pdf]

A phase I study of carfilzomib with cyclophosphamide and etoposide in relapsed and refractory leukemia and solid tumors

Supplement Tables

Contents

Table 5: Site Table 2

SAEs All Treated Patients All cycles (>5%) 2

AEs (Grade ≥3) Toxicity Evaluable Patients All cycles (>5%) 4

AEs (Grade ≥3) All Patients Treated All Cycles (>5%) 6

Table 5: Site Table

Site Name	n = 42
Alberta Children's Hospital	2
Arkansas Children's Hospital	8
Dana-Farber Cancer Institute	1
Memorial Sloan-Kettering Cancer Center	4
Penn State Hershey Children's Hospital	3
Phoenix Children's Hospital	10
Stanford University	6
UT Health Science Center at San Antonio	8

Table 5. Table summarizing the number of participants enrolled per site.

SAEs All Treated Patients All cycles (>5%)

Table 6: Serious Adverse Events (SAEs) by Dose Level in All Patients (n = 38) Across All Treatment Cycles

	Dose				
SAE	11mg/m^2 n=7	15mg/m^2 n=14	20mg/m^2 n=3	20/27mg/m^2 n=3	20/36mg/m^2 n=11
Febrile neutropenia	4 (57.1)	5 (35.7)	2 (66.7)	3 (100)	6 (54.5)
Anemia	1 (14.3)	0 (0)	1 (33.3)	0 (0)	1 (9.1)
Fever	1 (14.3)	0 (0)	1 (33.3)	0 (0)	1 (9.1)
Diarrhea	0 (0)	1 (7.1)	0 (0)	0 (0)	1 (9.1)
Hematuria	0 (0)	0 (0)	0 (0)	1 (33.3)	1 (9.1)
Hypertension	0 (0)	0 (0)	0 (0)	0 (0)	2 (18.2)
Hypotension	0 (0)	0 (0)	0 (0)	0 (0)	2 (18.2)
Lung infection	0 (0)	0 (0)	0 (0)	0 (0)	2 (18.2)
PRES*	0 (0)	1 (7.1)	0 (0)	0 (0)	1 (9.1)
Sepsis	1 (14.3)	0 (0)	0 (0)	0 (0)	1 (9.1)
Skin infection	0 (0)	0 (0)	0 (0)	0 (0)	2 (18.2)

PRES*, posterior reversible encephalopathy syndrome

Table 6. Number and percentage of treated subjects (n = 38) who experienced Serious Adverse Events (SAEs). SAEs are categorized by dose level and reported across all treatment cycles. Values in parentheses represent the proportion of subjects (%) experiencing each listed SAE for each dose level. Only SAEs reported in more than 5% of all subjects are included.

Table 7: Serious Adverse Events (SAEs) by Tumor Type in All Patients (n = 38) Across All Treatment Cycles

SAE	Tumor Type	
	Leukemia n=14	Solid Tumor n=24
Febrile neutropenia	5 (35.7)	15 (62.5)
Anemia	0 (0)	3 (12.5)
Fever	1 (7.1)	2 (8.3)
Diarrhea	0 (0)	2 (8.3)
Hematuria	0 (0)	2 (8.3)
Hypertension	0 (0)	2 (8.3)
Hypotension	0 (0)	2 (8.3)
Lung infection	0 (0)	2 (8.3)
PRES*	1 (7.1)	1 (4.2)
Sepsis	1 (7.1)	1 (4.2)
Skin infection	0 (0)	2 (8.3)

PRES*, posterior reversible encephalopathy syndrome

Table 7. Number and percentage of treated subjects (n = 38) who experienced Serious Adverse Events (SAEs). SAEs are categorized by tumor type and reported across all treatment cycles. Values in parentheses represent the proportion of subjects (%) experiencing each listed SAE for each tumor type. Only SAEs reported in more than 5% of all subjects are included.

AEs (Grade ≥3) Toxicity Evaluable Patients All cycles (>5%)

Table 8: Adverse Events (AEs) (Grade ≥3) by Dose Level in patients evaluable for toxicity (n = 31) Across All Treatment Cycles

	Dose				
AE Term	11mg/m^2 n=6	15mg/m^2 n=8	20mg/m^2 n=3	20/27mg/m^2 n=3	20/36mg/m^2 n=11
Platelet count decreased	6 (100)	8 (100)	3 (100)	3 (100)	11 (100)
White blood cell decreased	5 (83.3)	8 (100)	3 (100)	3 (100)	11 (100)
Lymphocyte count decreased	5 (83.3)	7 (87.5)	3 (100)	2 (66.7)	10 (90.9)
Anemia	5 (83.3)	7 (87.5)	3 (100)	2 (66.7)	9 (81.8)
Neutrophil count decreased	4 (66.7)	5 (62.5)	3 (100)	2 (66.7)	10 (90.9)
Hypokalemia	1 (16.7)	3 (37.5)	1 (33.3)	0 (0)	1 (9.1)
Hypoxia	0 (0)	3 (37.5)	0 (0)	0 (0)	3 (27.3)
Alanine aminotransferase increased	1 (16.7)	1 (12.5)	1 (33.3)	1 (33.3)	0 (0)
Febrile neutropenia	1 (16.7)	3 (37.5)	0 (0)	0 (0)	0 (0)
GGT increased	3 (50)	1 (12.5)	0 (0)	0 (0)	0 (0)
Aspartate aminotransferase increased	0 (0)	1 (12.5)	1 (33.3)	1 (33.3)	0 (0)
Blood bilirubin increased	0 (0)	1 (12.5)	1 (33.3)	0 (0)	1 (9.1)
Hypotension	1 (16.7)	0 (0)	0 (0)	1 (33.3)	1 (9.1)
Nausea	0 (0)	1 (12.5)	0 (0)	1 (33.3)	1 (9.1)
Abdominal pain	0 (0)	1 (12.5)	0 (0)	0 (0)	1 (9.1)
Back pain	1 (16.7)	1 (12.5)	0 (0)	0 (0)	0 (0)
Hyponatremia	1 (16.7)	0 (0)	0 (0)	0 (0)	1 (9.1)
Hypophosphatemia	0 (0)	1 (12.5)	0 (0)	0 (0)	1 (9.1)
Lung infection	0 (0)	1 (12.5)	0 (0)	1 (33.3)	0 (0)
Sinus tachycardia	0 (0)	0 (0)	0 (0)	1 (33.3)	1 (9.1)
Skin infection	0 (0)	1 (12.5)	0 (0)	0 (0)	1 (9.1)

Table 8. Number and percentage of toxicity-evaluable subjects (n = 31) who experienced Adverse Events (AEs) of Grade 3 or higher. AEs are categorized by dose level and reported across all treatment cycles. Values in parentheses represent the proportion of subjects (%) experiencing each listed adverse event for each dose level. Only adverse events reported in more than 5% of all subjects are included.

Table 9: Adverse Events (AEs) (Grade ≥3) by Tumor Type in patients evaluable for toxicity (n = 31) Across All Treatment Cycles

	Tumor Type	
AE Term	Leukemia n=8	Solid Tumor n=23
Platelet count decreased	8 (100)	23 (100)
White blood cell decreased	8 (100)	22 (95.7)
Lymphocyte count decreased	8 (100)	19 (82.6)
Anemia	8 (100)	18 (78.3)
Neutrophil count decreased	6 (75)	18 (78.3)
Hypokalemia	3 (37.5)	3 (13)
Hypoxia	2 (25)	4 (17.4)
Alanine aminotransferase increased	1 (12.5)	3 (13)
Febrile neutropenia	4 (50)	0 (0)
GGT increased	3 (37.5)	1 (4.3)
Aspartate aminotransferase increased	1 (12.5)	2 (8.7)
Blood bilirubin increased	1 (12.5)	2 (8.7)
Hypotension	1 (12.5)	2 (8.7)
Nausea	1 (12.5)	2 (8.7)
Abdominal pain	1 (12.5)	1 (4.3)
Back pain	2 (25)	0 (0)
Hyponatremia	1 (12.5)	1 (4.3)
Hypophosphatemia	1 (12.5)	1 (4.3)
Lung infection	1 (12.5)	1 (4.3)
Sinus tachycardia	0 (0)	2 (8.7)
Skin infection	1 (12.5)	1 (4.3)

Table 9. Number and percentage of toxicity-evaluable subjects (n = 31) who experienced Adverse Events (AEs) of Grade 3 or higher. AEs are categorized by tumor type and reported across all treatment cycles. Values in parentheses represent the proportion of subjects (%) experiencing each listed adverse event for each tumor type. Only adverse events reported in more than 5% of all subjects are included.

AEs (Grade ≥3) All Patients Treated All Cycles (>5%)

Table 10: Adverse Events (AEs) (Grade ≥3) by Dose Level in all patients (n = 38) Across All Treatment Cycles

	Dose				
AE Term	11mg/m^2 n=7	15mg/m^2 n=14	20mg/m^2 n=3	20/27mg/m^2 n=3	20/36mg/m^2 n=11
Platelet count decreased	7 (100)	12 (85.7)	3 (100)	3 (100)	11 (100)
White blood cell decreased	5 (71.4)	11 (78.6)	3 (100)	3 (100)	11 (100)
Anemia	6 (85.7)	12 (85.7)	3 (100)	2 (66.7)	9 (81.8)
Lymphocyte count decreased	5 (71.4)	10 (71.4)	3 (100)	2 (66.7)	10 (90.9)
Neutrophil count decreased	5 (71.4)	7 (50)	3 (100)	2 (66.7)	10 (90.9)
Febrile neutropenia	1 (14.3)	6 (42.9)	0 (0)	0 (0)	0 (0)
Hypokalemia	1 (14.3)	3 (21.4)	1 (33.3)	0 (0)	1 (9.1)
Hypoxia	0 (0)	3 (21.4)	0 (0)	0 (0)	3 (27.3)
Alanine aminotransferase increased	1 (14.3)	1 (7.1)	1 (33.3)	1 (33.3)	0 (0)
GGT increased	3 (42.9)	1 (7.1)	0 (0)	0 (0)	0 (0)
Aspartate aminotransferase increased	0 (0)	1 (7.1)	1 (33.3)	1 (33.3)	0 (0)
Back pain	2 (28.6)	1 (7.1)	0 (0)	0 (0)	0 (0)
Blood bilirubin increased	0 (0)	1 (7.1)	1 (33.3)	0 (0)	1 (9.1)
Hypotension	1 (14.3)	0 (0)	0 (0)	1 (33.3)	1 (9.1)
Lung infection	0 (0)	2 (14.3)	0 (0)	1 (33.3)	0 (0)
Nausea	0 (0)	1 (7.1)	0 (0)	1 (33.3)	1 (9.1)
Abdominal pain	0 (0)	1 (7.1)	0 (0)	0 (0)	1 (9.1)
Activated partial thromboplastin time prolonged	0 (0)	1 (7.1)	1 (33.3)	0 (0)	0 (0)
Hyponatremia	1 (14.3)	0 (0)	0 (0)	0 (0)	1 (9.1)
Hypophosphatemia	0 (0)	1 (7.1)	0 (0)	0 (0)	1 (9.1)
Sinus tachycardia	0 (0)	0 (0)	0 (0)	1 (33.3)	1 (9.1)
Skin infection	0 (0)	1 (7.1)	0 (0)	0 (0)	1 (9.1)

Table 10. Number and percentage of treated subjects (n = 38) who experienced Adverse Events (AEs) of Grade 3 or higher. AEs are categorized by dose level and reported across all treatment cycles. Values in parentheses represent the proportion of subjects (%) experiencing each listed adverse event for each dose level. Only adverse events reported in more than 5% of all subjects are included.

Table 11: Adverse Events (AEs) (Grade ≥3) by Tumor Type in all patients (n = 38) Across All Treatment Cycles

	Tumor Type	
AE Term	Leukemia n=14	Solid Tumor n=24
Platelet count decreased	12 (85.7)	24 (100)
White blood cell decreased	10 (71.4)	23 (95.8)
Anemia	13 (92.9)	19 (79.2)
Lymphocyte count decreased	10 (71.4)	20 (83.3)
Neutrophil count decreased	9 (64.3)	18 (75)
Febrile neutropenia	7 (50)	0 (0)
Hypokalemia	3 (21.4)	3 (12.5)
Hypoxia	2 (14.3)	4 (16.7)
Alanine aminotransferase increased	1 (7.1)	3 (12.5)
GGT increased	3 (21.4)	1 (4.2)
Aspartate aminotransferase increased	1 (7.1)	2 (8.3)
Back pain	3 (21.4)	0 (0)
Blood bilirubin increased	1 (7.1)	2 (8.3)
Hypotension	1 (7.1)	2 (8.3)
Lung infection	1 (7.1)	2 (8.3)
Nausea	1 (7.1)	2 (8.3)
Abdominal pain	1 (7.1)	1 (4.2)
Activated partial thromboplastin time prolonged	1 (7.1)	1 (4.2)
Hyponatremia	1 (7.1)	1 (4.2)
Hypophosphatemia	1 (7.1)	1 (4.2)
Sinus tachycardia	0 (0)	2 (8.3)
Skin infection	1 (7.1)	1 (4.2)

Table 11. Number and percentage of treated subjects (n = 38) who experienced Adverse Events (AEs) of Grade 3 or higher. AEs are categorized by tumor type and reported across all treatment cycles. Values in parentheses represent the proportion of subjects (%) experiencing each listed adverse event for each tumor type. Only adverse events reported in more than 5% of all subjects are included.
